# Risks of myocarditis, pericarditis, and cardiac arrhythmias associated with COVID-19 vaccination or SARS-CoV-2 infection

**DOI:** 10.1038/s41591-021-01630-0

**Published:** 2021-12-14

**Authors:** Martina Patone, Xue W. Mei, Lahiru Handunnetthi, Sharon Dixon, Francesco Zaccardi, Manu Shankar-Hari, Peter Watkinson, Kamlesh Khunti, Anthony Harnden, Carol A. C. Coupland, Keith M. Channon, Nicholas L. Mills, Aziz Sheikh, Julia Hippisley-Cox

**Affiliations:** 1 Nuffield Department of Primary Health Care Sciences, University of Oxford, Oxford, UK; 2 Wellcome Centre for Human Genetics, University of Oxford, Oxford, UK; 3 Leicester Real World Evidence Unit, Diabetes Research Centre, University of Leicester, Leicester, UK; 4 Usher Institute, University of Edinburgh, Edinburgh, UK; 5 Centre for Inflammation Research, University of Edinburgh, Edinburgh, UK; 6 School of Immunology and Microbial Sciences, King’s College London, London, UK; 7 Nuffield Department of Clinical Neurosciences, University of Oxford, Oxford, UK; 8 NIHR Biomedical Research Centre, Oxford University Hospitals NHS Trust, Oxford, UK; 9 Division of Primary Care, School of Medicine, University of Nottingham, Nottingham, UK; 10 British Heart Foundation Centre of Research Excellence, NIHR Oxford Biomedical Research Centre, University of Oxford, John Radcliffe Hospital, Oxford, UK; 11 BHF/University Centre for Cardiovascular Science, University of Edinburgh, Edinburgh, UK

**Keywords:** Epidemiology, Cardiovascular diseases

## Abstract

Although myocarditis and pericarditis were not observed as adverse events in coronavirus disease 2019 (COVID-19) vaccine trials, there have been numerous reports of suspected cases following vaccination in the general population. We undertook a self-controlled case series study of people aged 16 or older vaccinated for COVID-19 in England between 1 December 2020 and 24 August 2021 to investigate hospital admission or death from myocarditis, pericarditis and cardiac arrhythmias in the 1–28 days following adenovirus (ChAdOx1, *n* = 20,615,911) or messenger RNA-based (BNT162b2, *n* = 16,993,389; mRNA-1273, *n* = 1,006,191) vaccines or a severe acute respiratory syndrome coronavirus 2 (SARS-CoV-2) positive test (*n* = 3,028,867). We found increased risks of myocarditis associated with the first dose of ChAdOx1 and BNT162b2 vaccines and the first and second doses of the mRNA-1273 vaccine over the 1–28 days postvaccination period, and after a SARS-CoV-2 positive test. We estimated an extra two (95% confidence interval (CI) 0, 3), one (95% CI 0, 2) and six (95% CI 2, 8) myocarditis events per 1 million people vaccinated with ChAdOx1, BNT162b2 and mRNA-1273, respectively, in the 28 days following a first dose and an extra ten (95% CI 7, 11) myocarditis events per 1 million vaccinated in the 28 days after a second dose of mRNA-1273. This compares with an extra 40 (95% CI 38, 41) myocarditis events per 1 million patients in the 28 days following a SARS-CoV-2 positive test. We also observed increased risks of pericarditis and cardiac arrhythmias following a positive SARS-CoV-2 test. Similar associations were not observed with any of the COVID-19 vaccines, apart from an increased risk of arrhythmia following a second dose of mRNA-1273. Subgroup analyses by age showed the increased risk of myocarditis associated with the two mRNA vaccines was present only in those younger than 40.

## Abstract (web summary)

A self-controlled case series using individual-patient-level data from over 38 million people aged 16 years and over, reveals an increased risk of myocarditis within a week of receiving a first dose of ChAdOx1, BNT162b2 and mRNA-1273 vaccines, which was further increased after a second dose of either mRNA vaccine. SARS-CoV-2 infection was associated with even greater risk of myocarditis, as well as pericarditis and cardiac arrhythmia.

## Main

By the end of September 2021, more than 6.3 billion doses of COVID-19 vaccination had been administered worldwide^1^. Clinical trials of COVID-19 vaccines were underpowered to detect the rare adverse events that are important for risk–benefit evaluations and to inform clinical practice postvaccination. Therefore, identifying such rare adverse events is now a global scientific priority.

As of 4 November 2021, there have been 1,783 reports to the United States Vaccine Adverse Event Reporting System (VAERS) of cases of heart inflammation, namely myocarditis or pericarditis, among people aged 12–29 years who received COVID-19 vaccines, in particular following mRNA vaccination, that is, BNT162b2 and mRNA-1273 vaccines^2^. As of 9 July 2021, the European Medicines Agency (EMA) has reported 145 cases of myocarditis and 138 cases of pericarditis out of 177 million doses of the BNT162b2 vaccine, and 9 cases of myocarditis and 19 cases of pericarditis out of 20 million doses of the mRNA-1273 vaccine^3^. In Israel, 275 cases of myocarditis were reported between December 2020 and May 2021 among more than 5 million people vaccinated with the BNT162b2 vaccine^4^. No association between ChAdOx1 vaccine and myocarditis or pericarditis has been reported. The same reports showed that these events are more likely to occur in adolescent and young adults, mostly after the second dose. Evaluation of the risks of adverse events following vaccination or SARS-CoV-2 infection in different age groups provides crucial information to determine whether the risks from the vaccine outweighs the risks following a positive SARS-CoV-2 test.

In England, the vaccination campaign began on 8 December 2020 with the BNT162b2 vaccine followed by the ChAdOx1 vaccine on 4 January 2021. In the first phase, priority was given to the most vulnerable, in a schedule based primarily on age. The mRNA-1273 vaccine became available in England on 13 April 2021. Since 7 April 2021, ChAdOx1 vaccine has not been recommended for individuals younger than 30 years of age, and since 7 May 2021 for individuals younger than 40 years of age.

The English National Immunisation (NIMS) Database of COVID-19 vaccination includes data on vaccine type, date and doses for all people vaccinated in England. We linked NIMS, at individual patient level, to national data for mortality, hospital admissions and SARS-CoV-2 infection data to examine the associations between the first and second dose of ChAdOx1, BNT162b2 or mRNA-1273 vaccines and cardiac adverse events: myocarditis, pericarditis or cardiac arrhythmias. We used the same population to investigate the associations between a positive SARS-CoV-2 test (before or after vaccination) as a secondary exposure and the same cardiac adverse events. We also assessed risks for the same outcomes following vaccination or a SARS-CoV-2 positive test in younger persons (<40 years old). Incidence rate ratios, the rate of hospital admission or death from each outcome in risk periods after vaccination or a positive test relative to baseline periods, were estimated using self-controlled case series (SCCS) methodology^5,6^.

## Results

A total of 38,615,491 adults had been vaccinated with at least one dose of ChAdOx1 (*n* = 20,615,911), BNT162b2 (*n* = 16,993,389) or mRNA-1273 (*n* = 1,006,191) in England between 1 December 2020 and 24 August 2021 (Table 1). Of these, 32,095,748 had received two doses of either ChAdOx1 (*n* = 19,754,224, 95.8%), BNT162b2 (*n* = 11,972,733, 70.5%) or mRNA-1273 (*n* = 368,791, 36.7%). Individuals receiving the ChAdOx1 and BNT162b2 vaccine were older, on average, than those receiving the mRNA-1273 vaccine, as expected given that the mRNA-1273 vaccine roll-out began in April 2021 in the United Kingdom, when higher priority risk groups (including older people) had already received their vaccine.

**Table 1:** Baseline demographic characteristics of people receiving either ChAdOx1, BNT162b2 or mRNA-1273 vaccines or testing positive for SARS-CoV-2 virus (before or after vaccination), in England between 1 December 2020 and 24 August 2021. Data are presented as column % (counts)

	ChAdOx1	BNT162b2	mRNA-1273	ChAdOx1	BNT162b2	mRNA-1273	Positive SARS-CoV-2 test (amongst total vaccinated)
	One dose (at least) (*n* = 38,615,491)			Two doses (*n* = 32,095,748)			
Total number of people	20,615,911	16,993,389	1,006,191	19,754,224	11,972,733	368,791	3,028,867
**Sex**							
Women	43.3 (8,918,403)	42.6 (7,233,091)	29.9 (300,567)	43.3 (8,559,325)	47.2 (5,650,542)	33.9 (125,120)	45.7 (1,385,137)
Men	34.9 (7,191,428)	31.8 (5,401,842)	28.5 (286,893)	34.9 (6,900,964)	32.6 (3,906,666)	26.4 (97,524)	32.2 (974,389)
Not recorded	21.9 (4,506,080)	25.6 (4,358,456)	41.6 (418,731)	21.7 (4,293,935)	20.2 (2,415,525)	39.6 (146,147)	22.1 (669,341)
**Age**							
Mean age (s.d.)	55.2 (14.8)	47.8 (21.7)	32.3 (9.4)	55.4 (14.7)	55.5 (20.4)	39.6 (7.3)	44.5 (17.8)
16–29 years	5.2 (1,064,443)	25.2 (4,285,600)	41.4 (416,982)	5.0 (988,291)	10.4 (1,244,710)	6.9 (25,382)	24.4 (738,170)
30–39 years	7.8 (1,598,406)	23.2 (3,945,405)	36.4 (366,327)	7.6 (1,494,285)	20.7 (2,475,091)	43.8 (161,412)	19.0 (574,710)
40+ years	87.1 (17,953,062)	51.6 (8,762,384)	21.2 (222,882)	87.4 (17,271,648)	68.9 (8,252,932)	49.3 (181,997)	56.6 (1,715,987)
**Ethnicity**							
White	55.5 (11,449,387)	52.3 (8,887,419)	40.1 (403,362)	55.8 (11,019,453)	57.8 (6,921,753)	43.0 (158,719)	52.6 (1,593,727)
Indian	1.5 (319,328)	1.7 (288,641)	0.7 (7,406)	1.5 (304,100)	1.8 (217,910)	0.8 (2,975)	2.4 (72,852)
Pakistani	1.0 (214,193)	1.1 (183,917)	0.7 (6,724)	1.0 (191,755)	0.9 (105,414)	0.4 (1,474)	2.1 (64,153)
Bangladeshi	0.4 (81,004)	0.4 (62,128)	0.3 (3,287)	0.4 (74,743)	0.3 (34,927)	0.2 (684)	0.7 (21,585)
Other Asian	0.7 (137,542)	0.8 (134,649)	0.7 (6,648)	0.7 (129,169)	0.8 (90,508)	0.7 (2,488)	1.1 (31,933)
Black Caribbean	0.5 (99,666)	0.4 (62,101)	0.3 (2,734)	0.5 (91,098)	0.4 (47,767)	0.2 (664)	0.5 (14,152)
Black African	0.7 (149,358)	0.7 (115,282)	0.6 (5,929)	0.7 (134,635)	0.6 (73,472)	0.5 (1,679)	0.9 (27,757)
Chinese	0.3 (53,279)	0.3 (43,014)	0.3 (3,261)	0.3 (51,338)	0.3 (30,662)	0.5 (1,691)	0.2 (4,585)
Other ethnic group	1.8 (366,361)	1.9 (321,150)	1.9 (19,313)	1.7 (342,040)	1.7 (208,462)	1.8 (6,795)	2.3 (69,725)
Ethnicity not recorded	37.6 (7,745,793)	40.6 (6,895,088)	54.4 (547,527)	37.5 (7,415,893)	35.4 (4,241,858)	52.0 (191,622)	37.3 (1,128,398)
**Number of doses**							
One dose only	4.1 (835,832)	29.5 (5,007,083)	63.3 (637,335)	–	–	–	25.1 (759,352)
**Cardiac inflammation in the previous 2** **years**^**a**^							
Previous myocarditis	<0.1 (1,840)	<0.1 (1,485)	<0.1 (56)	<0.1 (1,747)	<0.1 (1,220)	<0.1 (15)	<0.1 (451)
Previous pericarditis	<0.1 (1,849)	<0.1 (1,508)	<0.1 (31)	<0.1 (1,771)	<0.1 (1,285)	<0.1 (8)	<0.1 (346)
Previous cardiac arrhythmia	2.6 (538,564)	2.9 (500,295)	0.3 (2,969)	2.6 (505,794)	3.9 (466,724)	0.3 (1,005)	3.1 (92,985)

^a^Two years before 1 December 2020

Amongst those with at least one dose, there were 3,028,867 (7.8%) individuals who had a SARS-CoV-2 positive test. Of these, 2,315,669 (6.0%) individuals tested positive before vaccination; while 713,198 (1.8%) and 298,315 (0.7%) tested positive after the first and second vaccine doses, respectively. Table 1 shows the characteristics of the study population, stratified by vaccine type and dose, and of those who tested positive for SARS-CoV-2.

**Fig. 1:**
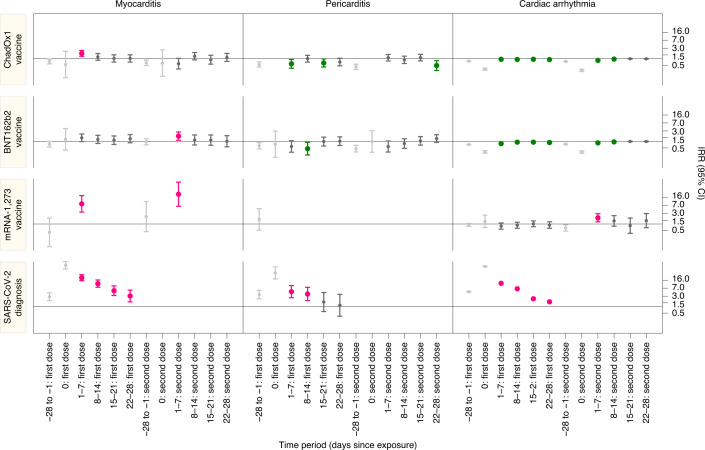
IRRs with 95% CIs for cardiac adverse events following each exposure. IRRs are presented for predefined risk periods (0, 1–7, 8–14, 15–21 and 22–28 days) after first or second dose of ChAdOx1, BNT162b2 and mRNA-1273 vaccines and a SARS-CoV-2 positive test for the prerisk period (28 days before exposure). Horizontal bold line in each panel indicates 1.

During the study period there were 1,615 and 1,574 admissions or deaths related to myocarditis and pericarditis, respectively (14 patients had both), and 385,508 related to cardiac arrhythmias. The characteristics of individuals with myocarditis, pericarditis and cardiac arrhythmias in the 1–28 days postvaccination differed by condition and according to the vaccine administered (Table 2). Supplementary Table 1 shows the characteristics of patients who died for the individual outcomes in the 1–28 days following a first or second dose of COVID-19 vaccine or SARS-CoV-2 infection. Table 3 and Fig. 1 show the number of patients with outcome events in each exposure time period and the incidence rate ratios (IRRs) and 95% CIs for outcomes in the exposure risk periods.

**Table 2:** Demographic characteristics of patients who experienced the individual outcomes in the 1–28 days following a first or second dose of COVID-19 vaccine or SARS-CoV-2 infection amongst the vaccinated population in England from 1 December 2020 to 24 August 2021 (cells with an asterisk are suppressed)

	Myocarditis							Pericarditis						
	1–28 days post first dose			1–28 days post second dose			1–28 days post test	1–28 days post first dose			1–28 days post second dose			1-28 days post test
	ChAdOx1nCoV-19 vaccine	BNT162b2 mRNA vaccine	mRNA-1273	ChAdOx1nCoV-19 vaccine	BNT162b2 mRNA vaccine	mRNA-1273	Positive SARS-CoV-2 test	ChAdOx1nCoV-19 vaccine	BNT162b2 mRNA vaccine	mRNA-1273	ChAdOx1nCoV-19 vaccine	BNT162b2 mRNA vaccine	mRNA-1273	Positive SARS-CoV-2 test
Total number of people	142	94	9	84	64	*	134	102	59	*	117	75	0	27
**Sex**														
Women	40.8 (58)	50.0 (47)	*	27.4 (23)	42.2 (27)	*	39.6 (53)	26.5 (27)	37.3 (22)	0	27.4 (32)	30.7 (23)	0	44.4 (12)
Men	58.5 (83)	50.0 (47)	*	71.4 (60)	57.8 (37)	*	60.4 (81)	72.5 (74)	62.7 (37)	*	72.6 (85)	69.3 (52)	0	55.6 (15)
Not recorded	0.7 (1)	0	0	1.2 (1)	0	0	0	1.0 (1)	0	0	0	0	0	0
**Age**														
Mean age (s.d.)	58.1 (18.2)	55.2 (22.0)	26.0 (9.9)	54.8 (18.3)	61.0 (22.8)	32.5 (10.7)	62.2 (17.0)	57.4 (13.8)	56.7 (20.1)	28.0 (5.3)	57.7 (16.6)	63.2 (18.7)	-	54.7 (14.5)
16–29 years	6.3 (9)	11.7 (11)	66.7 (6)	11.9 (10)	12.5 (8)	*	5.2 (7)	*	10.2 (6)	*	7.7 (9)	*	0	*
29–39 years	8.5 (12)	23.4 (22)	*	13.1 (11)	15.6 (10)	*	7.5 (10)	*	16.9 (10)	*	6.8 (8)	*	0	*
40+ years	85.2 (121)	64.9 (61)	*	75.0 (63)	71.9 (46)	*	87.3 (117)	93.1 (95)	72.9 (43)	0	85.5 (100)	86.7 (65)	0	88.9 (24)
**Number of doses**														
First dose only	44.4 (63)	52.1 (49)	100.0 (9)	–	–	–	19.4 (26)	17.6 (18)	32.2 (19)	*	–	–	–	22.2 (6)
**SARS-CoV-2**														
Positive SARS-CoV-2 test before vaccine	12.7 (18)	7.4 (7)	*	10.7 (9)	*	0	79.1 (106)	10.8 (11)	11.9 (7)	0	9.4 (11)	*	0	70.4 (19)

	Arrythmia													
**Sex**														
Total number of people	24,225	18,359	156	23,019	20,947	48	8,940							
Women	48.1 (11,651)	47.2 (8,655)	50.0 (78)	47.2 (10,872)	47.3 (9,907)	60.4 (29)	43.8 (3,913)							
Men	51.9 (12,568)	52.8 (9,699)	50.0 (78)	52.8 (12,143)	52.7 (11,038)	39.6 (19)	56.2 (5,026)							
Not recorded	<0.1 (6)	<0.0 (5)	0	<0.1 (4)	<0.1 (2)	0	<0.1 (1)							
**Age**														
Mean age (s.d.)	70.1 (16.2)	72.9 (18.1)	35.0 (11.4)	70.1 (15.3)	76.0 (14.5)	43.4 (9.5)	65.7 (18.0)							
18–29 years	2.1 (498)	4.4 (810)	35.9 (56)	1.8 (416)	1.5 (315)	*	3.7 (333)							
29–39 years	2.8 (674)	4.5 (829)	33.63 (52)	2.5 (574)	2.3 (487)	33.3 (16)	6.0 (535)							
40+ years	95.2 (23,053)	91.1 (16,720)	30.8 (48)	95.7 (22,029)	96.2 (20,145)	62.5 (30)	90.3 (8,072)							
**Number of doses**														
First dose only	18.5 (4,475)	20.6 (3,792)	60.3 (94)	–	–	–	17.0 (1,519)							
**SARS-CoV-2**														
Positive SARS-CoV-2 test before vaccine	6.6 (1,559)	3.8 (694)	9.0 (14)	5.5 (1,269)	2.8 (580)	0	75.6 (6,761)

**Table 3:** IRR (95% CI) for individual outcomes in predefined risk periods immediately before and after exposure to vaccination and before and after a positive SARS-CoV-2 test result, adjusted for calendar time from 1 December 2020 to 24 August 2021 (cells with an asterisk are suppressed). n/a, not applicable; pyrs, person-years

	ChAdOx1nCoV-19 vaccine			BNT162b2 mRNA vaccine			mRNA-1273 vaccine			Positive SARS-CoV-2 test		
	Events	pyrs	IRR (95% CI)	Events	pyrs	IRR (95% CI)	Events	pyrs	IRR (95% CI)	Events	pyrs	IRR (95% CI)
**Myocarditis**												
Baseline	550	409.0	1.00	398	307.7	1.00	22	20.4	1.00	119	216.7	1.00
−28 to −1 days: first dose/positive test	81	70.4	0.74 (0.58, 0.95)	49	49.0	0.75 (0.55, 1.01)	*	*	0.41 (0.09, 1.87)	32	21.0	2.84 (1.89, 4.28)
Day 0: first dose/positive test	*	*	0.54 (0.14, 2.19)	*	*	1.24 (0.40, 3.88)	*	*	n/a	36	0.8	78.21 (52.90, 115.62)
1–7 days: first dose/positive test	47	17.6	1.76 (1.29, 2.42)	27	12.5	1.45 (0.97, 2.17)	7	0.7	8.38 (3.53, 19.91)	68	5.8	21.08 (15.34, 28.96)
8–14 days: first dose/positive test	35	17.6	1.22 (0.85, 1.74)	23	12.5	1.23 (0.80, 1.90)	*	*	n/a	37	5.9	11.29 (7.70, 16.57)
15–21 days: first dose/positive test	30	17.6	1.03 (0.71, 1.51)	21	12.4	1.14 (0.73, 1.78)	*	*	n/a	18	6.0	5.36 (3.24, 8.89)
22–28 days: first dose/positive test	30	17.5	1.03 (0.71, 1.51)	23	12.0	1.33 (0.86, 2.04)	*	*	n/a	11	6.1	3.08 (1.65, 5.75)
−28 to −1 days: second dose	58	58.3	0.65 (0.49, 0.87)	48	33.3	0.96 (0.70, 1.32)	*	*	2.19 (0.45, 10.69)			
Day 0: second dose	*	*	0.64 (0.16, 2.56)	*	*	n/a	*	*	n/a			
1–7 days: second dose	13	14.8	0.60 (0.34, 1.04)	23	9.3	1.75 (1.13, 2.70)	*	*	23.10 (6.46, 82.56)			
8–14 days: second dose	28	14.8	1.31 (0.88, 1.93)	15	9.3	1.16 (0.69, 1.97)	*	*	n/a			
15–21 days: second dose	19	14.8	0.91 (0.57, 1.45)	14	9.3	1.14 (0.66, 1.97)	*	*	n/a			
22–28 days: second dose	24	14.8	1.16 (0.76, 1.76)	12	9.1	1.01 (0.57, 1.82)	*	*	n/a			
1–28 days: first dose/positive test	142	70.2	1.29 (1.05, 1.58)	94	49.4	1.31 (1.03, 1.66)	9	2.9	2.97 (1.34, 6.58)	134	23.9	9.76 (7.51, 12.69)
1–28 days: second dose	84	59.2	1.00 (0.78, 1.27)	64	37.1	1.30 (0.98, 1.72)	*	*	9.84 (2.69, 36.03)			
**Pericarditis**												
Baseline	581	405.2	1.00	414	285.9	1.00	12	11.2	1.00	95	115.4	1.00
−28 to −1 days: first dose/positive test	64	70.7	0.54 (0.42, 0.71)	42	47.2	0.63 (0.45, 0.87)	*	*	1.62 (0.52, 5.07)	29	10.0	3.57 (2.30, 5.55)
Day 0: first dose/positive test	*	*	n/a	*	*	0.74 (0.18, 2.98)	*	*	n/a	11	0.4	35.04 (18.47, 66.46)
1–7 days: first dose/positive test	19	17.7	0.59 (0.37, 0.94)	11	12.0	0.59 (0.32, 1.07)	*	*	n/a	11	2.8	4.85 (2.56, 9.18)
8–14 days: first dose/positive test	34	17.7	1.00 (0.70, 1.44)	9	12.0	0.46 (0.24, 0.90)	*	*	n/a	9	2.9	3.81 (1.90, 7.63)
15–21 days: first dose/positive test	23	17.6	0.64 (0.42, 0.99)	19	11.9	0.98 (0.62, 1.57)	*	*	n/a	*	*	1.63 (0.59, 4.45)
22–28 days: first dose/positive test	26	17.6	0.71 (0.47, 1.06)	20	11.6	1.02 (0.65, 1.61)	*	*	n/a	*	*	1.15 (0.36, 3.66)
−28 to −1 days: second dose	58	61.0	0.43 (0.32, 0.57)	35	36.5	0.47 (0.33, 0.67)	*	*	n/a			
Day 0: second dose	*	*	n/a	*	*	0.99 (0.32, 3.09)	*	*	n/a			
1–7 days: second dose	37	15.6	1.12 (0.79, 1.58)	12	10.1	0.58 (0.33, 1.04)	*	*	n/a			
8–14 days: second dose	29	15.6	0.89 (0.61, 1.31)	16	10.1	0.80 (0.48, 1.32)	*	*	n/a			
15–21 days: second dose	36	15.6	1.13 (0.80, 1.60)	21	10.1	1.05 (0.67, 1.64)	*	*	n/a			
22–28 days: second dose	15	15.5	0.49 (0.29, 0.82)	26	10.1	1.34 (0.89, 2.02)	*	*	n/a			
1–28 days: first dose/positive test	102	70.6	0.74 (0.59, 0.92)	59	47.4	0.77 (0.58, 1.02)	*	*	1.64 (0.45, 5.94)	27	11.7	2.79 (1.80, 4.32)
1–28 days: second dose	117	62.4	0.91 (0.73, 1.13)	75	40.4	0.93 (0.72, 1.21)	*	*	n/a			
**Cardiac arrhythmia**												
Baseline	118,356	88,084.1	1.00	113,968	81,778.6	1.00	1,221	867.6	1.00	15,119	24,323.4	1.00
−28 to −1 days: first dose/positive test	17,562	15,500.4	0.78 (0.76, 0.79)	12,767	13,158.8	0.72 (0.70, 0.73)	184	126.8	0.90 (0.77, 1.05)	6,618	2,223.6	4.82 (4.68, 4.97)
Day 0: first dose/positive test	289	553.9	0.33 (0.30, 0.38)	232	497.0	0.33 (0.29, 0.37)	9	4.5	1.32 (0.69, 2.54)	3,847	86.7	69.83 (67.32, 72.43)
1–7 days: first dose/positive test	5,873	3,872.1	0.95 (0.92, 0.97)	3,958	3,471.4	0.79 (0.76, 0.81)	38	31.6	0.80 (0.58, 1.11)	4,593	613.3	11.73 (11.33, 12.14)
8–14 days: first dose/positive test	5,981	3,867.0	0.93 (0.90, 0.95)	4,837	3,458.9	0.92 (0.89, 0.95)	40	31.4	0.88 (0.64, 1.21)	2,643	624.7	6.57 (6.30, 6.85)
15–21 days: first dose/positive test	6,243	3,863.0	0.95 (0.92, 0.97)	4,971	3,404.5	0.92 (0.90, 0.95)	43	32.1	1.02 (0.75, 1.38)	966	638.8	2.30 (2.15, 2.45)
22–28 days: first dose/positive test	6,128	3,857.0	0.92 (0.89, 0.94)	4,593	3,150.5	0.89 (0.86, 0.91)	35	29.1	0.90 (0.64, 1.26)	738	653.8	1.67 (1.55, 1.80)
−28 to −1 days: second dose	18,595	13,728.4	0.75 (0.74, 0.77)	15,010	10,907.1	0.75 (0.74, 0.77)	38	43.2	0.66 (0.47, 0.92)			
Day 0: second dose	265	502.4	0.30 (0.26, 0.33)	257	444.6	0.32 (0.29, 0.37)	*	*	n/a			
1–7 days: second dose	5,236	3,517.4	0.84 (0.82, 0.87)	4,737	3,112.3	0.85 (0.83, 0.88)	22	11.2	1.93 (1.25, 2.96)			
8–14 days: second dose	5,931	3,517.4	0.97 (0.94, 0.99)	5,288	3,112.3	0.95 (0.92, 0.98)	13	11.2	1.39 (0.80, 2.42)			
15–21 days: second dose	6,019	3,517.4	1.00 (0.97, 1.03)	5,495	3,112.3	0.99 (0.96, 1.02)	6	11.2	0.84 (0.37, 1.89)			
22–28 days: second dose	5,833	3,510.8	0.99 (0.97, 1.02)	5,427	3,098.4	0.99 (0.96, 1.02)	7	9.8	1.43 (0.67, 3.03)			
1–28 days: first dose/positive test ﻿	24,225	15,459.0	0.94 (0.93, 0.96)	18,359	13,485.4	0.89 (0.87, 0.90)	156	124.1	0.90 (0.76, 1.06)	8,940	2,530.5	5.35 (5.21, 5.50)
1–28 days: second dose	23,019	14,063.1	0.95 (0.94, 0.96)	20,947	12,435.3	0.95 (0.93, 0.96)	48	43.4	1.46 (1.08, 1.98)

### Myocarditis

Of the 38,615,491 vaccinated individuals included in our study, 1,615 (0.004%) were admitted to hospital with, or died from, myocarditis at any time in the study period (either before or after vaccination); 397 (0.001%) of these occurred in the 1-28 days post any dose of vaccine. Of the 1,615 who were admitted or died, 359 (22.2%) had a SARS-CoV-2 positive test, with 287 (17.8%) of these being before vaccination. There were 114 deaths with myocarditis recorded on the death certificate as a cause of death (23 had a SARS-CoV-2 positive test). Of those who have been admitted with, or died from, myocarditis in the 1-28 days postvaccination, 12.7% (18) and 10.7% (9) had a positive SARS-CoV-2 test before the first and second dose ChAdOx1 vaccine, respectively, and 7.4% (7) before the first dose of BNT162b2 vaccine (Table 2).

There was an increased risk of myocarditis at 1–7 days following the first dose of ChAdOx1 (IRR 1.76; 95% CI 1.29, 2.42), BNT162b2 (IRR 1.45, 95% CI 0.97, 2.12) and mRNA-1273 (IRR 8.38, 95% CI 3.53, 19.91), and the second dose of BNT162b2 (IRR 1.75, 95% CI 1.13, 2.70) and mRNA-1273 (IRR 23.10, 95% CI 6.46, 82.56). There was an increased risk of myocarditis at 1–7 days (IRR 21.08, 95% CI 15.34, 28.96), 8–14 days (IRR 11.29, 95% CI 7.70, 16.57), 15–21 days (IRR 5.36, 95% CI 3.24, 8.89) and 21–28 days (IRR 3.08, 95%CI 1.65, 5.75) following a positive test.

Over the 1–28 days postvaccination, we observed an association with the first dose of ChAdOx1 (IRR 1.29, 95% CI 1.05, 1.58), BNT162b2 (IRR 1.31, 95% CI 1.03, 1.66) and mRNA-1273 (IRR 2.97; 95% CI 1.34, 6.58). Following a second dose, the increased risk was much higher with mRNA-1273 (IRR 9.84, 95% CI 2.69, 36.03) compared with BNT162b2 (IRR 1.30, 95% CI 0.98, 1.72). The risk of myocarditis was increased in the 1–28 days following a SARS-CoV-2 positive test (IRR 9.76, 95% CI 7.51, 12.69).

### Pericarditis

Of the 38,615,491 vaccinated individuals included in our study, 1,574 (0.004%) were admitted to hospital with, or died from, pericarditis at any time in the study period (either before or after vaccination); 356 (0.001%) of these occurred in the 1-28 days after any dose of vaccine. Of the 1,574 who were admitted or died, 188 (11.9%) had a SARS-CoV-2 positive test, with 154 (9.8%) of these being before vaccination. There were 31 deaths with pericarditis recorded on the death certificate as cause of death (6 had a SARS-CoV-2 positive test). Table 2 shows the percentages of patients with pericarditis events in the risk period who had a positive SARS-CoV-2 test before vaccination by vaccine type and dose.

There were reduced risks of pericarditis after a first dose of ChAdOx1 (IRR 0.59; 95% CI 0.37, 0.94 at 1–7 days, IRR 0.64; 95% CI 0.42, 0.99 at 15–21 days), of BNT162b2 (IRR 0.46; 95% CI 0.24, 0.90 at 8–14 days) and following a second dose of ChAdOx1 (IRR 0.49; 95% CI: 0.29, 0.82 at 22–28 days). There were insufficient numbers of events to evaluate associations with the mRNA-1273 vaccine by week. There was an increased risk of hospital admission or death for pericarditis at 1–7 days (IRR 4.85, 95% CI 2.56, 9.18) and 8–14 days (IRR 3.81, 95% CI 1.90, 7.63) following a SARS-CoV-2 positive test.

Over the 1–28 days postvaccination, we observed a decreased risk of pericarditis following the first dose of ChAdOx1 (IRR 0.74, 95%CI 0.59, 0.92), in contrast with an increased risk in the 1–28 days following a SARS-CoV-2 positive test (IRR 2.79, 95% CI 1.80, 4.32). No association was observed with the BNT162b2 or mRNA-1273 vaccine.

### Cardiac arrhythmia

Of the 38,615,491 vaccinated individuals included in our study, 385,508 (1.0%) were admitted to hospital with or died from cardiac arrhythmia at any time in the study period (either before or after vaccination); 86,754 (0.2%) of these occurred in the 1-28 days after any dose of vaccine. Of those who were admitted or died 39,897 (10.3%) had a SARS-CoV-2 positive test, with 29,694 (7.7%) having a positive test before vaccination. There were 7,795 deaths with cardiac arrhythmia recorded as the cause of death (1,108 had a SARS-CoV-2 positive test). Table 2 shows the percentages of patients with cardiac arrhythmia events in the risk period who had a positive SARS-CoV-2 test before vaccination by vaccine type and dose.

There were decreased risks of cardiac arrhythmia after the first dose of ChAdOx1 (IRR 0.95, 95% CI 0.92, 0.97 at 1–7 days and over subsequent periods) and BNT162b2 (IRR 0.79, 95% CI 0.76, 0.81 at 1–7 days and over subsequent periods) and following a second dose of ChAdOx1 (IRR 0.84, 95% CI 0.82, 0.87 at 1–7 days; IRR 0.97, 95% CI 0.94, 0.99 at 8–14 days) and of BNT162b2 (IRR 0.85, 95% CI 0.83, 0.88 at 1–7 days; IRR 0.95, 95% CI 0.92, 0.98 at 8–15 days). There was an increased risk of cardiac arrhythmia following a second dose of mRNA-1273 (IRR 1.93, 95% CI 1.25, 2.96 at 1–7 days) and at 1–7 days (IRR 11.73, 95% CI 11.33, 12.14), 8–14 days (IRR 6.57, 95%CI 6.30, 6.85), 15–21 days (IRR 2.30, 95% CI 2.15, 2.45) and 21–28 days (IRR 1.67, 95% CI 1.55, 1.80) following a SARS-CoV-2 positive test.

Over the 1–28 days post vaccination, we found a decreased risk of cardiac arrhythmia associated with a first dose of ChAdOx1 (IRR 0.94, 95% CI 0.93, 0.96) and BNT162b2 (IRR 0.89, 95% CI 0.87, 0.90) and following a second dose of ChAdOx1 (IRR 0.95, 95% CI 0.94, 0.96) and BNT162b2 (IRR 0.95, 95% CI 0.93, 0.96). There was an increased risk of cardiac arrhythmia following a second dose of mRNA-1273 (IRR 1.46, 95% CI, 1.08, 1.98) and a SARS-CoV-2 positive test (IRR 5.35, 95% CI 5.21, 5.50).

### Subgroup analyses by age group and sex

Table 4 shows the IRRs for the outcomes in the overall 1–28 day risk periods before and after each exposure by sex and in those aged under 40 years or 40 years and older. Supplementary Tables 2 and 3a show the IRRs estimated for each week in the 1–28 days following exposure in these subgroups. Whilst the findings generally mirrored those reported in the overall 1–28 day period in each subgroup, given the small numbers of events in some weeks, care is needed in the interpretation. Here, we report the results of the subgroup analyses only for myocarditis.

**Table 4:** IRRs (95% CI) by age group (aged 40 years or younger, older than 40 years) and sex (women and men) for the outcomes in predefined risk periods immediately before and after exposure to vaccination and before and after a positive SARS-CoV-2 test result, adjusted for calendar time from 1 December 2020 to 24 August 2021 (cells with an asterisk are suppressed)

		ChAdOx1nCoV-19 vaccine		BNT162b2 mRNA vaccine		mRNA-1273 vaccine		Positive SARS-CoV-2 test	
	Time period	events	IRR (95% CI)	events	IRR (95% CI)	events	IRR (95% CI)	events	IRR (95% CI)
**Age** <**40** **years**									
**Myocarditis**	Baseline	95	1.00	120	1.00	16	1.00	37	1.00
	−28 to −1 days: first dose/positive test	8	0.36 (0.17, 0.77)	15	0.95 (0.54, 1.66)	*	0.76 (0.17, 3.50)	7	1.75 (0.76, 4.05)
	Day 0: first dose/positive test	*	n/a	*	3.29 (0.80, 13.45)	*	n/a	5	32.71 (12.49, 85.68)
	1–28 days: first dose/positive test	21	0.92 (0.55, 1.55)	33	1.83 (1.20, 2.79)	8	3.89 (1.60, 9.44)	17	4.06 (2.21, 7.45)
	−28 to −1 days: second dose	5	0.40 (0.16, 1.01)	7	0.93 (0.42, 2.09)	*	4.82 (0.82, 28.17)		
	Day 0: second dose	*	n/a	*	n/a	*	n/a		
	1–28 days: second dose	21	1.50 (0.88, 2.55)	18	3.40 (1.91, 6.04)	*	20.71 (4.02, 106.68)		
**Pericarditis**	Baseline	57	1.00	107	1.00	11	1.00	26	1.00
	−28 to −1 days: first dose/positive test	7	0.46 (0.21, 1.04)	16	0.86 (0.50, 1.49)	*	1.79 (0.48, 6.62)	5	2.05 (0.75, 5.56)
	Day 0: first dose/positive test	*	n/a	*	n/a	*	n/a	*	21.12 (4.86, 91.77)
	1–28 days: first dose/positive test	7	0.51 (0.23, 1.16)	16	0.89 (0.51, 1.54)	*	2.49 (0.67, 9.32)	*	0.99 (0.29, 3.33)
	−28 to −1 days: second dose	6	0.58 (0.24, 1.39)	7	0.90 (0.40, 2.01)	*	n/a		
	Day 0: second dose	*	n/a	*	n/a	*	n/a		
	1–28 days: second dose	17	1.79 (0.97, 3.28)	10	1.26 (0.62, 2.54)	*	n/a		
**Cardiac arrhythmia**	Baseline	5520	1.00	11,531	1.00	924	1.00	2,290	1.00
	−28 to −1 days: first dose/positive test	1036	0.86 (0.80, 0.92)	1,787	0.90 (0.85, 0.95)	147	1.00 (0.84, 1.19)	283	1.22 (1.08, 1.39)
	Day 0: first dose/positive test	17	0.39 (0.24, 0.62)	25	0.36 (0.25, 0.54)	6	1.25 (0.56, 2.79)	189	21.35 (18.37, 24.80)
	1–28 days: first dose/positive test	1172	0.94 (0.88, 1.01)	1,639	0.90 (0.85, 0.95)	108	0.90 (0.73, 1.10)	868	3.38 (3.12, 3.66)
	−28 to −1 days: second dose	839	0.77 (0.71, 0.83)	659	0.75 (0.69, 0.82)	18	0.67 (0.41, 1.08)		
	Day 0: second dose	20	0.51 (0.33, 0.80)	15	0.46 (0.27, 0.76)	*	n/a		
	1–28 days: second dose	990	0.97 (0.90, 1.04)	802	1.04 (0.96, 1.12)	18	1.46 (0.90, 2.38)		
**Age** > **= 40**									
**Myocarditis**	Baseline	455	1.00	278	1.00	6	1.00	82	1.00
	−28 to −1 days: first dose/positive test	73	0.76 (0.58, 1.00)	34	0.68 (0.47, 1.00)	*	n/a	25	3.22 (2.02, 5.15)
	Day 0: first dose/positive test	*	0.66 (0.16, 2.67)	*	n/a	*	n/a	31	94.81 (61.39, 146.42)
	1–28 days: first dose/positive test	121	1.33 (1.06, 1.67)	61	1.12 (0.83, 1.52)	*	n/a	117	12.18 (9.01, 16.46)
	−28 to −1 days: second dose	53	0.70 (0.51, 0.95)	41	0.93 (0.65, 1.32)	*	n/a		
	Day 0: second dose	*	0.73 (0.18, 2.95)	*	n/a	*	n/a		
	1–28 days: second dose	63	0.87 (0.65, 1.15)	46	0.96 (0.69, 1.34)	*	n/a		
**Pericarditis**	Baseline	524	1.00	307	1.00	*	1.00	69	1.00
	−28 to −1 days: first dose/positive test	57	0.59 (0.44, 0.78)	26	0.60 (0.39, 0.90)	*	n/a	24	4.30 (2.63, 7.03)
	Day 0: first dose/positive test	*	n/a	*	n/a	*	n/a	9	42.16 (20.65, 86.07)
	1–28 days: first dose/positive test	95	0.79 (0.62, 1.00)	43	0.80 (0.57, 1.12)	*	n/a	24	3.72 (2.30, 6.00)
	−28 to −1 days: second dose	52	0.40 (0.30, 0.55)	28	0.41 (0.28, 0.61)	*	n/a		
	Day 0: second dose	*	n/a	*	1.07 (0.34, 3.35)	*	n/a		
	1–28 days: second dose	100	0.82 (0.65, 1.04)	65	0.88 (0.66, 1.16)	*	n/a		
**Cardiac arrhythmia**	Baseline	112,836	1.00	102,437	1.00	297	1.00	12,829	1.00
	−28 to −1 days: first dose/positive test	16,526	0.75 (0.74, 0.76)	10,980	0.68 (0.67, 0.70)	37	0.64 (0.46, 0.91)	6,335	5.35 (5.18, 5.52)
	Day 0: first dose/positive test	272	0.33 (0.29, 0.37)	207	0.32 (0.28, 0.37)	*	1.50 (0.48, 4.69)	3,658	76.38 (73.54, 79.34)
	1–28 days: first dose/positive test	23,053	0.94 (0.93, 0.96)	16,720	0.88 (0.87, 0.90)	48	0.91 (0.67, 1.24)	8,072	5.71 (5.54, 5.87)
	−28 to −1 days: second dose	17,756	0.76 (0.74, 0.77)	14,351	0.75 (0.74, 0.77)	20	0.62 (0.39, 0.98)		
	Day 0: second dose	245	0.29 (0.25, 0.32)	242	0.32 (0.28, 0.36)	*	n/a		
	1–28 days: second dose	22,029	0.95 (0.94, 0.97)	20,145	0.94 (0.93, 0.96)	30	1.40 (0.95, 2.07)		
**Women**									
**Myocarditis**	Baseline	230	1.00	163	1.00	6	1.00	40	1.00
	−28 to −1 days: first dose/positive test	32	0.68 (0.45, 1.01)	15	0.51 (0.29, 0.87)	*	n/a	14	3.23 (1.71, 6.10)
	Day 0: first dose/positive test	*	1.39 (0.34, 5.63)	*	1.94 (0.48, 7.87)	*	n/a	13	70.71 (36.93, 135.37)
	1–28 days: first dose/positive test	58	1.40 (1.01, 1.93)	47	1.54 (1.08, 2.20)	*	n/a	53	11.00 (7.12, 16.99)
	−28 to −1 days: second dose	18	0.51 (0.31, 0.85)	17	0.86 (0.51, 1.47)	*	n/a		
	Day 0: second dose	*	n/a	*	n/a	*	n/a		
	1–28 days: second dose	23	0.63 (0.40, 0.99)	27	1.25 (0.81, 1.95)	*	n/a		
**Pericarditis**	Baseline	205	1.00	142	1.00	*	1.00	29	1.00
	−28 to −1 days: first dose/positive test	23	0.57 (0.36, 0.90)	14	0.61 (0.35, 1.08)	*	n/a	8	3.12 (1.37, 7.11)
	Day 0: first dose/positive test	*	n/a	*	n/a	*	n/a	5	50.44 (18.80, 135.30)
	1-28 days: first dose/positive test	27	0.57 (0.37, 0.88)	22	0.84 (0.53, 1.34)	*	n/a	12	4.29 (2.13, 8.63)
	−28 to −1 days: second dose	12	0.26 (0.14, 0.47)	11	0.40 (0.21, 0.75)				
	Day 0: second dose	*	n/a	*	1.82 (0.45, 7.41)				
	1–28 days: second dose	32	0.74 (0.50, 1.11)	23	0.80 (0.50, 1.27)				
**Cardiac arrhythmia**	Baseline	56,585	1.00	55,963	1.00	758	1.00	8,125	1.00
	−28 to −1 days: first dose/positive test	8,174	0.73 (0.71, 0.75)	6,097	0.68 (0.66, 0.70)	111	0.90 (0.73, 1.10)	3,286	4.37 (4.19, 4.56)
	Day 0: first dose/positive test	162	0.38 (0.33, 0.45)	117	0.33 (0.28, 0.40)	6	1.46 (0.65, 3.26)	1,725	56.62 (53.68, 59.73)
	1–28 days: first dose/positive test	11,651	0.93 (0.91, 0.95)	8,655	0.85 (0.83, 0.87)	78	0.75 (0.59, 0.95)	3,913	4.33 (4.17, 4.51)
	−28 to −1 days: second dose	8,811	0.75 (0.73, 0.77)	7,006	0.73 (0.71, 0.75)	22	0.71 (0.46, 1.11)		
	Day 0: second dose	118	0.28 (0.23, 0.33)	116	0.31 (0.25, 0.37)	*	n/a		
	1–28 days: second dose	10,872	0.95 (0.93, 0.97)	9,907	0.94 (0.92, 0.96)	29	1.77 (1.19, 2.61)		
**Men**									
**Myocarditis**	Baseline	318	1.00	234	1.00	16	1.00	79	1.00
	−28 to −1 days: first dose/positive test	49	0.73 (0.53, 1.01)	34	0.89 (0.62, 1.30)	*	0.59 (0.13, 2.74)	18	2.45 (1.44, 4.18)
	Day 0: first dose/positive test	*	n/a	*	n/a	*	n/a	23	77.15 (47.39, 125.59)
	1–28 days: first dose/positive test	83	1.21 (0.93, 1.59)	47	1.16 (0.84, 1.61)	8	3.79 (1.59, 9.04)	81	9.06 (6.51, 12.62)
	−28 to −1 days: second dose	40	0.78 (0.55, 1.11)	31	1.10 (0.74, 1.64)	*	3.61 (0.69, 18.95)		
	Day 0: second dose	*	n/a	0	n/a	*	n/a		
	1–28 days: second dose	60	1.26 (0.94, 1.71)	37	1.39 (0.96, 2.02)	*	12.27 (2.77, 54.37)		
**Pericarditis**	Baseline	375	1.00	272	1.00	10	1.00	66	1.00
	−28 to −1 days: first dose/positive test	41	0.53 (0.38, 0.74)	28	0.63 (0.42, 0.93)	*	1.94 (0.60, 6.26)	21	3.76 (2.24, 6.31)
	Day 0: first dose/positive test	*	n/a	*	n/a	*	n/a	6	27.82 (11.86, 65.26)
	1–28 days: first dose/positive test	74	0.82 (0.62, 1.07)	37	0.73 (0.51, 1.04)	*	1.96 (0.52, 7.34)	15	2.20 (1.24, 3.90)
	−28 to −1 days: second dose	46	0.51 (0.37, 0.71)	24	0.50 (0.33, 0.78)	*	n/a		
	Day 0: second dose	*	n/a	*	n/a	*	n/a		
	1–28 days: second dose	85	0.99 (0.77, 1.28)	52	1.01 (0.73, 1.38)	*	n/a		
**Cardiac arrhythmia**	Baseline	61,748	1.00	57,988	1.00	463	1.00	6,994	1.00
	−28 to −1 days: first dose/positive test	9,387	0.79 (0.77, 0.80)	6,670	0.73 (0.72, 0.75)	73	0.91 (0.71, 1.17)	3,332	5.09 (4.88, 5.32)
	Day 0: first dose/positive test	127	0.29 (0.24, 0.34)	115	0.32 (0.27, 0.39)	*	1.11 (0.36, 3.46)	2,122	81.29 (77.32, 85.47)
	1–28 days: first dose/positive test	12,568	0.95 (0.93, 0.97)	9,699	0.93 (0.91, 0.95)	78	1.13 (0.89, 1.45)	5,026	6.55 (6.31, 6.80)
	−28 to −1 days: second dose	9,784	0.76 (0.74, 0.78)	8,004	0.78 (0.76, 0.80)	16	0.60 (0.36, 1.01)		
	Day 0: second dose	147	0.31 (0.27, 0.37)	141	0.35 (0.29, 0.41)	*	n/a		
	1–28 days: second dose	12,143	0.95 (0.93, 0.97)	11,038	0.96 (0.94, 0.98)	19	1.18 (0.73, 1.89)		

n/a, not applicable.

In those aged under 40 years, we observed increased risks of myocarditis in the 1–28 days following a first dose of BNT162b2 (IRR 1.83, 95% CI 1.20, 2.79) and of mRNA-1273 (IRR 3.89, 95% CI 1.60, 9.44), after a second dose of BNT162b2 (IRR 3.40, 95% CI 1.91, 6.04) and of mRNA-1273 (IRR 20.71, 95% CI 4.02, 106.68) and following a SARS-CoV-2 positive test (IRR 4.06, 95%CI 2.21, 7.45). No association was found with the ChAdOx1 vaccine. In those aged 40 years or older, the risk of myocarditis was increased in the 1–28 days following a first dose of ChAdOx1 (IRR 1.33, 95% CI 1.06, 1.67) and a SARS-CoV-2 positive test (IRR 12.18, 95% CI 9.01, 16.46). No association was found with the BNT162b2 vaccine and numbers of events were insufficient to evaluate associations with the mRNA-1273 vaccine.

In women, we found an increased risk of myocarditis 1–28 days following a first dose of ChAdOx1 (IRR 1.40, 95% CI 1.01, 1.93) and BNT162b2 (IRR 1.54, 95% CI 1.08, 2.20), and following a SARS-CoV-2 positive test (IRR 11.00, 95% CI 7.12, 16.99). There were insufficient numbers of events to evaluate associations with the mRNA-1273 vaccine for women. In men, we found an increased risk of myocarditis at 1–28 days following a first and second dose of mRNA-1273 (IRR 3.79, 95% CI 1.59, 9.04 and IRR 12.27, 95%CI 2.77, 54.37, respectively) and following a SARS-CoV-2 positive test (IRR 9.06, 95% CI 6.51, 12.62). No association was found with the ChAdOx1 or BNT162b2 vaccines.

Supplementary Table 3b shows the IRRs of these outcomes estimated in the 1–28 days following exposure when restricting to different age groups (16–29, 29–39 and 40 plus years). The increased risk of myocarditis associated with either messenger RNA-based vaccine consistently occurs in the younger population (<40 years).

### Subgroup analyses by previous SARS-CoV-2 infection

Supplementary Table 4 shows the estimated IRRs for myocarditis, pericarditis or cardiac arrhythmias in the 1–28 day risk period after each vaccination in those who did not have a SARS-CoV-2 positive test before vaccination. These results generally agreed with the main analyses. We did not observe any increased risk of myocarditis, pericarditis or arrhythmia following a first or second dose of ChAdOx1 or BNT162b2 vaccine in those who tested positive before vaccination, but there was a decreased risk of cardiac arrythmias following a first dose of either vaccine (Supplementary Table 5). There were insufficient numbers of events to evaluate associations with the mRNA-1273 vaccine in this subgroup.

### Subgroup analyses by categories of cardiac arrhythmia

Cardiac arrhythmias (*n* = 385,508) were categorized as atrial fibrillation or flutter (*n* = 229,248, 59.4%), atrio-ventricular (AV) block and related conduction disorders (*n* = 114,701, 29.7%), ventricular tachycardia (*n* = 8,211, 2.1%), ventricular fibrillation (*n* = 2,910, 0.7%) and other, including supraventricular tachycardia (*n* = 130,485, 33.8%).

Over the 1–28 days postexposure, we observed an increased risk of atrial fibrillation or flutter arrhythmia at 15–21 days following a first dose of mRNA-1273 vaccine (IRR 2.06, 95% CI 1.11, 3.82); of ventricular fibrillation at 22–28 days following a second dose of ChAdOx1 vaccine (IRR 1.35, 95% CI 1.05, 1.74) and of other cardiac arrhythmia at 1–7 days following a second dose of mRNA-1273 vaccine (IRR 2.32, 95% CI 1.49, 3.62). There was an increased risk of all cardiac arrhythmia subgroups in the 1–28 days following a SARS-CoV-2 positive test (Supplementary Table 6).

### Robustness of results

We found no increased risk of celiac disease (negative control) across the prespecified time periods for the vaccine exposures, with the exception of the 15–21 days after the second dose of ChAdOx1 (IRR 1.20; 95% CI: 1.05, 1.36). We also found a decreased risk on the day of vaccination, as expected given the healthy vaccinee effect. Anaphylaxis (positive control) showed the expected increased risk on the day of vaccination (both first and second dose), but not at later periods following the ChAdOx1 and BNT162b2 vaccinations (Supplementary Table 7). There were insufficient numbers of events to evaluate associations between anaphylaxis and the mRNA-1273 vaccine. See Methods for clarification of the choice of controls outcomes.

**Extended Data Fig. 1:**
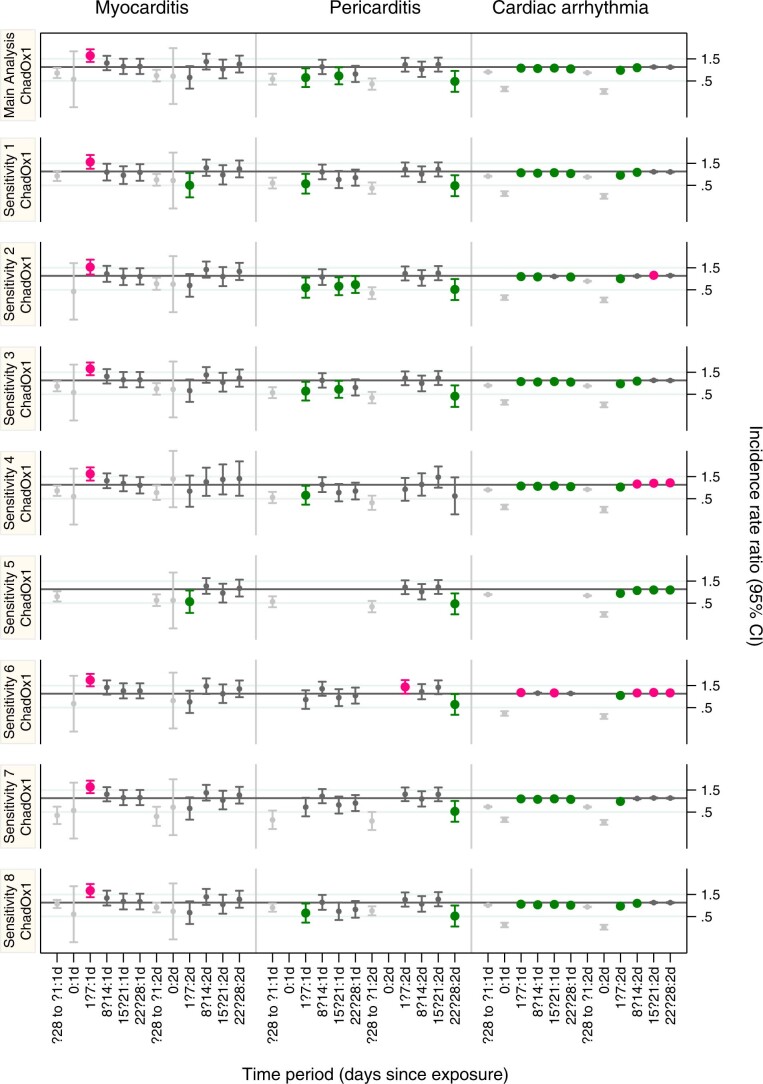
Incidence rate ratios (IRRs) with 95% confidence intervals associated with ChAdOx1 vaccine (sensitivity analyses). Incidence rate ratios (IRRs) with 95% confidence intervals for single outcomes in predefined risk periods immediately before and after 1^st^ and 2nd dose of ChAdOx1 vaccine computed using an underlying population of size n = 38,615,491 vaccinated individuals and different sensitivity analyses. Horizontal bold line indicates 1.

**Extended Data Fig. 2:**
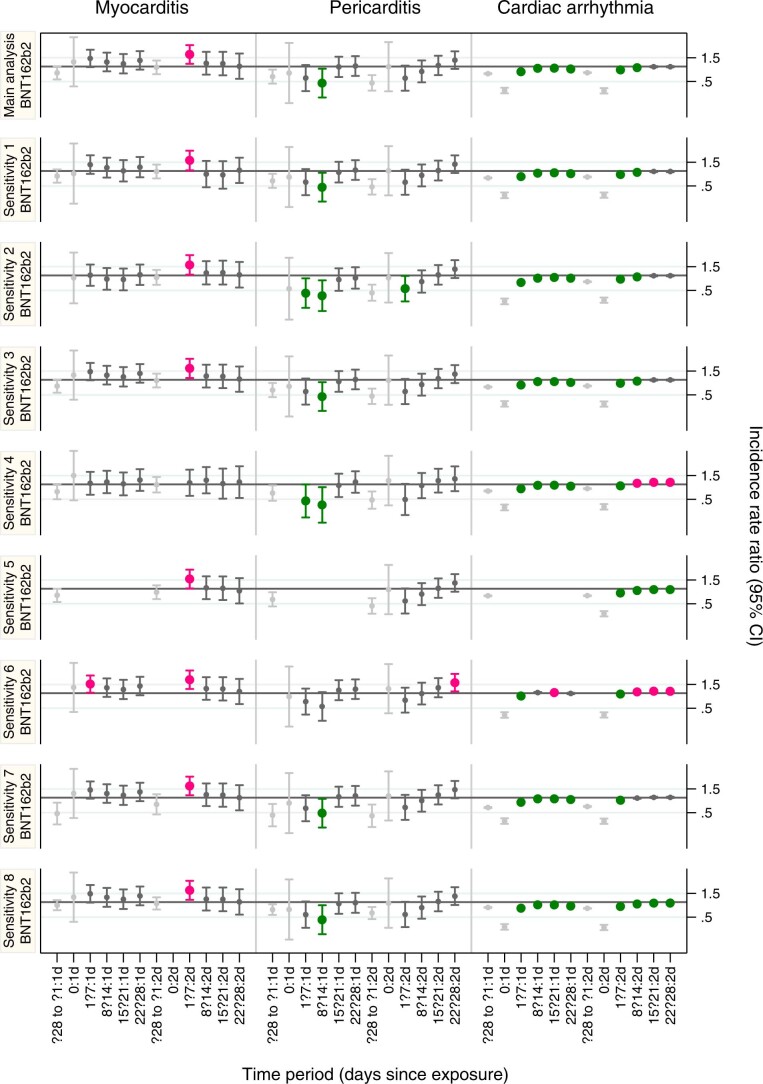
Incidence rate ratios (IRRs) with 95% confidence intervals associated with BNT162b2 vaccine (sensitivity analyses). Incidence rate ratios (IRRs) with 95% confidence intervals for single outcomes in predefined risk periods immediately before and after 1st and 2nd dose of BNT162b2 vaccine computed using an underlying population of size n = 38,615,491 vaccinated individuals and different sensitivity analyses. Horizontal bold line indicates 1.

**Extended Data Fig. 3:**
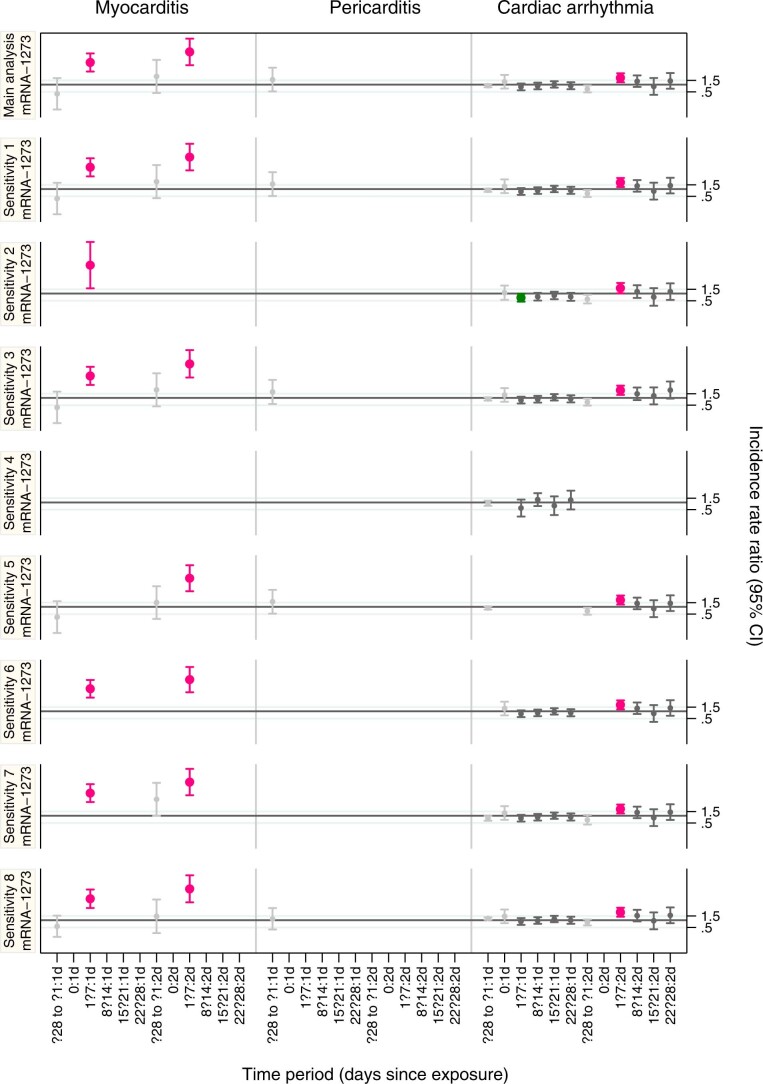
Incidence rate ratios (IRRs) with 95% confidence intervals associated with mRNA-1273 vaccine (sensitivity analyses). Incidence rate ratios (IRRs) with 95% confidence intervals for single outcomes in predefined risk periods immediately before and after 1st and 2nd dose of mRNA-1273 vaccine computed using an underlying population of size n = 38,615,491 vaccinated individuals and different sensitivity analyses. Horizontal bold line indicates 1.

Supplementary Table 8a and Extended Data Figs. 1–3 show the results for several sensitivity analyses. Overall, our main findings were not sensitive to censoring due to death, and IRRs for the second dose of vaccination agree with main results when we removed those who had the outcome after the first dose of any vaccine, but before the second.

As expected, sensitivity analyses 6–8 show that, by reducing the length of the prerisk period, we could exaggerate the relative incidence associated with vaccine exposure and understate the relative incidence associated with infection exposure, whereas increasing the length of the prerisk period has the opposite effect (Supplementary Table 8b).

A sensitivity analysis restricting the study period up to 17 May 2021, when the Centres for Disease Control and Prevention (CDC) announced cases of myocarditis after the BNT162b2 vaccine, showed no increased incidence of myocarditis in the 1–7 days following a second dose of BNT162b2 (IRR 1.07, 95% CI 0.59, 1.97). The age distribution of those vaccinated with ChAdOx1 in these two time periods was similar, but those vaccinated with BNT162b2 were older in the restricted study period (13.8% versus 29.7% were younger than 40 years; Supplementary Table 9).

### Hospital duration of stay for myocarditis

The median hospital duration of stay for those with myocarditis in the 28 days postvaccination was 3 days (interquartile range (IQR): 1, 9) for ChAdOx1, 3 days (IQR: 1, 7) for BNT162b2 and 4 days (IQR: 3, 6) for mRNA-1273, with means of 8.3, 5.7 and 4.5 days, respectively. This compares with a median of 4 days (IQR: 1, 9) and mean of 7.6 days for those whose admissions had not occurred in the 28 days post vaccination.

### Absolute measures of effect of vaccination and SARS-CoV-2 infection

**Fig. 2:**
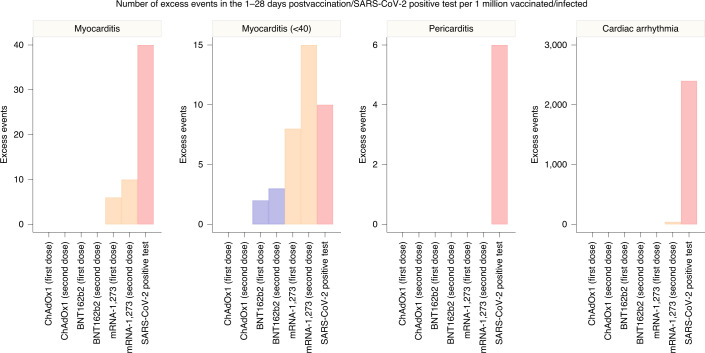
Number of excess events due to exposure per 1 million exposed, as reported in Supplementary Table 10. When IRR did not show a significant increase of incidence over the 1–28 days postvaccination or a SARS-CoV-2 positive test, absolute measures are not given.

We estimated the number of exposures needed for one excess event and the excess number of events per 1 million exposed for each outcome (Fig. 2 and Supplementary Table 10). In the 1–28 days following the first dose of the ChAdOx1, BNT162b2 and mRNA-1273 vaccine, an extra two (95% CI 0, 3), one (95%CI 0, 2) and six (95% CI 2, 8) myocarditis events per 1 million exposed would be anticipated, respectively. In the 1–28 days following the second dose of mRNA-1273, an extra ten (95% CI 7, 11) myocarditis events per 1 million persons would be anticipated. This compares with an extra 40 (95% CI 38, 41) myocarditis events per 1 million in the 1–28 days following a SARS-CoV-2 positive test.

Subgroup analyses by age showed that the increased risk of events associated with the two mRNA vaccines was present only in those aged under 40 years. For this age group, we estimated 2 (95% CI 1, 3) and 8 (95%CI 4, 9) excess cases of myocarditis per 1 million people receiving a first dose of BNT162b2 and mRNA-1273, respectively, and 3 (95% CI 2, 4) and 15 (95%CI 12, 16) excess cases of myocarditis per 1 million people receiving a second dose of BNT162b2 and mRNA-1273, respectively. This compares with ten (95% CI 7, 11) extra cases of myocarditis following a SARS-CoV-2 positive test in those aged under 40 years.

## Discussion

This is the largest study to date of acute cardiac outcomes after SARS-CoV-2 vaccination or infection, the first to compare the risk of cardiac events between different vaccine products and SARS-CoV2 infection and the first to investigate the association between cardiac events and the ChAdOx1 vaccine.

Our findings are relevant to the public, clinicians and policy makers. First, there was an increase in the risk of myocarditis within a week of receiving the first dose of both adenovirus and mRNA vaccines, and a higher increased risk after the second dose of both mRNA vaccines. In contrast, we found no evidence of an increase in the risk of pericarditis or cardiac arrhythmias following vaccination, except in the 1–28 days following a second dose of the mRNA-1273 vaccine. Second, in the same population, there was a greater risk of myocarditis, pericarditis and cardiac arrhythmia following SARS-CoV-2 infection. Third, the increased risk of myocarditis after vaccination was higher in persons aged under 40 years. We estimated extra myocarditis events to be between 1 and 10 per million persons in the month following vaccination, which was substantially lower than the 40 extra events per million persons observed following SARS-CoV-2 infection.

We assessed the temporal association between COVID-19 vaccination and cardiac adverse events using hospital admissions with diagnoses of myocarditis or pericarditis, and cardiac arrhythmias. Myocarditis is an inflammatory disorder of the myocardium that commonly results from viral infection, systemic immune-mediated diseases or immunomodulatory treatments^7^. It occurs more commonly in men, which may be a consequence of different effects of sex hormones on the immune system^8^. Several cases have been reported in patients hospitalized with SARS-CoV-2 infection, and screening for cardiac involvement using cardiac troponin testing has demonstrated that myocardial injury is common and associated with poor outcomes^9^. Irrespective of the underlying etiology of myocarditis, those who develop important ventricular impairment are at increased risk of cardiogenic shock and mortality^10^, highlighting the importance of ascertaining whether myocarditis may be temporally associated with vaccination for SARS-CoV-2.

Whereas myocarditis is a specific form of cardiac inflammation, pericarditis reflects inflammation localized to the pericardium, and the occurrence of cardiac arrhythmias, although associated with both, is not a specific indictor of cardiac inflammation. Thus, neither pericarditis nor any category of cardiac arrhythmia were associated specifically with COVID-19 vaccination^10–12^. Myocarditis is underdiagnosed in practice^13^, with clinical bias being directed towards myocardial ischemia or infarction. Thus, our use of diagnostic codes for myocarditis from routine data suggest that the ascertainment of cardiac inflammation after COVID-19 vaccination is likely to be under- rather than over-represented^14,15^.

Although no cases of myocarditis were observed in the randomized trials of vaccine, this condition is uncommon, and postmarket authorization surveillance may be required. Our observation of an increased risk within 7 days of receiving the vaccine is consistent with the presentation of viral myocarditis, where viral symptoms are often reported in the week leading up to presentation. Furthermore, myocarditis following vaccination has been reported with other vaccines, for example, in healthy adults after receipt of live vaccinia virus vaccines^16,17^ Whilst the mechanisms of myocarditis following exposure to SARS-CoV2 infection and vaccination are not known, it seems likely that systemic complications of infection are a consequence of an immune-mediated, virus-independent immunopathologic process^18^. However, vaccine mediated expression of SARS-CoV-2 surface spike protein on the surface of cardiomyocytes could potentially trigger an immunologic response resulting in organ-specific cell death^19,20^

Our findings are consistent with those from a case-control study of 884,828 persons receiving the BNT162b2 vaccine in Israel^21^. That study observed an association with myocarditis in the 42 days following vaccination (risk ratio of 3.24), but no association with pericarditis or cardiac arrhythmia. Two further studies from Israel add to our observations by providing clinical review to ensure robust case ascertainment^22^ and reporting investigations and outcomes in individual patients with myocarditis following the BNT162b2 vaccine^23^. Witberg et al.^21,22^ observed a small excess in events 3–5 days following the second dose of BNT162b2 vaccine, but most were mild presentations and just one classified as fulminant^22^. Mevorach et al. observed an incidence ratio of 5.34 for myocarditis in 5,442,696 persons following BNT162b2, although this was attenuated when restricted to the 136 definite and probable cases of myocarditis^23^. Risk of myocarditis was restricted to males under the age of 40 years and only observed following the second dose. Similarly, two studies from the United States have reported an incident rate ratio of 2.7 for myocarditis in the 10 days following the second dose of both mRNA vaccines^24^ and an estimated 6.3 and 10.1 extra cases per million doses in the 1- to 21-day period following the first and second dose of both mRNA vaccines, respectively, in those younger than 40 years^25^.

Our findings extend these observations by including 38 million adults in England receiving both mRNA and adenovirus-mediated vaccine. There were 1,615 myocarditis events in our study population, enabling a granular evaluation of subgroups and the temporal association in the weeks following vaccination. We observed a small excess in myocarditis events after both the first and second dose of vaccine, but this risk was restricted to a 7-day period following vaccination. This observation was not limited to the mRNA vaccines as we also found an excess in myocarditis events following the first dose of ChAdOx1 vaccine. The excess risk was observed in men and women but was only consistently observed following both mRNA vaccines in those younger than 40 years, although this may, in part, reflect the small number of individuals over the age of 40 years receiving the mRNA-1273 vaccine in England.

Whilst myocarditis can be life-threatening, most vaccine-associated myocarditis events have been mild and self-limiting^22^. The risk observed here is small and confined to the 7-day period following vaccination, whereas the lifetime risk of morbidity and mortality following SARS-CoV-2 infection is substantial. Indeed, myocardial injury is very common in persons admitted to hospital with SARS-CoV-2 infection^26^, when evaluated systematically using high-sensitivity cardiac troponin tests^27^. Moreover, evidence of myocardial injury, irrespective of whether due to myocarditis or myocardial ischemia, is associated with a higher risk of in-hospital death^28^. We estimate that the absolute number of excess myocarditis events in the 28 days following a first dose of adenovirus or mRNA vaccine is between one and six per million persons vaccinated, and the excess risk following the second dose of the mRNA-1283 vaccine is ten per million. By contrast, we estimate 40 excess myocarditis events per million in the 28 days following SARS-CoV-2 infection. The risks are more evenly balanced in younger persons aged up to 40 years, where we estimated the excess in myocarditis events following SARS-CoV-2 infection to be 10 per million with the excess following a second dose of mRNA-1273 vaccine being 15 per million. Further research is required to understand why the risk of myocarditis seems to be higher following mRNA-1273 vaccine. Although the wider societal benefits of controlling the spread of virus to those who are more vulnerable are substantial, these data may help inform public health policy and the choice of vaccine offered to younger adults.

This study has several strengths. First, the United Kingdom offered an ideal place to carry out this study given that three vaccinations have been rolled out at speed and scale. Second, this was a population-based study of data recorded prospectively and avoided recall and selection biases linked to case reports. Third, the large sample size provided sufficient power to investigate these rare outcomes, which could not be assessed through clinical trials. Fourth, the SCCS study design removes potential confounding from fixed characteristics, and the breakdown of our study period into weekly blocks accounted for temporal confounding. Of note, the estimated IRRs are consistently less than 1 in the pre-exposure period before vaccination and greater than 1 in the prerisk period before a SARS-CoV-2 positive test. This was expected since events are unlikely to happen shortly before vaccination (relatively healthy people are receiving the vaccine) and more likely to happen before a SARS-CoV-2 positive test (as a standard procedure, patients admitted to hospital are tested for SARS-CoV-2). We also assessed the robustness of our results through analyses of control outcomes and several sensitivity analyses.

There are some limitations that we should acknowledge. First, although we used an established methodology for evaluating vaccine safety, we cannot determine whether our findings are causal. Second, we relied on hospital admission codes and death certification to define our outcome measures. As such, we are not able to determine what proportion of patients underwent cardiac imaging or biopsy to confirm the diagnosis of myocarditis. It remains possible that our findings have been influenced by referral bias, with troponin testing performed more widely following vaccination due to media reports of vaccine-associated myocarditis. Our sensitivity analysis restricted to those persons vaccinated before the CDC announcement does not discount this possibility, although the different results could also be explained by the fact that the population who received the BNT162b2 vaccine were older in the restricted study period. Third, the mRNA-1273 vaccine roll-out began in April 2021 in the United Kingdom; as a consequence, the number of events in patients who received this vaccine was low. Although the signal associated with myocarditis is strong for this vaccine, care is needed in the interpretation, and it would be useful to replicate our results in similarly large datasets internationally. Fourth, we are unclear about the biological plausibility of the observed reduced risks of pericarditis and arrhythmia linked to vaccination and, although these findings are consistent of those of Barda et al.^21^, they should be interpreted with caution. Fifth, in this study, we performed several comparisons, which may lead to some erroneous inferences. As a consequence, careful interpretation is needed, especially for the borderline associations found. Finally, it is also important to note that control outcomes were chosen to assess the validity of the association between cardiac adverse events and vaccination. Control outcomes for a SARS-CoV-2 positive test are more challenging to find, as the entire health system is affected by the pandemic. Caution is needed in interpretation of the findings for a SARS-CoV-2 positive test in light of this.

In summary, this population-based study quantifies for the first time the risk of several rare cardiac adverse events associated with three COVID-19 vaccines as well as SARS-CoV-2 infection. Vaccination for SARS-CoV-2 in adults was associated with a small increase in the risk of myocarditis within a week of receiving the first dose of both adenovirus and mRNA vaccines, and after the second dose of both mRNA vaccines. By contrast, SARS-CoV-2 infection was associated with a substantial increase in the risk of hospitalization or death from myocarditis, pericarditis and cardiac arrhythmia.

## Methods

### Ethical approval

National Health Service Research Ethics Committee approval was obtained from East Midlands-Derby Research Ethics Committee [reference 04/03/2021].

### Data

We used the National Immunisation Database (NIMS) database of COVID-19 vaccination to identify vaccine exposure. This includes vaccine type, date and doses for all people vaccinated in England. We linked NIMS vaccination data, at individual level, to national data for mortality (Office for National Statistics), hospital admissions (Hospital Episode Statistics) and SARS-CoV-2 infection data (Second Generation Surveillance System).

### Study design

The SCCS design was used; this design was developed originally to examine vaccine safety^5,6^. The analyses are conditional on each case, so any fixed characteristics during the study period, such as sex, ethnicity or chronic conditions, are inherently controlled for. Any time-varying factors, like seasonal variation, need to be adjusted for in the analyses.

### Study period and population

We examined the associations between ChAdOx1, BNT162b2 or mRNA-1273 vaccines and selected cardiac conditions during the ongoing COVID-19 vaccination programme in England, which commenced on 8 December 2020. Separate analyses were carried out in cases with each cardiac outcome of interest. People were considered eligible for inclusion in each study cohort if they had received at least one vaccine dose, were at least 16 years old and were admitted to hospital with, or died from, the outcome of interest between 1 December 2020 and 24 August 2021 (last data update). Patients were followed up from the study start (1 December 2020) to the earliest of the end of the study period or when they died. Patients with a hospital admission for the same outcome in the 2 years before the start of the study period were excluded.

### Outcomes

**Extended Data Fig. 4:**
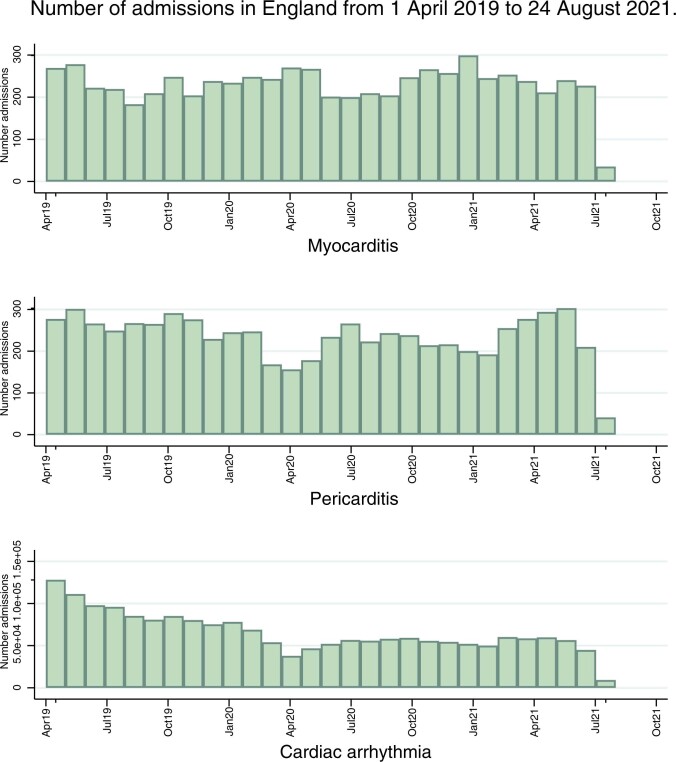
Number of admissions to hospital for single outcomes by month between April 2019 and August 2021. Number of admissions to hospital for single outcomes by month between April 2019 and August 2021.

The outcomes in this study are selected cardiac conditions with previous indications of association with SARS-CoV-2 infection or COVID-19 vaccination. These included myocarditis, pericarditis and arrythmia. We used the International Classification of Diseases-10 codes to define each outcome, as listed in Supplementary Table 11. The outcomes were identified as the first hospital admission due to the event of interest, or death recorded on the death certificate with the International Classification of Diseases-10 code related to the outcome of interest within the study period. A histogram showing the number of admissions in England from 1 April 2019 to 24 August 2021 is presented in Extended Data Fig. 4.

### Exposures

**Extended Data Fig. 5:**
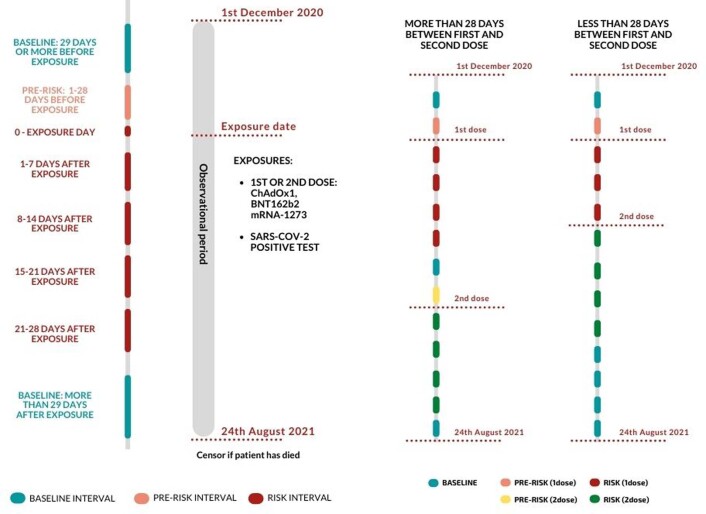
Schematic presentation of the SCCS study design. Extended Data Fig. 5: Schematic presentation of the SCCS study design.

The exposure variables were a first or second dose of the ChAdOx1, BNT162b2 or mRNA-1273 vaccines, or SARS-CoV-2 infection, defined as the first SARS-CoV-2 positive test in the study period. All exposures were included in the same model. We defined the exposure risk intervals as the following prespecified time periods: 0, 1–7, 8–14, 15–21 and 22–28 days after each exposure date, under the assumption that the adverse events under consideration are unlikely to be related to exposure later than 28 days postexposure. We assumed that the risks may be different after each vaccine dose (first and second), and hence we allowed for a dose effect, by defining a separate risk interval after each dose: 0, 1–7, 8–14, 15–21 or 22–28 days after the first dose and 0, 1–7, 8–14, 15–21 or 22–28 days after the second dose. To avoid overlapping risk periods, we assumed that later exposures (second dose) take precedence over earlier ones (first dose), except for the 28-day prerisk period for the second dose, as shown in Extended Data Fig. 5. A prerisk interval of 1–28 days before each exposure date was included to account for potential bias that might arise if the occurrence of the outcome temporarily influenced the likelihood of exposure. The baseline period for the vaccination exposures comprised the remaining time from 1 December 2020 until 29 days before the first dose date and from 29 days after the first dose until 29 days before the second dose (if applicable), and from 29 days after the second dose until 24 August 2021 or the censored date if earlier. A SARS-CoV-2 positive test was considered as a separate exposure in the models, which allowed overlapping risk windows with vaccination exposure.

### Seasonality and COVID-19 pandemic period

Hospital admissions were likely influenced by the pressure on the health systems due to COVID-19, which was not uniform during the pandemic period. To allow for these underlying seasonal effects, we split the study observation period into weeks and adjusted for week as a factor variable in the statistical models.

### Statistical analysis

We described characteristics of each cohort (vaccinated patients with the outcomes of interest) in terms of age, sex and ethnic group. The SCCS models were fitted using a conditional Poisson regression model with an offset for the length of the exposure risk period. Separate analyses were carried out for each cardiac outcome of interest. IRR, the relative rate of hospital admissions or deaths due to each outcome of interest in exposure risk periods relative to baseline periods, and their 95% CI were estimated by the SCCS model adjusted for week as a time-varying covariate. Exposure terms for vaccines and for a SARS-CoV-2 positive test were included in the same model.

We investigated if the associations between vaccine exposures and outcomes are sex- or age-dependent by running subgroups analyses amongst those aged under 40 years and those aged 40 years and older and by gender. We also conducted analyses restricted to those with a SARS-CoV-2 infection before vaccination and those without SARS-CoV-2 infection. Finally, we performed subgroup analyses by prespecified categories of cardiac arrythmia as reported in Supplementary Table 11.

We conducted sensitivity analyses to assess the robustness of results to assumptions, such as that the occurrence of an outcome event did not influence the probability of subsequent exposures by (1) excluding those who died from the outcome and (2) restricting analysis to the period postvaccination, without censoring at death. To assess potential reporting delays in the data by (3) restricting the study to the period up to 1 August 2021. To include only time unaffected by any notoriety bias by (4) restricting the study to the period up to 17 May 2021, when CDC announced cases of myocarditis after BNT162b2 vaccine and (5) removing patients who had outcomes in the 28 days after a first dose, but before a second dose, since they are less likely to have a second dose if they experienced an adverse event after the first.

To compare the choice of different pre-exposure risk periods, we also included three extra sensitivity analyses using different lengths for the prerisk period: (6) including the prerisk period in the baseline, (7) including only the 1–14 days before exposure in the prerisk period and (8) including a longer prerisk period of 59 days.

Stata v.17 was used for these analyses.

### Absolute risk

Absolute risk differences cannot be obtained using SCCS. We supplemented our estimates of IRRs with measures of effect using a method^29^ developed to estimate the number of exposures needed to produce one excess adverse outcome and the excess number of events per 1 million exposed for each outcome.

### SCCS assumptions

#### Independence between outcome and exposure

**Extended Data Fig. 6:**
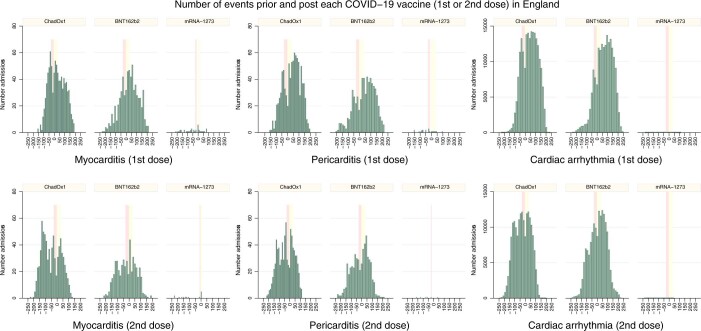
Number of hospital admissions or deaths for each outcome prior and post each vaccine dose by vaccine type. Pink area shows the 28 prior to vaccination and yellow area shows the 28 days postvaccination.

We assumed that patients who experienced an outcome before vaccination were likely to delay vaccination until symptoms had improved. Therefore, we included a prerisk period in the analyses, lasting from 1 to 28 days before vaccination, which removes this period from the baseline period (Extended Data Fig. 6). Hospital admissions for the events of interest can trigger COVID-19 testing. Such events may well be caused by SARS-CoV-2 infection, but the reverse causality involved in their detection induces bias. To reduce the bias, which could over or underestimate the effect of infection, we decided to allocate day 0 to a risk period on its own^30^.

#### Event-dependent observation periods

We tested this assumption with sensitivity analyses 1 and 2. These further analyses agreed with the main analysis, suggesting that there should be little concern for these outcomes.

### Negative and positive control outcomes

We examined the association of exposures with celiac disease as a negative control outcome^31^, which is assumed not to be associated with exposure to vaccination or SARS-CoV-2 infection; and with anaphylaxis as a positive control outcome given that it could occur shortly after vaccination with either vaccine^32^.

### Reporting Summary

Further information on research design is available in the Nature Research Reporting Summary linked to this article.

## Online content

Any methods, additional references, Nature Research reporting summaries, source data, extended data, supplementary information, acknowledgements, peer review information; details of author contributions and competing interests; and statements of data and code availability are available at 10.1038/s41591-021-01630-0.

## Supplementary information

Supplementary Information Supplementary Tables 1–11.

Reporting Summary

## Data Availability

The data that support the findings of this study—NIMS Database of COVID-19, mortality (Office of National Statistics), hospital admissions (Hospital Episode Statistics) and SARS-CoV-2 infection data (PHE)—are not publicly available because they are based on deidentified national clinical records. Due to national and organizational data privacy regulations, individual-level data such as those used for this study cannot be shared openly.

## Code availability

The code used for this study has been deposited in the git repository of the research group, which is protected by privacy. Access to the code is available from the authors on request for noncommercial, academic and research use only.

## References

[1] Number of people vaccinated against COVID-19. *Our World in Data*; https://ourworldindata.org/explorers/coronavirus-data-explorer?zoomToSelection=true&time=2021-09-30&facet=none&pickerSort=asc&pickerMetric=location&Metric=People+vaccinated+%28by+dose%29&Interval=7-day+rolling+average&Relative+to+Population=false&Align+outbreaks=false&country=~ITA (2021).

[2] Selected adverse events reported after COVID-19 vaccination. *Centers for Disease Control and Prevention*; https://www.cdc.gov/coronavirus/2019-ncov/vaccines/safety/adverse-events.html (2021).

[3] Comirnaty and Spikevax: possible link to very rare cases of myocarditis and pericarditis. *European Medicines Agency*; https://www.ema.europa.eu/en/news/comirnaty-spikevax-possible-link-very-rare-cases-myocarditis-pericarditis (2021).

[4] Surveillance of myocarditis (inflammation of the heart muscle) cases between December 2020 and May 2021 (including). Ministry of Health of the Israeli Government; https://www.gov.il/en/departments/news/01062021-03 (2021).

[5] Petersen I, Douglas I, Whitaker H (2016). Self controlled case series methods: an alternative to standard epidemiological study designs. Brit. Med. J..

[6] Farrington CP, Nash J, Miller E (1996). Case series analysis of adverse reactions to vaccines: a comparative evaluation. Am. J. Epidemiol..

[7] Pollack A (2015). Viral myocarditis–diagnosis, treatment options, and current controversies. Nat. Rev. Cardiol..

[8] Fairweather D, Cooper LT, Blauwet LA (2013). Sex and gender differences in myocarditis and dilated cardiomyopathy. Curr. Probl. Cardiol..

[9] Chapman AR, Bularga A, Mills NL (2020). High-sensitivity cardiac troponin can be an ally in the fight against COVID-19. Circulation.

[10] Kociol RD (2020). Recognition and initial management of fulminant myocarditis: a scientific statement from the American Heart Association. Circulation.

[11] Friedrich MG (2009). Cardiovascular magnetic resonance in myocarditis: a JACC white paper. J. Am. Coll. Cardiol..

[12] Ferreira VM (2018). Cardiovascular magnetic resonance in nonischemic myocardial inflammation: expert recommendations. J. Am. Coll. Cardiol..

[13] Pasupathy S (2015). Systematic review of patients presenting with suspected myocardial infarction and nonobstructive coronary arteries. Circulation.

[14] Dastidar AG (2019). Prognostic role of CMR and conventional risk factors in myocardial infarction with nonobstructed coronary arteries. JACC Cardiovasc. Imaging.

[15] Kim JY, Han K, Suh YJ (2021). Prevalence of abnormal cardiovascular magnetic resonance findings in recovered patients from COVID-19: a systematic review and meta-analysis. J. Cardiovasc. Magn. Reson..

[16] Mei R (2018). Myocarditis and pericarditis after immunization: gaining insights through the Vaccine Adverse Event Reporting System. Int. J. Cardiol..

[17] Engler RJ (2015). A prospective study of the incidence of myocarditis/pericarditis and new onset cardiac symptoms following smallpox and influenza vaccination. PLoS ONE.

[18] Dorward DA (2021). Tissue-specific immunopathology in fatal COVID-19. Am. J. Respir. Crit. Care Med..

[19] Bozkurt B, Kamat I, Hotez PJ (2021). Myocarditis with COVID-19 mRNA vaccines. Circulation.

[20] Makunts T (2021). Myocarditis occurrence with cancer immunotherapy across indications in clinical trial and post-marketing data. Sci. Rep..

[21] Barda N (2021). Safety of the BNT162b2 mRNA Covid-19 vaccine in a nationwide setting. N. Engl. J. Med..

[22] Witberg, G. et al. Myocarditis after Covid-19 vaccination in a large health care organization. *N. Engl. J. Med*. 10.1056/NEJMoa2110737 (2021). 10.1056/NEJMoa2110737 PMC8531986 34614329

[23] Mevorach, D. et al. Myocarditis after BNT162b2 mRNA vaccine against Covid-19 in Israel. *N. Engl. J. Med*. 10.1056/NEJMoa2109730 (2021). 10.1056/NEJMoa2109730 PMC8531987 34614328

[24] Simone, A. et al. Acute myocarditis following COVID-19 mRNA vaccination in adults aged 18 years or older. *JAMA Intern. Med.*10.1001/jamainternmed.2021.5511 (2021). 10.1001/jamainternmed.2021.5511 PMC8491129 34605853

[25] Klein NP (2021). Surveillance for adverse events after COVID-19 mRNA vaccination. JAMA.

[26] Sandoval Y, Januzzi JL, Jaffe AS (2020). Cardiac troponin for assessment of myocardial injury in COVID-19: JACC review topic of the week. J. Am. Coll. Cardiol..

[27] De Michieli L (2021). High-sensitivity cardiac troponin T for the detection of myocardial injury and risk stratification in COVID-19. Clin. Chem..

[28] Lala A (2020). Prevalence and impact of myocardial injury in patients hospitalized with COVID-19 infection. J. Am. Coll. Cardiol..

[29] Wilson K, Hawken S (2013). Drug safety studies and measures of effect using the self-controlled case series design. Pharmacoepidemiol. Drug Saf..

[30] Fonseca-Rodríguez O (2021). Avoiding bias in self-controlled case series studies of coronavirus disease 2019. Stat Med..

[31] Lipsitch (2010). Negative controls: a tool for detecting confounding and bias in observational studies. Epidemiology.

[32] Kounis NG (2021). Allergic reactions to current available COVID-19 vaccinations: pathophysiology, causality, and therapeutic considerations. Vaccines (Basel).

