# Supplementary material for: Risks of myocarditis, pericarditis, and cardiac arrhythmias associated with COVID-19 vaccination or SARS-CoV-2 infection
Source: Nat Med. 2021 Dec 14;28(2):410–22. doi: 10.1038/s41591-021-01630-0 (PMC8863574; doi:10.1038/s41591-021-01630-0)
Supplement: Supplementary file 1 — Supplementary Tables 1–11. [file 41591_2021_1630_MOESM1_ESM.pdf]

nature portfolio

https://doi.org/10.1038/s41591-021-01630-0

Supplementary information

Risks of myocarditis, pericarditis, and cardiac arrhythmias associated with COVID-19 vaccination or SARS-CoV-2 infection

In the format provided by the authors and unedited

Supplementary table 1: Demographic characteristics of patients who died for the individual outcomes in the 1-28 days following a first or second dose of COVID-19 vaccine or SARS-CoV-2 infection amongst the vaccinated population in England from December 1 2020 until August 24 2021 (cells with * are suppressed).

	**Deaths for myocarditis**							**Deaths for pericarditis**						
	**1-28 days post 1st dose**			**1-28 days post 2nd dose**			**1-28 days post test**	**1-28 days post 1st dose**			**1-28 days post 2nd dose**			**1-28 days post test**
	**ChAdOx1 nCoV-19 vaccine**	**BNT162b2 mRNA vaccine**	**mRNA-1273**	**ChAdOx1n CoV-19 vaccine**	**BNT162b 2 mRNA vaccine**	**mRNA-1273**	**Positive SARS-CoV-2 test**	**ChAdOx1n CoV-19 vaccine**	**BNT162b 2 mRNA vaccine**	**mRNA-1273**	**ChAdOx1n CoV-19 vaccine**	**BNT162b 2 mRNA vaccine**	**mRN A-1273**	**Positive SARS-CoV-2 test**
Total number of people	31	15	0	5	10	0	*	*	*	0	*	*	0	0
Women	38.7 (12)	60.0 (9)	-	*	50.0 (5)	-	*	*	*	-	*	*	-	-
Men	58.1 (18)	40.0 (6)	-	*	50.0 (5)	-	*	*	*	-	*	*	-	-
Not recorded	3.2 (1)	0	-	*	0	-	0	0	0	-	0	0	-	-
Mean age (SD)	64.4 (17.7)	72.2 (16.8)	-	70.6 (4.8)	74.5 (19.3)	-	82.3 (10.6)	71.5 (6.0)	86.0 (.)	-	74.3 (16.8)	81.0 (.)	-	-
Days between deaths and exposure (mean, SD)	12.7 (7.8)	14.1 (7.6)	-	9.6 (6.9)	11.3 (5.2)	-	12.0 (7.8)	8.3 (3.0)	21.0 (.)	-	10.7 (9.6)	16.0 (.)	-	-
	**Deaths for cardiac arrhythmia**													

	1-28 days post 1st dose			1-28 days post 2nd dose			1-28 days post test
	ChAdOx1 nCoV-19 vaccine	BNT162b2 mRNA vaccine	mRNA-1273	ChAdOx1n CoV-19 vaccine	BNT162b 2 mRNA vaccine	mRNA-1273	Positive SARS-CoV-2 test
Total number of people	904	755	0	478	524	0	131
Women	54.1 (489)	47.7 (360)	-	53.6 (256)	46.8 (245)	-	50.4 (66)
Men	45.4 (410)	51.7 (390)	-	45.6 (218)	52.9 (277)	-	48.9 (64)
Not recorded	0.6 (5)	0.7 (5)	-	0.8 (4)	0.4 (2)	-	0.8 (1)
Mean age (SD)	84.9 (9.6)	85.7 (7.4)	-	84.3 (9.8)	85.6 (7.6)	-	85.8 (6.8)
Days between deaths and exposure (mean, SD)	14.5 (8.2)	14.1 (7.7)	-	15.0 (8.0)	15.1 (8.0)	-	14.5 (6.8)

Supplementary table 2: Incidence rate ratios (IRR 95% CI) by sex for the outcomes in pre-defined risk periods immediately before and after exposure to vaccination and before and after a positive SARS-CoV-2 test result, adjusted for calendar time from December 1 2020 to August 24 2021 (cells with < 5 are suppressed).

		ChAdOx1 vaccine		BNT162b2 mRNA vaccine		mRNA-1273 vaccine		Positive SARS-CoV-2 test	
	**Time period**	**events**	**IRR (95% CI)**	**events**	**IRR (95% CI)**	**events**	**IRR (95% CI)**	**events**	**IRR (95% CI)**
**Women**									
**Myocarditis**	Baseline	230	1.00	163	1.00	6	1.00	40	1.00
	-28 to -1 days: 1st dose/positive test	32	0.72 (0.48, 1.07)	15	0.54 (0.31, 0.93)	*	n/a	14	3.40 (1.80, 6.45)
	0 day: 1st dose/positive test	*	1.41 (0.35, 5.72)	*	1.96 (0.48, 7.95)	*	n/a	13	74.94 (38.98, 144.09)
	1-7 days: 1st dose/positive test	19	1.86 (1.13, 3.06)	11	1.31 (0.69, 2.48)	*	n/a	28	23.06 (13.79, 38.55)
	8-14 days: 1st dose/positive test	11	1.02 (0.55, 1.91)	13	1.55 (0.85, 2.81)	*	n/a	17	14.06 (7.77, 25.46)
	15-21 days: 1st dose/positive test	14	1.26 (0.72, 2.22)	13	1.66 (0.92, 2.99)	*	n/a	7	6.09 (2.68, 13.82)
	22-28 days: 1st dose/positive test	14	1.30 (0.74, 2.28)	10	1.47 (0.76, 2.83)	*	n/a	*	n/a
	-28 to -1 days: 2nd dose	18	0.51 (0.31, 0.84)	17	0.84 (0.49, 1.43)	*	n/a		
	0 day: 2nd dose	*	n/a	*	n/a	*	n/a		
	1-7 days: 2nd dose	*	0.22 (0.05, 0.88)	8	1.45 (0.70, 3.00)	*	n/a		
	8-14 days: 2nd dose	6	0.65 (0.28, 1.48)	9	1.62 (0.81, 3.23)	*	n/a		
	15-21 days: 2nd dose	5	0.54 (0.22, 1.33)	6	1.11 (0.48, 2.55)	*	n/a		
	22-28 days: 2nd dose	10	1.08 (0.56, 2.06)	*	0.74 (0.27, 2.01)	*	n/a		
**Pericarditis**	Baseline	205	1.00	142	1.00	*	1.00	29	1.00

	-28 to -1 days: 1st dose/positive test	23	0.58 (0.37, 0.91)	14	0.61 (0.35, 1.08)	*	n/a	8	3.20 (1.40, 7.31)
	0 day: 1st dose/positive test	*	n/a	*	n/a	*	n/a	5	52.31 (19.40, 141.05)
	1-7 days: 1st dose/positive test	3	0.27 (0.09, 0.87)	6	0.97 (0.42, 2.23)	*	n/a	5	7.95 (2.96, 21.31)
	8-14 days: 1st dose/positive test	10	0.88 (0.46, 1.70)	*	0.60 (0.22, 1.65)	*	n/a	*	6.19 (2.11, 18.18)
	15-21 days: 1st dose/positive test	6	0.49 (0.22, 1.13)	*	0.46 (0.14, 1.45)	*	n/a	*	n/a
	22-28 days: 1st dose/positive test	8	0.63 (0.31, 1.30)	9	1.29 (0.65, 2.57)	*	n/a	*	2.41 (0.57, 10.22)
	-28 to -1 days: 2nd dose	12	0.26 (0.14, 0.47)	11	0.40 (0.21, 0.75)	*	n/a		
	0 day: 2nd dose	*	n/a	*	1.83 (0.45, 7.44)	*	n/a		
	1-7 days: 2nd dose	7	0.64 (0.30, 1.38)	5	0.67 (0.27, 1.65)	*	n/a		
	8-14 days: 2nd dose	9	0.81 (0.41, 1.61)	*	0.57 (0.21, 1.56)	*	n/a		
	15-21 days: 2nd dose	11	1.03 (0.55, 1.92)	9	1.26 (0.63, 2.52)	*	n/a		
	22-28 days: 2nd dose	5	0.50 (0.20, 1.22)	5	0.70 (0.28, 1.74)	*	n/a		
**Cardiac arrhythmia**	Baseline	56585	1.00	55963	1.00	758	1.00	8125	1.00
	-28 to -1 days: 1st dose/positive test	8174	0.75 (0.73, 0.77)	6097	0.69 (0.67, 0.71)	111	0.90 (0.74, 1.10)	3286	4.46 (4.27, 4.65)
	0 day: 1st dose/positive test	162	0.38 (0.33, 0.45)	117	0.33 (0.28, 0.40)	6	1.46 (0.65, 3.27)	1725	57.83 (54.81, 61.01)
	1-7 days: 1st dose/positive test	2844	0.94 (0.90, 0.98)	1878	0.75 (0.72, 0.79)	21	0.73 (0.47, 1.13)	1949	9.19 (8.73, 9.67)
	8-14 days: 1st dose/positive test	2870	0.91 (0.88, 0.95)	2258	0.87 (0.83, 0.91)	19	0.69 (0.44, 1.09)	1111	5.10 (4.78, 5.43)
	15-21 days: 1st dose/positive test	3028	0.95 (0.91, 0.99)	2317	0.88 (0.84, 0.91)	19	0.76 (0.48, 1.20)	472	2.07 (1.89, 2.27)

	22-28 days: 1st dose/positive test	2909	0.90 (0.87, 0.94)	2202	0.86 (0.83, 0.90)	19	0.83 (0.53, 1.31)	381	1.59 (1.43, 1.76)
	-28 to -1 days: 2nd dose	8811	0.75 (0.73, 0.77)	7006	0.73 (0.71, 0.75)	22	0.72 (0.46, 1.11)		
	0 day: 2nd dose	118	0.28 (0.23, 0.33)	116	0.30 (0.25, 0.37)	*	n/a		
	1-7 days: 2nd dose	2527	0.86 (0.82, 0.89)	2272	0.85 (0.82, 0.89)	14	2.37 (1.38, 4.07)		
	8-14 days: 2nd dose	2759	0.95 (0.91, 0.99)	2503	0.94 (0.90, 0.98)	7	1.49 (0.70, 3.16)		
	15-21 days: 2nd dose	2856	1.00 (0.97, 1.04)	2595	0.98 (0.94, 1.02)	3	0.86 (0.28, 2.70)		
	22-28 days: 2nd dose	2730	0.98 (0.95, 1.02)	2537	0.97 (0.93, 1.01)	5	2.17 (0.89, 5.29)		
**Men**									
**Myocarditis**	Baseline	318	1.00	234	1.00	16	1.00	79	1.00
	-28 to -1 days: 1st dose/positive test	49	0.76 (0.55, 1.05)	34	0.91 (0.63, 1.32)	*	0.58 (0.12, 2.69)	18	2.54 (1.49, 4.34)
	0 day: 1st dose/positive test	*	n/a	*	n/a	*	n/a	23	80.42 (49.25, 131.32)
	1-7 days: 1st dose/positive test	28	1.73 (1.15, 2.60)	16	1.59 (0.95, 2.67)	7	11.76 (4.75, 29.13)	40	19.94 (13.27, 29.97)
	8-14 days: 1st dose/positive test	24	1.35 (0.87, 2.09)	10	0.98 (0.52, 1.87)	*	n/a	20	9.50 (5.72, 15.80)
	15-21 days: 1st dose/positive test	16	0.89 (0.53, 1.50)	8	0.77 (0.38, 1.57)	*	n/a	11	4.94 (2.60, 9.40)
	22-28 days: 1st dose/positive test	15	0.82 (0.48, 1.40)	13	1.26 (0.71, 2.24)	*	n/a	10	4.15 (2.12, 8.12)
	-28 to -1 days: 2nd dose	40	0.77 (0.54, 1.09)	31	1.09 (0.73, 1.62)	*	3.47 (0.66, 18.17)		
	0 day: 2nd dose	*	n/a	*	n/a	*	n/a		
	1-7 days: 2nd dose	11	0.88 (0.47, 1.62)	15	2.06 (1.20, 3.54)	*	27.80 (6.39, 120.88)		
	8-14 days: 2nd dose	21	1.74 (1.10, 2.76)	6	0.87 (0.38, 1.99)	*	n/a		
	15-21 days: 2nd dose	14	1.20 (0.69, 2.08)	8	1.23 (0.60, 2.53)	*	n/a		
	22-28 days: 2nd dose	14	1.23 (0.71, 2.12)	8	1.27 (0.62, 2.62)	*	n/a		
**Pericarditis**	Baseline	375	1.00	272	1.00	10	1.00	66	1.00
	-28 to -1 days: 1st dose/positive test	41	0.53 (0.38, 0.75)	28	0.63 (0.42, 0.94)	*	1.93 (0.60, 6.23)	21	3.80 (2.26, 6.39)

	0 day: 1st dose/positive test	*	n/a	*	n/a	*	n/a	6	27.81 (11.84, 65.30)
	1-7 days: 1st dose/positive test	15	0.71 (0.42, 1.20)	5	0.40 (0.16, 0.97)	*	n/a	6	3.70 (1.58, 8.66)
	8-14 days: 1st dose/positive test	24	1.08 (0.71, 1.65)	5	0.39 (0.16, 0.95)	*	n/a	5	2.92 (1.16, 7.33)
	15-21 days: 1st dose/positive test	17	0.73 (0.44, 1.20)	16	1.27 (0.76, 2.12)	*	n/a	*	1.70 (0.53, 5.46)
	22-28 days: 1st dose/positive test	18	0.76 (0.47, 1.23)	11	0.87 (0.47, 1.60)	*	n/a	*	n/a
	-28 to -1 days: 2nd dose	46	0.51 (0.37, 0.71)	24	0.51 (0.33, 0.78)	*	n/a		
	0 day: 2nd dose	*	n/a	*	n/a	*	n/a		
	1-7 days: 2nd dose	30	1.36 (0.92, 2.00)	7	0.53 (0.25, 1.13)	*	n/a		
	8-14 days: 2nd dose	20	0.93 (0.58, 1.47)	12	0.92 (0.51, 1.66)	*	n/a		
	15-21 days: 2nd dose	25	1.18 (0.77, 1.79)	12	0.93 (0.52, 1.68)	*	n/a		
	22-28 days: 2nd dose	10	0.49 (0.26, 0.92)	21	1.70 (1.08, 2.69)	*	n/a		
**Cardiac arrhythmia**	Baseline	61748	1.00	57988	1.00	463	1.00	6994	1.00
	-28 to -1 days: 1st dose/positive test	9387	0.81 (0.79, 0.82)	6670	0.75 (0.73, 0.77)	73	0.91 (0.71, 1.17)	3332	5.24 (5.02, 5.48)
	0 day: 1st dose/positive test	127	0.29 (0.24, 0.34)	115	0.32 (0.27, 0.39)	*	1.12 (0.36, 3.48)	2122	83.82 (79.71, 88.15)
	1-7 days: 1st dose/positive test	3026	0.95 (0.92, 0.99)	2079	0.82 (0.78, 0.86)	17	0.93 (0.57, 1.51)	2644	14.70 (14.03, 15.40)
	8-14 days: 1st dose/positive test	3111	0.94 (0.91, 0.98)	2578	0.97 (0.93, 1.01)	21	1.19 (0.77, 1.85)	1531	8.28 (7.83, 8.76)
	15-21 days: 1st dose/positive test	3213	0.95 (0.91, 0.98)	2654	0.97 (0.94, 1.01)	24	1.43 (0.95, 2.16)	494	2.57 (2.34, 2.81)
	22-28 days: 1st dose/positive test	3218	0.93 (0.90, 0.97)	2388	0.91 (0.87, 0.95)	16	1.01 (0.61, 1.67)	357	1.76 (1.58, 1.96)
	-28 to -1 days: 2nd dose	9784	0.76 (0.74, 0.78)	8004	0.78 (0.76, 0.80)	16	0.61 (0.36, 1.01)		
	0 day: 2nd dose	147	0.31 (0.27, 0.37)	141	0.34 (0.29, 0.41)	*	n/a		
	1-7 days: 2nd dose	2708	0.83 (0.80, 0.86)	2464	0.86 (0.82, 0.89)	8	1.48 (0.73, 3.01)		

	8-14 days: 2nd dose	3172	0.98 (0.95, 1.02)	2785	0.96 (0.92, 1.00)	6	1.31 (0.58, 2.97)		
	15-21 days: 2nd dose	3161	1.00 (0.96, 1.04)	2899	1.00 (0.96, 1.04)	*	0.83 (0.27, 2.62)		
	22-28 days: 2nd dose	3102	1.00 (0.97, 1.04)	2890	1.01 (0.97, 1.05)	*	0.78 (0.19, 3.16)		

Supplementary table 3a: Incidence rate ratios (IRR 95% CI) by age groups (older or younger than 40) for the outcomes in pre-defined risk periods immediately before and after exposure to vaccination and before and after a positive SARS-CoV-2 test result, adjusted for calendar time from December 1 2020 to August 24 2021 (cells with < 5 are suppressed).

		ChAdOx1 vaccine		BNT162b2 mRNA vaccine		mRNA-1273 vaccine		Positive SARS-CoV-2 test	
	**Time period**	**events**	**IRR (95% CI)**	**events**	**IRR (95% CI)**	**events**	**IRR (95% CI)**	**events**	**IRR (95% CI)**
**Age <40**									
**Myocarditis**	Baseline	95	1.00	120	1.00	16	1.00	37	1.00
	-28 to -1 days: 1st dose/positive test	8	0.38 (0.18, 0.81)	15	0.96 (0.55, 1.68)	*	0.77 (0.17, 3.51)	7	1.75 (0.76, 4.05)
	0 day: 1st dose/positive test	*	n/a	*	3.30 (0.81, 13.50)	*	n/a	5	31.41 (11.91, 82.80)
	1-7 days: 1st dose/positive test	14	2.49 (1.36, 4.58)	14	3.16 (1.77, 5.66)	6	10.92 (4.09, 29.16)	10	9.06 (4.31, 19.02)
	8-14 days: 1st dose/positive test	*	0.34 (0.08, 1.41)	11	2.30 (1.20, 4.41)	*	n/a	*	n/a
	15-21 days: 1st dose/positive test	*	0.68 (0.24, 1.90)	5	1.11 (0.45, 2.77)	*	n/a	*	2.97 (0.90, 9.85)
	22-28 days: 1st dose/positive test	*	n/a	*	0.68 (0.21, 2.18)	*	n/a	*	2.67 (0.79, 8.99)
	-28 to -1 days: 2nd dose	5	0.39 (0.15, 0.99)	7	0.91 (0.41, 2.05)	*	4.62 (0.79, 26.97)		
	0 day: 2nd dose	*	n/a	*	n/a	*	n/a		

	1-7 days: 2nd dose	*	0.60 (0.14, 2.49)	14	8.15 (4.37, 15.20)	*	44.43 (8.92, 221.24)		
	8-14 days: 2nd dose	11	3.24 (1.65, 6.39)	*	1.36 (0.32, 5.67)	*	n/a		
	15-21 days: 2nd dose	*	0.57 (0.14, 2.35)	*	1.59 (0.38, 6.64)	*	n/a		
	22-28 days: 2nd dose	6	1.63 (0.69, 3.85)	*	n/a	*	n/a		
**Pericarditis**	Baseline	57	1.00	107	1.00	11	1.00	26	1.00
	-28 to -1 days: 1st dose/positive test	7	0.46 (0.21, 1.04)	16	0.86 (0.50, 1.49)	*	1.77 (0.48, 6.57)	5	2.08 (0.76, 5.66)
	0 day: 1st dose/positive test	*	n/a	*	n/a	*	n/a	*	21.59 (4.96, 94.00)
	1-7 days: 1st dose/positive test	*	0.58 (0.14, 2.44)	*	0.81 (0.29, 2.23)	*	n/a	*	2.67 (0.62, 11.49)
	8-14 days: 1st dose/positive test	*	0.58 (0.14, 2.44)	*	0.43 (0.10, 1.76)	*	n/a	*	n/a
	15-21 days: 1st dose/positive test	*	n/a	6	1.38 (0.60, 3.21)	*	n/a	*	n/a
	22-28 days: 1st dose/positive test	*	0.59 (0.14, 2.47)	*	0.98 (0.35, 2.70)	*	n/a	*	n/a
	-28 to -1 days: 2nd dose	6	0.58 (0.24, 1.39)	7	0.90 (0.40, 2.01)	*	n/a		
	0 day: 2nd dose	*	n/a	*	n/a	*	n/a		
	1-7 days: 2nd dose	6	2.25 (0.93, 5.48)	*	1.30 (0.40, 4.23)	*	n/a		
	8-14 days: 2nd dose	5	2.00 (0.77, 5.23)	*	1.43 (0.44, 4.65)	*	n/a		
	15-21 days: 2nd dose	*	1.77 (0.62, 5.07)	*	1.08 (0.26, 4.50)	*	n/a		
	22-28 days: 2nd dose	*	0.95 (0.22, 4.00)	*	1.18 (0.28, 4.97)	*	n/a		
**Cardiac arrhythmia**	Baseline	5520	1.00	11531	1.00	924	1.00	2290	1.00
	-28 to -1 days: 1st dose/positive test	1036	0.87 (0.81, 0.93)	1787	0.90 (0.86, 0.95)	147	1.00 (0.84, 1.19)	283	1.24 (1.09, 1.40)

	0 day: 1st dose/positive test	17	0.39 (0.24, 0.63)	25	0.36 (0.25, 0.54)	6	1.25 (0.56, 2.79)	189	21.63 (18.62, 25.14)
	1-7 days: 1st dose/positive test	352	1.15 (1.03, 1.28)	364	0.76 (0.69, 0.85)	29	0.88 (0.61, 1.27)	378	6.14 (5.50, 6.87)
	8-14 days: 1st dose/positive test	274	0.88 (0.77, 0.99)	459	0.98 (0.89, 1.07)	25	0.78 (0.53, 1.17)	258	4.14 (3.63, 4.72)
	15-21 days: 1st dose/positive test	279	0.89 (0.79, 1.01)	405	0.89 (0.81, 0.99)	27	0.92 (0.63, 1.35)	118	1.82 (1.51, 2.19)
	22-28 days: 1st dose/positive test	267	0.85 (0.75, 0.96)	411	0.96 (0.87, 1.06)	27	1.01 (0.69, 1.49)	114	1.69 (1.40, 2.04)
	-28 to -1 days: 2nd dose	839	0.76 (0.71, 0.83)	659	0.76 (0.70, 0.82)	18	0.67 (0.42, 1.09)		
	0 day: 2nd dose	20	0.51 (0.33, 0.80)	15	0.46 (0.27, 0.76)	*	n/a		
	1-7 days: 2nd dose	256	0.96 (0.84, 1.09)	246	1.12 (0.98, 1.27)	10	2.09 (1.10, 3.94)		
	8-14 days: 2nd dose	268	1.03 (0.91, 1.17)	232	1.14 (1.00, 1.30)	5	1.38 (0.57, 3.36)		
	15-21 days: 2nd dose	248	0.98 (0.86, 1.12)	178	0.96 (0.83, 1.12)	*	n/a		
	22-28 days: 2nd dose	218	0.89 (0.78, 1.02)	146	0.87 (0.74, 1.03)	*	1.39 (0.34, 5.62)		
**Age >= 40**									
**Myocarditis**	Baseline	455	1.00	278	1.00	6	1.00	82	1.00
	-28 to -1 days: 1st dose/positive test	73	0.81 (0.62, 1.05)	34	0.70 (0.48, 1.03)	*	n/a	25	3.40 (2.12, 5.46)
	0 day: 1st dose/positive test	*	0.66 (0.16, 2.68)	*	n/a	*	n/a	31	100.44 (64.84, 155.60)
	1-7 days: 1st dose/positive test	33	1.49 (1.03, 2.17)	13	0.91 (0.51, 1.62)	*	n/a	58	26.08 (18.16, 37.44)
	8-14 days: 1st dose/positive test	33	1.38 (0.95, 2.01)	12	0.84 (0.46, 1.54)	*	n/a	36	15.61 (10.35, 23.54)
	15-21 days: 1st dose/positive test	26	1.08 (0.71, 1.63)	16	1.11 (0.66, 1.88)	*	n/a	15	6.27 (3.58, 11.00)
	22-28 days: 1st dose/positive test	29	1.19 (0.81, 1.77)	20	1.49 (0.93, 2.40)	*	n/a	8	3.20 (1.54, 6.67)
	-28 to -1 days: 2nd dose	53	0.69 (0.51, 0.94)	41	0.91 (0.64, 1.29)	*	n/a		
	0 day: 2nd dose	*	0.72 (0.18, 2.92)	*	n/a	*	n/a		

	1-7 days: 2nd dose	11	0.58 (0.31, 1.06)	9	0.73 (0.37, 1.44)	*	n/a		
	8-14 days: 2nd dose	17	0.92 (0.56, 1.50)	13	1.04 (0.59, 1.83)	*	n/a		
	15-21 days: 2nd dose	17	0.95 (0.58, 1.56)	12	0.99 (0.55, 1.78)	*	n/a		
	22-28 days: 2nd dose	18	1.04 (0.64, 1.68)	12	1.01 (0.56, 1.83)	*	n/a		
**Pericarditis**	Baseline	524	1.00	307	1.00	*	1.00	69	1.00
	-28 to -1 days: 1st dose/positive test	57	0.59 (0.44, 0.79)	26	0.60 (0.39, 0.90)	*	2.90 (0.18, 46.58)	24	4.38 (2.68, 7.18)
	0 day: 1st dose/positive test	*	n/a	*	n/a	*	n/a	9	42.93 (20.99, 87.78)
	1-7 days: 1st dose/positive test	17	0.62 (0.38, 1.01)	7	0.56 (0.26, 1.20)	*	n/a	9	6.15 (3.01, 12.54)
	8-14 days: 1st dose/positive test	32	1.10 (0.76, 1.59)	7	0.52 (0.24, 1.11)	*	n/a	9	5.82 (2.86, 11.85)
	15-21 days: 1st dose/positive test	22	0.71 (0.46, 1.09)	13	0.94 (0.53, 1.67)	*	n/a	*	2.42 (0.88, 6.70)
	22-28 days: 1st dose/positive test	24	0.73 (0.48, 1.12)	16	1.10 (0.66, 1.85)	*	n/a	*	1.12 (0.27, 4.58)
	-28 to -1 days: 2nd dose	52	0.41 (0.30, 0.55)	28	0.41 (0.28, 0.62)	*	n/a		
	0 day: 2nd dose	*	n/a	*	1.07 (0.34, 3.35)	*	n/a		
	1-7 days: 2nd dose	31	1.00 (0.68, 1.45)	9	0.48 (0.24, 0.93)	*	n/a		
	8-14 days: 2nd dose	24	0.78 (0.51, 1.19)	13	0.71 (0.40, 1.24)	*	n/a		
	15-21 days: 2nd dose	32	1.05 (0.73, 1.53)	19	1.02 (0.64, 1.64)	*	n/a		
	22-28 days: 2nd dose	13	0.45 (0.26, 0.79)	24	1.33 (0.87, 2.03)	*	n/a		
**Cardiac arrhythmia**	Baseline	112836	1.00	102437	1.00	297	1.00	12829	1.00
	-28 to -1 days: 1st dose/positive test	16526	0.77 (0.76, 0.78)	10980	0.69 (0.68, 0.71)	37	0.64 (0.46, 0.91)	6335	5.49 (5.32, 5.67)
	0 day: 1st dose/positive test	272	0.33 (0.29, 0.37)	207	0.32 (0.28, 0.37)	*	1.50 (0.48, 4.70)	3658	78.56 (75.62, 81.61)
	1-7 days: 1st dose/positive test	5521	0.93 (0.91, 0.96)	3594	0.79 (0.76, 0.81)	9	0.66 (0.34, 1.27)	4215	12.72 (12.27, 13.19)
	8-14 days: 1st dose/positive test	5707	0.93 (0.90, 0.96)	4378	0.91 (0.88, 0.94)	15	1.13 (0.67, 1.90)	2385	7.00 (6.69, 7.32)

	15-21 days: 1st dose/positive test	5964	0.95 (0.93, 0.98)	4566	0.92 (0.90, 0.95)	16	1.23 (0.74, 2.05)	848	2.38 (2.22, 2.56)
	22-28 days: 1st dose/positive test	5861	0.92 (0.90, 0.95)	4182	0.88 (0.85, 0.91)	8	0.64 (0.32, 1.30)	624	1.66 (1.54, 1.80)
	-28 to -1 days: 2nd dose	17756	0.75 (0.74, 0.77)	14351	0.75 (0.73, 0.76)	20	0.62 (0.39, 0.98)		
	0 day: 2nd dose	245	0.29 (0.25, 0.32)	242	0.32 (0.28, 0.36)	*	n/a		
	1-7 days: 2nd dose	4980	0.84 (0.82, 0.86)	4491	0.84 (0.81, 0.86)	12	1.76 (0.98, 3.16)		
	8-14 days: 2nd dose	5663	0.97 (0.94, 0.99)	5056	0.94 (0.91, 0.96)	8	1.34 (0.66, 2.72)		
	15-21 days: 2nd dose	5771	1.01 (0.98, 1.03)	5317	0.99 (0.96, 1.02)	5	1.02 (0.42, 2.47)		
	22-28 days: 2nd dose	5615	1.00 (0.98, 1.03)	5281	0.99 (0.96, 1.02)	5	1.37 (0.56, 3.35)		

Supplementary table 3b: Incidence rate ratios (IRR 95% CI) by age groups (16-29; 30-39; 40+) for the outcomes in pre-defined risk periods immediately before and after exposure to vaccination and before and after a positive SARS-CoV-2 test result, adjusted for calendar time from December 1 2020 to August 24 2021 (cells with < 5 are suppressed).

		ChAdOx1 vaccine		BNT162b2 mRNA vaccine		mRNA-1273 vaccine		Positive SARS-CoV-2 test	
	**Time period**	**events**	**IRR (95% CI)**	**events**	**IRR (95% CI)**	**events**	**IRR (95% CI)**	**events**	**IRR (95% CI)**
**Age 16-29**									
**Myocarditis**	Baseline	43	1.00	61	1.00	9	1.00	19	1.00
	-28 to -1 days: 1st dose/positive test	5	0.52 (0.19, 1.38)	8	0.82 (0.38, 1.78)	*	1.27 (0.26, 6.33)	*	1.73 (0.57, 5.30)
	0 day: 1st dose/positive test	*	n/a	*	n/a	*	n/a	*	n/a
	1-28 days: 1st dose/positive test	9	1.01 (0.46, 2.22)	11	1.11 (0.56, 2.21)	6	5.20 (1.70, 15.94)	7	2.83 (1.14, 7.03)
	-28 to -1 days: 2nd dose	*	n/a	*	0.87 (0.26, 2.96)	*	n/a		
	0 day: 2nd dose	*	n/a	*	n/a	*	n/a		

	1-28 days: 2nd dose	10	1.54 (0.70, 3.37)	8	2.88 (1.24, 6.72)	*	74.39 (5.28, 1048.75)		
**Pericarditis**	Baseline	29	1.00	58	1.00	5	1.00	19	1.00
	-28 to -1 days: 1st dose/positive test	*	0.41 (0.12, 1.42)	8	0.88 (0.40, 1.93)	*	n/a	5	2.58 (0.90, 7.38)
	0 day: 1st dose/positive test	*	n/a	*	n/a	*	n/a	*	n/a
	1-28 days: 1st dose/positive test	*	0.41 (0.12, 1.42)	6	0.82 (0.34, 1.98)	*	n/a	*	n/a
	-28 to -1 days: 2nd dose	*	0.38 (0.09, 1.70)	*	1.00 (0.29, 3.49)	*	n/a		
	0 day: 2nd dose	*	n/a	*	n/a	*	n/a		
	1-28 days: 2nd dose	9	2.20 (0.92, 5.25)	6	1.81 (0.69, 4.74)	*	n/a		
**Cardiac arrhythmia**	Baseline	2211	1.00	5832	1.00	479	1.00	1146	1.00
	-28 to -1 days: 1st dose/positive test	420	0.86 (0.77, 0.96)	827	0.88 (0.82, 0.95)	66	0.94 (0.73, 1.22)	148	1.27 (1.07, 1.52)
	0 day: 1st dose/positive test	11	0.62 (0.34, 1.12)	13	0.39 (0.23, 0.68)	*	1.29 (0.41, 4.01)	77	17.60 (13.94, 22.22)
	1-28 days: 1st dose/positive test	498	0.99 (0.89, 1.09)	810	0.94 (0.87, 1.02)	56	1.02 (0.77, 1.36)	333	2.66 (2.34, 3.01)
	-28 to -1 days: 2nd dose	365	0.84 (0.75, 0.95)	263	0.80 (0.70, 0.91)	*	0.78 (0.28, 2.20)		
	0 day: 2nd dose	11	0.72 (0.40, 1.31)	6	0.46 (0.21, 1.04)	*	n/a		
	1-28 days: 2nd dose	416	1.02 (0.92, 1.14)	315	0.99 (0.87, 1.11)	*	0.64 (0.15, 2.64)		
**Age 30-39**									
**Myocarditis**	Baseline	52	1.00	59	1.00	7	1.00	18	1.00
	-28 to -1 days: 1st dose/positive test	*	0.24 (0.07, 0.80)	7	1.15 (0.50, 2.65)	*	n/a	*	1.76 (0.49, 6.25)

	0 day: 1st dose/positive test	*	n/a	*	n/a	*	n/a	5	87.54 (30.18, 253.96)
	1-28 days: 1st dose/positive test	12	0.86 (0.43, 1.73)	22	2.76 (1.57, 4.86)	*	2.14 (0.42, 10.98)	10	5.94 (2.57, 13.71)
	-28 to -1 days: 2nd dose	5	0.68 (0.25, 1.81)	*	1.07 (0.36, 3.17)	*	5.63 (0.85, 37.50)		
	0 day: 2nd dose	*	n/a	*	n/a	*	n/a		
	1-28 days: 2nd dose	11	1.51 (0.73, 3.13)	10	3.95 (1.78, 8.76)	*	n/a		
**Pericarditis**	Baseline	28	1.00	49	1.00	6	1.00	7	1.00
	-28 to -1 days: 1st dose/positive test	*	0.53 (0.18, 1.57)	8	0.96 (0.44, 2.12)	*	1.77 (0.34, 9.30)	*	n/a
	0 day: 1st dose/positive test	*	n/a	*	n/a	*	n/a	*	172.09 (28.03, 1056.70)
	1-28 days: 1st dose/positive test	*	0.58 (0.20, 1.73)	10	0.99 (0.48, 2.04)	*	2.30 (0.43, 12.19)	*	3.19 (0.63, 16.26)
	-28 to -1 days: 2nd dose	*	0.81 (0.27, 2.46)	*	0.77 (0.26, 2.23)	*	n/a		
	0 day: 2nd dose	*	n/a	*	n/a	*	n/a		
	1-28 days: 2nd dose	8	1.51 (0.63, 3.62)	*	0.90 (0.31, 2.64)	*	n/a		
**Cardiac arrhythmia**	Baseline	3309	1.00	5699	1.00	445	1.00	1144	1.00
	-28 to -1 days: 1st dose/positive test	616	0.86 (0.79, 0.94)	960	0.91 (0.84, 0.98)	81	1.04 (0.82, 1.32)	135	1.17 (0.98, 1.40)
	0 day: 1st dose/positive test	6	0.23 (0.10, 0.51)	12	0.33 (0.19, 0.58)	*	1.18 (0.38, 3.67)	112	25.02 (20.55, 30.47)
	1-28 days: 1st dose/positive test	674	0.91 (0.84, 1.00)	829	0.86 (0.79, 0.93)	52	0.79 (0.59, 1.06)	535	4.08 (3.67, 4.53)
	-28 to -1 days: 2nd dose	474	0.71 (0.64, 0.79)	396	0.72 (0.65, 0.81)	14	0.63 (0.36, 1.09)		
	0 day: 2nd dose	9	0.38 (0.20, 0.73)	9	0.45 (0.23, 0.87)	*	n/a		
	1-28 days: 2nd dose	574	0.92 (0.84, 1.02)	487	1.07 (0.97, 1.18)	16	1.77 (1.05, 2.99)		
**Age >=40**									

**Myocarditis**	Baseline	455	1.00	278	1.00	6	1.00	82	1.00
	-28 to -1 days: 1st dose/positive test	73	0.76 (0.58, 1.00)	34	0.68 (0.47, 1.00)	*	n/a	25	3.22 (2.02, 5.15)
	0 day: 1st dose/positive test	*	0.66 (0.16, 2.67)	*	n/a	*	n/a	31	94.81 (61.39, 146.42)
	1-28 days: 1st dose/positive test	121	1.33 (1.06, 1.67)	61	1.12 (0.83, 1.52)	*	n/a	117	12.18 (9.01, 16.46)
	-28 to -1 days: 2nd dose	53	0.70 (0.51, 0.95)	41	0.93 (0.65, 1.32)	*	n/a		
	0 day: 2nd dose	*	0.73 (0.18, 2.95)	0	n/a	*	n/a		
	1-28 days: 2nd dose	63	0.87 (0.65, 1.15)	46	0.96 (0.69, 1.34)	*	2.23 (0.23, 21.46)		
**Pericarditis**	Baseline	524	1.00	307	1.00	*	1.00	69	1.00
	-28 to -1 days: 1st dose/positive test	57	0.59 (0.44, 0.78)	26	0.60 (0.39, 0.90)	*	n/a	24	4.30 (2.63, 7.03)
	0 day: 1st dose/positive test	*	n/a	*	n/a	*	n/a	9	42.16 (20.65, 86.07)
	1-28 days: 1st dose/positive test	95	0.79 (0.62, 1.00)	43	0.80 (0.57, 1.12)	*	n/a	24	3.72 (2.30, 6.00)
	-28 to -1 days: 2nd dose	52	0.40 (0.30, 0.55)	28	0.41 (0.28, 0.61)	*	n/a		
	0 day: 2nd dose	*	n/a	*	1.07 (0.34, 3.35)	*	n/a		
	1-28 days: 2nd dose	100	0.82 (0.65, 1.04)	65	0.88 (0.66, 1.16)	*	n/a		
**Cardiac arrhythmia**	Baseline	112836	1.00	102437	1.00	297	1.00	12829	1.00
	-28 to -1 days: 1st dose/positive test	16526	0.75 (0.74, 0.76)	10980	0.68 (0.67, 0.70)	37	0.64 (0.46, 0.91)	6335	5.35 (5.18, 5.52)
	0 day: 1st dose/positive test	272	0.33 (0.29, 0.37)	207	0.32 (0.28, 0.37)	*	1.50 (0.48, 4.69)	3658	76.38 (73.54, 79.34)
	1-28 days: 1st dose/positive test	23053	0.94 (0.93, 0.96)	16720	0.88 (0.87, 0.90)	48	0.91 (0.67, 1.24)	8072	5.71 (5.54, 5.87)
	-28 to -1 days: 2nd dose	17756	0.76 (0.74, 0.77)	14351	0.75 (0.74, 0.77)	20	0.62 (0.39, 0.98)		

	0 day: 2nd dose	245	0.29 (0.25, 0.32)	242	0.32 (0.28, 0.36)	*	n/a		
	1-28 days: 2nd dose	22029	0.95 (0.94, 0.97)	20145	0.94 (0.93, 0.96)	30	1.40 (0.95, 2.07)		

Supplementary table 4: Incidence rate ratios (IRR 95% CI) for the outcomes in pre-defined risk periods immediately before and after exposure to vaccination adjusted for calendar time from December 1 2020 to August 24 2021 (cells with < 5 are suppressed), including only people who did not have a SARS-CoV-2 positive test. Model for pericarditis does not converge with the mRNA-1273 vaccine.

		ChAdOx1 vaccine		BNT162b2 mRNA vaccine		mRNA-1273 vaccine	
	**Time period**	**events**	**IRR (95% CI)**	**events**	**IRR (95% CI)**	**events**	**IRR (95% CI)**
**Myocarditis**	Baseline	387	1.00	314	1.00	18	1.00
	-28 to -1 days: 1st dose/positive test	62	0.82 (0.62, 1.09)	42	0.84 (0.60, 1.17)	*	0.61 (0.14, 2.63)
	0 day: 1st dose/positive test	*	0.69 (0.17, 2.80)	*	1.53 (0.49, 4.78)	*	n/a
	1-7 days: 1st dose/positive test	38	1.91 (1.35, 2.70)	22	1.58 (1.02, 2.44)	5	6.30 (2.32, 17.13)
	8-14 days: 1st dose/positive test	24	1.15 (0.75, 1.76)	18	1.31 (0.81, 2.12)	*	n/a
	15-21 days: 1st dose/positive test	22	1.02 (0.65, 1.58)	17	1.22 (0.75, 2.01)	*	n/a
	22-28 days: 1st dose/positive test	23	1.04 (0.67, 1.60)	17	1.28 (0.78, 2.10)	*	n/a
	-28 to -1 days: 2nd dose	53	0.71 (0.52, 0.96)	44	1.02 (0.73, 1.42)	*	1.90 (0.39, 9.31)
	0 day: 2nd dose	*	0.74 (0.18, 2.99)	*	n/a	*	n/a
	1-7 days: 2nd dose	11	0.59 (0.32, 1.08)	20	1.75 (1.10, 2.79)	*	19.86 (5.52, 71.47)
	8-14 days: 2nd dose	26	1.41 (0.93, 2.12)	15	1.34 (0.79, 2.27)	*	n/a
	15-21 days: 2nd dose	18	0.99 (0.61, 1.60)	13	1.20 (0.68, 2.12)	*	n/a
	22-28 days: 2nd dose	18	0.99 (0.61, 1.60)	11	1.04 (0.57, 1.93)	*	n/a
	1-28 days: 1st dose/positive test	107	1.27 (1.00, 1.60)	74	1.34 (1.03, 1.74)	7	2.37 (0.98, 5.75)

	1-28 days: 2nd dose	73	1.00 (0.76, 1.30)	59	1.35 (1.00, 1.82)	*	8.70 (2.35, 32.11)
**Pericarditis**	Baseline	501	1.00	376	1.00	-	-
	-28 to -1 days: 1st dose/positive test	57	0.56 (0.42, 0.75)	38	0.63 (0.45, 0.89)	-	-
	0 day: 1st dose/positive test	*	n/a	*	0.80 (0.20, 3.23)	-	-
	1-7 days: 1st dose/positive test	14	0.49 (0.29, 0.84)	9	0.52 (0.27, 1.02)	-	-
	8-14 days: 1st dose/positive test	29	0.96 (0.65, 1.41)	6	0.35 (0.16, 0.79)	-	-
	15-21 days: 1st dose/positive test	19	0.59 (0.37, 0.94)	16	0.94 (0.57, 1.57)	-	-
	22-28 days: 1st dose/positive test	24	0.72 (0.48, 1.10)	16	0.90 (0.54, 1.49)	-	-
	-28 to -1 days: 2nd dose	51	0.40 (0.29, 0.54)	31	0.42 (0.29, 0.61)	-	-
	0 day: 2nd dose	*	n/a	*	0.65 (0.16, 2.64)	-	-
	1-7 days: 2nd dose	35	1.13 (0.80, 1.62)	12	0.58 (0.33, 1.04)	-	-
	8-14 days: 2nd dose	23	0.77 (0.50, 1.18)	14	0.70 (0.41, 1.21)	-	-
	15-21 days: 2nd dose	33	1.12 (0.78, 1.60)	21	1.06 (0.68, 1.67)	-	-
	22-28 days: 2nd dose	13	0.46 (0.26, 0.80)	25	1.32 (0.87, 1.99)	-	-
	1-28 days: 1st dose/positive test	86	0.69 (0.54, 0.88)	47	0.68 (0.50, 0.93)	-	-
	1-28 days: 2nd dose	104	0.87 (0.69, 1.09)	72	0.90 (0.69, 1.18)	-	-
**Cardiac arrhythmia**	Baseline	101126	1.00	105775	1.00	1012	1.00
	-28 to -1 days: 1st dose/positive test	15105	0.80 (0.79, 0.82)	11577	0.74 (0.72, 0.75)	167	0.92 (0.78, 1.08)
	0 day: 1st dose/positive test	244	0.33 (0.29, 0.37)	202	0.32 (0.28, 0.37)	8	1.30 (0.65, 2.61)
	1-7 days: 1st dose/positive test	4945	0.92 (0.90, 0.95)	3276	0.75 (0.72, 0.77)	31	0.74 (0.52, 1.06)
	8-14 days: 1st dose/positive test	5087	0.91 (0.89, 0.94)	3999	0.88 (0.86, 0.91)	38	0.95 (0.69, 1.32)
	15-21 days: 1st dose/positive test	5442	0.95 (0.92, 0.97)	4286	0.91 (0.88, 0.94)	39	1.04 (0.75, 1.43)
	22-28 days: 1st dose/positive test	5509	0.93 (0.90, 0.95)	4117	0.89 (0.86, 0.92)	32	0.92 (0.65, 1.31)
	-28 to -1 days: 2nd dose	17321	0.76 (0.74, 0.77)	14319	0.75 (0.73, 0.76)	35	0.65 (0.46, 0.92)
	0 day: 2nd dose	249	0.30 (0.26, 0.34)	242	0.32 (0.28, 0.36)	*	n/a

	1-7 days: 2nd dose	4837	0.84 (0.81, 0.86)	4479	0.84 (0.82, 0.87)	22	2.06 (1.34, 3.17)
	8-14 days: 2nd dose	5515	0.97 (0.94, 0.99)	5047	0.94 (0.92, 0.97)	13	1.48 (0.85, 2.59)
	15-21 days: 2nd dose	5606	1.00 (0.97, 1.03)	5240	0.98 (0.95, 1.01)	6	0.90 (0.40, 2.01)
	22-28 days: 2nd dose	5456	1.00 (0.97, 1.03)	5201	0.98 (0.96, 1.01)	7	1.52 (0.72, 3.22)
	1-28 days: 1st dose/positive test	20983	0.93 (0.91, 0.94)	15678	0.86 (0.85, 0.88)	140	0.90 (0.76, 1.08)
	1-28 days: 2nd dose	21414	0.95 (0.93, 0.96)	19967	0.94 (0.93, 0.96)	48	1.56 (1.15, 2.12)

Supplementary table 5: Incidence rate ratios (IRR 95% CI) for the outcomes in 1-28 days risk periods immediately before and after exposure to vaccination, adjusted for calendar time from December 1 2020 to August 24 2021 (cells with < 5 are suppressed), including only people who had a SARS-CoV-2 positive test result prior vaccination. Numbers of events in those vaccinated with mRNA-1273 vaccine were too small for meaningful results.

		ChAdOx1 vaccine		BNT162b2 mRNA vaccine		Positive SARS-CoV-2 test	
	**Time period**	**events**	**IRR (95% CI)**	**events**	**IRR (95% CI)**	**events**	**IRR (95% CI)**
**Myocarditis**	Baseline	145	1.00	66	1.00	95	1.00
	-28 to -1 days: 1st dose/positive test	19	0.52 (0.31, 0.88)	7	0.49 (0.21, 1.14)	25	1.63 (0.96, 2.78)
	0 day: 1st dose/positive test	*	n/a	*	n/a	23	38.14 (22.32, 65.18)
	1-28 days: 1st dose/positive test	18	1.09 (0.63, 1.91)	7	0.96 (0.42, 2.20)	106	6.59 (4.54, 9.56)
	-28 to -1 days: 2nd dose	*	0.35 (0.12, 0.99)	*	0.90 (0.31, 2.63)		
	0 day: 2nd dose	*	n/a	*	n/a		
	1-28 days: 2nd dose	9	0.91 (0.43, 1.94)	*	0.52 (0.12, 2.23)		
**Pericarditis**	Baseline	72	1.00	32	1.00	79	1.00
	-28 to -1 days: 1st dose/positive test	7	0.50 (0.22, 1.14)	*	0.54 (0.16, 1.81)	24	3.38 (1.80, 6.34)
	0 day: 1st dose/positive test	*	n/a	*	n/a	6	19.96 (7.89, 50.45)
	1-28 days: 1st dose/positive test	11	1.02 (0.50, 2.07)	7	1.43 (0.61, 3.36)	19	2.12 (1.15, 3.91)
	-28 to -1 days: 2nd dose	*	0.31 (0.09, 1.03)	*	1.52 (0.49, 4.70)		

	0 day: 2nd dose	*	n/a	*	n/a		
	1-28 days: 2nd dose	11	1.10 (0.54, 2.24)	*	n/a		
**Cardiac arrhythmia**	Baseline	14933	1.00	5895	1.00	10928	1.00
	-28 to -1 days: 1st dose/positive test	2025	0.55 (0.52, 0.58)	883	0.61 (0.56, 0.66)	4120	2.78 (2.64, 2.91)
	0 day: 1st dose/positive test	24	0.29 (0.19, 0.43)	15	0.41 (0.25, 0.68)	2402	39.09 (37.03, 41.25)
	1-28 days: 1st dose/positive test	1559	0.78 (0.74, 0.83)	694	0.81 (0.74, 0.88)	6761	4.05 (3.89, 4.22)
	-28 to -1 days: 2nd dose	1008	0.70 (0.65, 0.75)	462	0.78 (0.71, 0.87)		
	0 day: 2nd dose	11	0.21 (0.12, 0.39)	6	0.27 (0.12, 0.61)		
	1-28 days: 2nd dose	1269	0.95 (0.89, 1.01)	580	1.02 (0.93, 1.12)		

Supplementary table 6: Incidence rate ratios (IRR 95% CI) for subgroups of arrhythmia in pre-defined risk periods immediately before and after exposure to vaccination and before and after a Positive SARS-CoV-2 test result, adjusted for calendar time from December 1 2020 until August 24 2021, 2021 (cells with < 5 are suppressed).

		**ChAdOx1 vaccine**		**BNT162b2 mRNA vaccine**		**mRNA-1273 vaccine**		**Positive SARS-CoV-2 test**	
	Time period	events	IRR (95% CI)	events	IRR (95% CI)	events	IRR (95% CI)	events	IRR (95% CI)
**Atrial fibrillation and flutter**	Baseline	69651	1.00	69639	1.00	143	1.00	7641	1.00
	-28 to -1 days: 1st dose/positive test	9620	0.74 (0.72, 0.75)	7344	0.68 (0.67, 0.70)	29	1.17 (0.78, 1.75)	4335	6.32 (6.08, 6.58)
	0 day: 1st dose/positive test	157	0.31 (0.27, 0.36)	129	0.30 (0.25, 0.35)	*	2.36 (0.58, 9.53)	2327	83.39 (79.47, 87.51)
	1-7 days: 1st dose/positive test	3249	0.89 (0.86, 0.93)	2474	0.79 (0.76, 0.83)	*	0.69 (0.26, 1.87)	2412	12.17 (11.61, 12.76)
	8-14 days: 1st dose/positive test	3465	0.92 (0.89, 0.95)	3003	0.92 (0.89, 0.96)	6	1.08 (0.48, 2.45)	1203	5.92 (5.56, 6.29)
	15-21 days: 1st dose/positive test	3565	0.93 (0.90, 0.96)	3061	0.91 (0.88, 0.95)	11	2.06 (1.11, 3.82)	464	2.18 (1.99, 2.40)
	22-28 days: 1st dose/positive test	3579	0.92 (0.89, 0.95)	2806	0.88 (0.84, 0.91)	5	0.85 (0.31, 2.30)	341	1.52 (1.36, 1.70)
	-28 to -1 days: 2nd dose	10507	0.72 (0.70, 0.74)	9401	0.73 (0.71, 0.74)	8	0.83 (0.40, 1.73)		

	0 day: 2nd dose	148	0.28 (0.24, 0.33)	156	0.30 (0.26, 0.35)	*	n/a		
	1-7 days: 2nd dose	2983	0.81 (0.78, 0.84)	3035	0.83 (0.80, 0.86)	*	n/a		
	8-14 days: 2nd dose	3527	0.97 (0.94, 1.00)	3402	0.93 (0.89, 0.96)	*	n/a		
	15-21 days: 2nd dose	3463	0.97 (0.94, 1.01)	3613	0.99 (0.95, 1.02)	*	n/a		
	22-28 days: 2nd dose	3480	1.00 (0.97, 1.04)	3576	0.99 (0.95, 1.02)	*	n/a		
	1-28 days: 1st dose/positive test	13858	0.92 (0.90, 0.94)	11344	0.88 (0.86, 0.90)	26	1.16 (0.76, 1.79)	4420	5.23 (5.04, 5.44)
	1-28 days: 2nd dose	13453	0.94 (0.92, 0.96)	13626	0.94 (0.92, 0.96)	*	0.35 (0.09, 1.45)		
**AV block and conduction disorders**	Baseline	34779	1.00	34679	1.00	139	1.00	4572	1.00
	-28 to -1 days: 1st dose/positive test	4985	0.77 (0.75, 0.80)	3478	0.68 (0.65, 0.71)	17	0.70 (0.42, 1.17)	2154	5.49 (5.20, 5.79)
	0 day: 1st dose/positive test	115	0.46 (0.38, 0.55)	70	0.34 (0.27, 0.42)	*	n/a	1139	70.99 (66.37, 75.93)
	1-7 days: 1st dose/positive test	1747	0.96 (0.91, 1.01)	1157	0.77 (0.73, 0.82)	*	0.36 (0.09, 1.47)	1198	10.47 (9.80, 11.18)
	8-14 days: 1st dose/positive test	1713	0.90 (0.86, 0.95)	1415	0.90 (0.85, 0.95)	6	1.11 (0.49, 2.53)	677	5.73 (5.28, 6.23)
	15-21 days: 1st dose/positive test	1912	0.98 (0.94, 1.03)	1444	0.89 (0.84, 0.94)	5	0.98 (0.40, 2.40)	247	1.99 (1.75, 2.27)
	22-28 days: 1st dose/positive test	1761	0.89 (0.85, 0.93)	1336	0.85 (0.81, 0.90)	*	0.44 (0.11, 1.77)	168	1.28 (1.10, 1.49)
	-28 to -1 days: 2nd dose	5516	0.75 (0.72, 0.77)	4730	0.74 (0.72, 0.77)	*	0.38 (0.12, 1.21)		
	0 day: 2nd dose	87	0.32 (0.26, 0.40)	87	0.34 (0.28, 0.42)	*	n/a		
	1-7 days: 2nd dose	1647	0.89 (0.84, 0.93)	1531	0.86 (0.81, 0.90)	*	n/a		
	8-14 days: 2nd dose	1729	0.95 (0.90, 0.99)	1714	0.96 (0.91, 1.00)	*	n/a		
	15-21 days: 2nd dose	1790	1.00 (0.95, 1.05)	1739	0.97 (0.93, 1.02)	*	n/a		
	22-28 days: 2nd dose	1666	0.95 (0.90, 1.00)	1694	0.96 (0.91, 1.01)	*	n/a		

	1-28 days: 1st dose/positive test	7133	0.94 (0.91, 0.97)	5352	0.86 (0.83, 0.89)	15	0.73 (0.42, 1.25)	2290	4.65 (4.42, 4.90)
	1-28 days: 2nd dose	6832	0.94 (0.92, 0.97)	6678	0.94 (0.91, 0.97)	*	0.87 (0.31, 2.41)		
**Ventricular tachycardia**	Baseline	2655	1.00	2184	1.00	12	1.00	344	1.00
	-28 to -1 days: 1st dose/positive test	419	0.83 (0.74, 0.93)	225	0.69 (0.60, 0.80)	*	1.37 (0.38, 4.91)	120	4.03 (3.24, 5.01)
	0 day: 1st dose/positive test	*	0.10 (0.02, 0.40)	6	0.45 (0.20, 1.01)	*	n/a	50	40.91 (30.22, 55.39)
	1-7 days: 1st dose/positive test	133	0.92 (0.77, 1.11)	72	0.76 (0.59, 0.96)	*	n/a	42	4.84 (3.50, 6.71)
	8-14 days:1st dose/positive test	133	0.89 (0.75, 1.07)	92	0.92 (0.74, 1.14)	*	4.08 (0.90, 18.52)	16	1.81 (1.09, 3.00)
	15-21 days: 1stdose/positive test	177	1.15 (0.98, 1.35)	94	0.92 (0.74, 1.14)	*	n/a	23	2.53 (1.65, 3.87)
	22-28 days: 1st dose/positive test	152	0.97 (0.82, 1.15)	111	1.06 (0.87, 1.29)	*	n/a	14	1.47 (0.86, 2.52)
	-28 to -1 days: 2nd dose	445	0.74 (0.67, 0.83)	282	0.67 (0.58, 0.76)	*	n/a		
	0 day: 2nd dose	7	0.32 (0.15, 0.68)	*	0.12 (0.03, 0.49)	*	n/a		
	1-7 days: 2nd dose	119	0.80 (0.66, 0.97)	109	0.96 (0.78, 1.16)	*	n/a		
	8-14 days: 2nd dose	139	0.96 (0.80, 1.14)	119	1.03 (0.85, 1.25)	*	n/a		
	15-21 days: 2nd dose	150	1.06 (0.89, 1.25)	106	0.94 (0.77, 1.14)	*	n/a		
	22-28 days: 2nd dose	155	1.13 (0.96, 1.34)	103	0.93 (0.76, 1.14)	*	n/a		
	1-28 days: 1st dose/positive test	595	0.99 (0.89, 1.09)	369	0.92 (0.82, 1.04)	*	2.11 (0.67, 6.66)	95	2.64 (2.10, 3.34)
	1-28 days: 2nd dose	563	0.98 (0.89, 1.08)	437	0.97 (0.87, 1.08)	*	n/a		
**Ventricular fibrillation**	Baseline	1061	1.00	721	1.00	7	1.00	103	1.00
	-28 to -1 days: 1st dose/positive test	150	0.69 (0.57, 0.83)	73	0.65 (0.50, 0.84)	*	n/a	43	5.02 (3.45, 7.30)

	0 day: 1st dose/positive test	*	0.46 (0.17, 1.23)	*	0.66 (0.21, 2.06)	*	n/a	23	64.48 (40.45, 102.79)
	1-7 days: 1st dose/positive test	52	0.83 (0.62, 1.10)	30	0.92 (0.63, 1.34)	*	n/a	12	4.66 (2.54, 8.55)
	8-14 days:1st dose/positive test	61	0.95 (0.73, 1.24)	30	0.84 (0.58, 1.22)	*	n/a	8	3.09 (1.49, 6.38)
	15-21 days: 1stdose/positive test	55	0.82 (0.62, 1.09)	35	0.96 (0.68, 1.37)	*	n/a	9	3.36 (1.69, 6.67)
	22-28 days: 1st dose/positive test	51	0.74 (0.55, 0.98)	24	0.65 (0.43, 0.98)	*	n/a	9	3.16 (1.59, 6.27)
	-28 to -1 days: 2nd dose	111	0.46 (0.38, 0.57)	64	0.44 (0.34, 0.58)	*	n/a		
	0 day: 2nd dose	*	n/a	*	n/a	*	n/a		
	1-7 days: 2nd dose	50	0.87 (0.65, 1.17)	31	0.84 (0.58, 1.21)	*	n/a		
	8-14 days: 2nd dose	47	0.86 (0.64, 1.17)	37	1.00 (0.71, 1.41)	*	n/a		
	15-21 days: 2nd dose	59	1.13 (0.86, 1.48)	48	1.34 (0.99, 1.81)	*	n/a		
	22-28 days: 2nd dose	68	1.35 (1.05, 1.74)	34	0.99 (0.70, 1.41)	*	n/a		
	1-28 days: 1st dose/positive test	219	0.84 (0.71, 0.98)	119	0.84 (0.68, 1.04)	*	n/a	38	3.54 (2.42, 5.18)
	1-28 days: 2nd dose	224	1.05 (0.89, 1.23)	150	1.05 (0.87, 1.27)	*	n/a		
**Other cardiac arrhythmia**s	Baseline	40922	1.00	37232	1.00	1018	1.00	7347	1.00
	-28 to -1 days: 1st dose/positive test	6569	0.81 (0.79, 0.83)	4548	0.76 (0.73, 0.78)	146	0.84 (0.71, 1.00)	1841	2.79 (2.65, 2.95)
	0 day: 1st dose/positive test	122	0.40 (0.33, 0.47)	85	0.37 (0.30, 0.46)	6	1.04 (0.47, 2.33)	1148	43.88 (41.17, 46.77)
	1-7 days: 1st dose/positive test	2176	0.99 (0.95, 1.03)	1222	0.75 (0.71, 0.79)	32	0.80 (0.56, 1.14)	1686	9.06 (8.57, 9.56)
	8-14 days: 1st dose/positive test	2127	0.93 (0.89, 0.98)	1437	0.85 (0.81, 0.90)	29	0.76 (0.52, 1.10)	1154	6.00 (5.63, 6.39)
	15-21 days: 1stdose/positive test	2179	0.94 (0.90, 0.98)	1530	0.89 (0.85, 0.94)	31	0.85 (0.60, 1.22)	436	2.16 (1.96, 2.38)

	22-28 days: 1st dose/positive test	2119	0.90 (0.86, 0.94)	1427	0.86 (0.81, 0.90)	28	0.86 (0.58, 1.28)	398	1.87 (1.69, 2.07)
	-28 to -1 days: 2nd dose	6734	0.79 (0.77, 0.81)	4510	0.77 (0.75, 0.80)	30	0.67 (0.46, 0.97)		
	0 day: 2nd dose	85	0.27 (0.22, 0.34)	70	0.31 (0.24, 0.39)	*	n/a		
	1-7 days: 2nd dose	1887	0.88 (0.84, 0.92)	1401	0.88 (0.84, 0.93)	21	2.32 (1.49, 3.62)		
	8-14 days: 2nd dose	2027	0.95 (0.91, 1.00)	1540	0.97 (0.92, 1.02)	11	1.50 (0.82, 2.74)		
	15-21 days: 2nd dose	2130	1.03 (0.98, 1.07)	1519	0.97 (0.92, 1.02)	5	0.90 (0.37, 2.19)		
	22-28 days: 2nd dose	2025	1.00 (0.96, 1.05)	1487	0.97 (0.92, 1.02)	5	1.32 (0.54, 3.20)		
	1-28 days: 1st dose/positive test	8601	0.95 (0.92, 0.97)	5616	0.84 (0.82, 0.87)	120	0.82 (0.67, 0.99)	3674	4.60 (4.42, 4.79)
	1-28 days: 2nd dose	8069	0.96 (0.94, 0.99)	5947	0.95 (0.92, 0.98)	42	1.64 (1.18, 2.27)		

Supplementary Table 7: Incidence rate ratios (IRR 95% CI) for celiac disease (negative control) and anaphylaxis (positive control) in pre-defined risk periods immediately before and after exposure to vaccination and before and after a positive SARS-CoV-2 result, adjusted for calendar time from December 1 2020 to August 24 2021 (cells with < 5 are suppressed). Model for anaphylaxis with mRNA-1273 vaccine does not converge.

		**Anaphylaxis (positive control)**		**Celiac disease (negative control)**	
**Group**	**Time period**	**events**	**IRR (95% CI)**	**events**	**IRR (95% CI)**
**ChAdOx1 vaccine**	Baseline	726	1.00	3889	1.00
	-28 to -1 days: 1st dose	110	0.78 (0.63, 0.96)	718	0.96 (0.88, 1.05)
	0 day: 1st dose	63	11.83 (9.05, 15.47)	*	0.07 (0.02, 0.28)
	1-7 days: 1st dose	60	1.60 (1.22, 2.11)	166	0.83 (0.71, 0.97)
	8-14 days: 1st dose	29	0.76 (0.52, 1.10)	211	1.03 (0.90, 1.19)
	15-21 days: 1st dose	43	1.09 (0.79, 1.49)	201	0.97 (0.84, 1.12)
	22-28 days: 1st dose	35	0.87 (0.62, 1.24)	205	0.97 (0.84, 1.12)
	-28 to -1 days: 2nd dose	137	0.93 (0.76, 1.13)	756	0.91 (0.84, 0.99)
	0 day: 2nd dose	16	2.97 (1.80, 4.89)	10	0.33 (0.18, 0.61)

	1-7 days: 2nd dose	40	1.06 (0.77, 1.47)	179	0.85 (0.73, 0.99)
	8-14 days: 2nd dose	40	1.06 (0.77, 1.47)	226	1.07 (0.94, 1.23)
	15-21 days: 2nd dose	34	0.92 (0.65, 1.30)	251	1.20 (1.05, 1.36)
	22-28 days: 2nd dose	50	1.39 (1.03, 1.86)	200	0.96 (0.83, 1.11)
	1-28 days: 1st dose	167	1.08 (0.90, 1.29)	783	0.95 (0.88, 1.04)
	1-28 days: 2nd dose	164	1.11 (0.92, 1.33)	856	1.02 (0.94, 1.10)
**BNT162b2 mRNA vaccine**	Baseline	521	1.00	3477	1.00
	-28 to -1 days: 1st dose	62	0.71 (0.54, 0.93)	555	0.95 (0.86, 1.04)
	0 day: 1st dose	39	12.20 (8.78, 16.95)	7	0.32 (0.15, 0.66)
	1-7 days: 1st dose	33	1.46 (1.02, 2.08)	120	0.77 (0.64, 0.93)
	8-14 days: 1st dose	27	1.19 (0.81, 1.76)	145	0.92 (0.77, 1.08)
	15-21 days: 1st dose	25	1.14 (0.76, 1.71)	139	0.86 (0.72, 1.02)
	22-28 days: 1st dose	20	0.94 (0.60, 1.47)	147	0.91 (0.77, 1.08)
	-28 to -1 days: 2nd dose	52	0.94 (0.69, 1.26)	580	0.97 (0.89, 1.07)
	0 day: 2nd dose	11	5.00 (2.73, 9.15)	6	0.26 (0.12, 0.58)
	1-7 days: 2nd dose	22	1.44 (0.93, 2.23)	148	0.93 (0.78, 1.09)
	8-14 days: 2nd dose	17	1.14 (0.70, 1.87)	165	1.03 (0.88, 1.21)
	15-21 days: 2nd dose	19	1.32 (0.82, 2.10)	174	1.11 (0.95, 1.30)
	22-28 days: 2nd dose	15	1.07 (0.64, 1.81)	159	1.03 (0.88, 1.21)
	1-28 days: 1st dose	105	1.18 (0.95, 1.47)	551	0.87 (0.79, 0.96)
	1-28 days: 2nd dose	73	1.26 (0.97, 1.63)	646	1.03 (0.94, 1.12)
**mRNA-1273 vaccine**	Baseline	-	-	78	1.00
	-28 to -1 days: 1st dose	-	-	15	1.06 (0.61, 1.84)
	0 day: 1st dose	-	-	*	n/a
	1-7 days: 1st dose	-	-	*	n/a

	8-14 days: 1st dose	-	-	*	1.23 (0.45, 3.37)
	15-21 days: 1st dose	-	-	5	1.53 (0.62, 3.80)
	22-28 days: 1st dose	-	-	*	1.49 (0.54, 4.10)
	-28 to -1 days: 2nd dose	-	-	8	1.42 (0.66, 3.08)
	0 day: 2nd dose	-	-	*	n/a
	1-7 days: 2nd dose	-	-	*	n/a
	8-14 days: 2nd dose	-	-	*	n/a
	15-21 days: 2nd dose	-	-	*	n/a
	22-28 days: 2nd dose	-	-	*	n/a
	1-28 days: 1st dose	-	-	15	1.11 (0.62, 1.96)
	1-28 days: 2nd dose	-	-	*	n/a
**SARS-CoV-2**	Baseline	187	1.00	621	1.00
	-28 to -1 days: positive test	23	1.30 (0.83, 2.02)	111	1.79 (1.46, 2.21)
	0 day: positive test	14	21.51 (12.36, 37.45)	51	22.12 (16.57, 29.53)
	1-7 days: positive test	10	2.28 (1.20, 4.33)	71	4.39 (3.42, 5.63)
	8-14 days: positive test	*	0.70 (0.22, 2.19)	55	3.33 (2.52, 4.40)
	15-21 days: positive test	*	0.68 (0.22, 2.14)	26	1.54 (1.04, 2.29)
	22-28 days: positive test	8	1.75 (0.86, 3.58)	22	1.25 (0.82, 1.92)
	1-28 days: positive test	24	1.35 (0.87, 2.08)	174	2.59 (2.18, 3.07)

Supplementary table 8a: Incidence rate ratios (IRR 95% CI) for single outcomes in pre-defined risk periods immediately before and after exposure to vaccination, adjusted for calendar time from December 1 2020 to August 24 2021. Comparisons between different sensitivity analyses (cells with < 5 are suppressed).

		Sensitivity 1*	Sensitivity 2*	Sensitivity 3*	Sensitivity 4*	Sensitivity 5*

		events	IRR (95% CI)	events	IRR (95% CI)	events	IRR (95% CI)	events	IRR (95% CI)	events	IRR (95% CI)
**Myocarditis**											
**ChAdOx1 vaccine**	Baseline	523	1.00	286	1.00	535	1.00	359	1.00	550	1.00
	-28 to -1 days: 1st dose	81	0.80 (0.62, 1.03)	-	-	81	0.74 (0.58, 0.95)	81	0.74 (0.58, 0.96)	81	0.70 (0.54, 0.90)
	0 day: 1st dose	*	n/a	*	0.46 (0.11, 1.89)	*	0.55 (0.14, 2.19)	*	0.56 (0.14, 2.24)	*	n/a
	1-7 days: 1st dose	39	1.61 (1.14, 2.27)	47	1.55 (1.07, 2.24)	47	1.77 (1.29, 2.42)	45	1.72 (1.24, 2.38)	*	n/a
	8-14 days: 1st dose	25	0.97 (0.64, 1.47)	35	1.11 (0.74, 1.65)	35	1.22 (0.85, 1.75)	34	1.22 (0.84, 1.77)	*	n/a
	15-21 days: 1st dose	22	0.83 (0.54, 1.29)	30	0.96 (0.63, 1.44)	30	1.03 (0.71, 1.51)	30	1.07 (0.73, 1.59)	*	n/a
	22-28 days: 1st dose	25	0.95 (0.63, 1.44)	30	0.98 (0.65, 1.46)	30	1.03 (0.71, 1.51)	27	0.98 (0.65, 1.47)	*	n/a
	-28 to -1 days: 2nd dose	58	0.66 (0.49, 0.88)	58	0.68 (0.50, 0.92)	58	0.65 (0.49, 0.87)	41	0.68 (0.47, 0.97)	58	0.58 (0.43, 0.78)
	0 day: 2nd dose	*	0.64 (0.16, 2.56)	*	0.66 (0.16, 2.67)	*	0.64 (0.16, 2.56)	*	1.33 (0.33, 5.42)	*	0.57 (0.14, 2.30)
	1-7 days: 2nd dose	11	0.50 (0.27, 0.92)	13	0.62 (0.35, 1.10)	13	0.60 (0.34, 1.05)	7	0.73 (0.33, 1.59)	13	0.54 (0.31, 0.94)
	8-14 days: 2nd dose	26	1.20 (0.80, 1.80)	28	1.37 (0.92, 2.05)	28	1.31 (0.88, 1.94)	9	1.16 (0.58, 2.35)	28	1.18 (0.79, 1.74)
	15-21 days: 2nd dose	18	0.84 (0.52, 1.36)	19	0.97 (0.60, 1.55)	19	0.91 (0.57, 1.45)	8	1.31 (0.62, 2.76)	19	0.82 (0.52, 1.31)
	22-28 days: 2nd dose	24	1.13 (0.74, 1.73)	24	1.25 (0.81, 1.91)	23	1.12 (0.73, 1.71)	6	1.36 (0.58, 3.21)	24	1.06 (0.70, 1.61)
	1-28 days: 1st dose	111	1.11 (0.88, 1.39)	142	1.15 (0.89, 1.50)	142	1.29 (1.05, 1.59)	136	1.28 (1.03, 1.60)	-	-
	1-28 days: 2nd dose	79	0.92 (0.71, 1.19)	84	1.05 (0.81, 1.36)	83	0.99 (0.77, 1.27)	30	1.09 (0.70, 1.69)	84	0.90 (0.70, 1.15)
	Baseline	374	1.00	225	1.00	387	1.00	215	1.00	398	1.00

**BNT162b2 mRNA vaccine**	-28 to -1 days: 1st dose	49	0.79 (0.58, 1.08)			49	0.75 (0.55, 1.02)	40	0.71 (0.50, 1.02)	49	0.74 (0.54, 1.00)
	0 day: 1st dose	*	0.89 (0.22, 3.56)	*	0.89 (0.27, 2.89)	*	1.25 (0.40, 3.89)	*	1.50 (0.48, 4.70)	*	n/a
	1-7 days: 1st dose	23	1.34 (0.87, 2.07)	27	1.01 (0.62, 1.65)	27	1.46 (0.98, 2.19)	16	1.05 (0.62, 1.78)	*	n/a
	8-14 days: 1st dose	20	1.17 (0.74, 1.85)	23	0.85 (0.51, 1.41)	23	1.24 (0.81, 1.92)	17	1.12 (0.66, 1.87)	*	n/a
	15-21 days: 1st dose	17	1.01 (0.61, 1.65)	21	0.83 (0.50, 1.37)	21	1.15 (0.74, 1.81)	16	1.02 (0.60, 1.73)	*	n/a
	22-28 days: 1st dose	19	1.19 (0.75, 1.91)	23	1.03 (0.64, 1.65)	23	1.35 (0.88, 2.07)	18	1.22 (0.74, 2.02)	*	n/a
	-28 to -1 days: 2nd dose	48	0.98 (0.71, 1.34)	48	0.91 (0.65, 1.29)	48	0.97 (0.71, 1.33)	45	0.98 (0.69, 1.41)	48	0.85 (0.62, 1.17)
	0 day: 2nd dose	*	n/a	*	n/a	*	n/a	*	n/a	*	n/a
	1-7 days: 2nd dose	21	1.63 (1.03, 2.56)	23	1.63 (1.03, 2.56)	22	1.70 (1.09, 2.65)	12	1.07 (0.59, 1.97)	23	1.57 (1.02, 2.43)
	8-14 days: 2nd dose	11	0.87 (0.47, 1.59)	15	1.13 (0.66, 1.93)	15	1.19 (0.70, 2.02)	12	1.21 (0.66, 2.22)	15	1.05 (0.62, 1.78)
	15-21 days: 2nd dose	10	0.83 (0.44, 1.58)	14	1.14 (0.65, 1.98)	14	1.18 (0.68, 2.03)	9	1.03 (0.52, 2.07)	14	1.03 (0.60, 1.77)
	22-28 days: 2nd dose	12	1.03 (0.57, 1.85)	12	1.03 (0.57, 1.86)	12	1.04 (0.58, 1.86)	8	1.11 (0.53, 2.31)	12	0.91 (0.51, 1.63)
	1-28 days: 1st dose	79	1.20 (0.93, 1.54)	94	0.91 (0.66, 1.27)	94	1.33 (1.05, 1.68)	67	1.13 (0.84, 1.53)	-	-
	1-28 days: 2nd dose	54	1.11 (0.82, 1.51)	64	1.25 (0.92, 1.69)	63	1.30 (0.98, 1.74)	41	1.13 (0.78, 1.66)	64	1.16 (0.87, 1.54)
**mRNA-1273 vaccine**	Baseline	22	1.00	*	1.00	22	1.00	12	1.00	22	1.00
	-28 to -1 days: 1st dose	*	0.40 (0.09, 1.82)	-	-	*	0.41 (0.09, 1.84)	*	n/a	*	0.37 (0.08, 1.71)
	0 day: 1st dose	0	n/a	*	n/a	*	n/a	*	n/a	*	n/a

	1-7 days: 1st dose	7	8.06 (3.39, 19.14)	7	15.24 (1.65, 140.79)	7	8.33 (3.50, 19.84)	*	n/a	*	n/a
	8-14 days: 1st dose	*	n/a	*	n/a	*	n/a	*	n/a	*	n/a
	15-21 days: 1st dose	*	n/a	*	n/a	*	n/a	*	n/a	*	n/a
	22-28 days: 1st dose	*	n/a	*	n/a	*	n/a	*	n/a	*	n/a
	-28 to -1 days: 2nd dose	*	2.05 (0.42, 10.01)	*	n/a	*	2.18 (0.44, 10.64)		n/a	*	1.51 (0.32, 7.23)
	0 day: 2nd dose	*	n/a	*	n/a	*	n/a	*	n/a	*	n/a
	1-7 days: 2nd dose	*	21.52 (6.03, 76.79)	*	n/a	*	26.48 (7.11, 98.58)	*	n/a	*	15.63 (4.50, 54.37)
	8-14 days: 2nd dose	*	n/a	*	n/a	*	n/a	*	n/a	*	n/a
	15-21 days: 2nd dose	*	n/a	*	n/a	*	n/a	*	n/a	*	n/a
	22-28 days: 2nd dose	*	n/a	*	n/a	*	n/a	*	n/a	*	n/a
	1-28 days: 1st dose	9	2.83 (1.28, 6.27)	9	5.32 (0.60, 47.31)	9	3.01 (1.35, 6.70)	*	n/a	-	-
	1-28 days: 2nd dose	*	9.13 (2.50, 33.40)	*	n/a	*	15.25 (3.80, 61.24)	*	n/a	*	6.41 (1.81, 22.74)
**Pericarditis**											
**ChAdOx1 vaccine**	Baseline	566	1.00	362	1.00	563	1.00	360	1.00	581	1.00
	-28 to -1 days: 1st dose	64	0.56 (0.43, 0.74)	-	-	64	0.54 (0.41, 0.71)	63	0.53 (0.40, 0.70)	64	0.54 (0.41, 0.71)
	0 day: 1st dose	*	n/a	*	n/a	*	n/a	*	n/a	*	n/a
	1-7 days: 1st dose	17	0.54 (0.33, 0.88)	19	0.56 (0.34, 0.92)	19	0.58 (0.37, 0.93)	19	0.60 (0.37, 0.96)	*	n/a
	8-14 days: 1st dose	32	0.98 (0.68, 1.41)	34	0.94 (0.64, 1.39)	34	1.00 (0.70, 1.43)	33	1.01 (0.70, 1.46)	*	n/a
	15-21 days: 1st dose	23	0.67 (0.44, 1.02)	23	0.60 (0.38, 0.94)	23	0.64 (0.42, 0.98)	23	0.68 (0.44, 1.04)	*	n/a

	22-28 days: 1st dose	26	0.74 (0.49, 1.10)	26	0.65 (0.43, 0.99)	26	0.70 (0.47, 1.05)	25	0.73 (0.48, 1.11)	*	n/a
	-28 to -1 days: 2nd dose	58	0.44 (0.33, 0.58)	58	0.42 (0.32, 0.57)	58	0.42 (0.32, 0.56)	41	0.41 (0.29, 0.58)	58	0.42 (0.32, 0.56)
	0 day: 2nd dose	*	n/a	*	n/a	*	n/a	*	n/a	*	0.20 (0.03, 1.45)
	1-7 days: 2nd dose	36	1.11 (0.78, 1.57)	37	1.13 (0.80, 1.60)	37	1.11 (0.79, 1.56)	14	0.80 (0.45, 1.41)	37	1.11 (0.78, 1.56)
	8-14 days: 2nd dose	28	0.87 (0.59, 1.29)	29	0.91 (0.61, 1.34)	28	0.86 (0.58, 1.27)	15	1.02 (0.58, 1.77)	29	0.88 (0.60, 1.30)
	15-21 days: 2nd dose	35	1.11 (0.78, 1.57)	36	1.15 (0.81, 1.63)	36	1.12 (0.79, 1.59)	18	1.47 (0.87, 2.49)	36	1.12 (0.79, 1.59)
	22-28 days: 2nd dose	15	0.49 (0.29, 0.83)	15	0.51 (0.30, 0.85)	14	0.46 (0.27, 0.78)	5	0.58 (0.23, 1.45)	15	0.48 (0.29, 0.81)
	1-28 days: 1st dose	98	0.73 (0.58, 0.92)	102	0.69 (0.53, 0.90)	102	0.73 (0.58, 0.92)	100	0.75 (0.59, 0.96)	-	-
	1-28 days: 2nd dose	114	0.90 (0.72, 1.11)	117	0.93 (0.75, 1.17)	115	0.89 (0.72, 1.11)	52	0.96 (0.67, 1.38)	117	0.90 (0.73, 1.12)
**BNT162b2 mRNA vaccine**	Baseline	407	1.00	268	1.00	402	1.00	208	1.00	414	1.00
	-28 to -1 days: 1st dose	42	0.63 (0.46, 0.88)	-	-	42	0.62 (0.45, 0.86)	38	0.67 (0.47, 0.95)	42	0.61 (0.44, 0.85)
	0 day: 1st dose	*	0.75 (0.19, 3.04)	*	0.54 (0.13, 2.27)	*	0.74 (0.18, 2.97)	*	n/a	*	n/a
	1-7 days: 1st dose	11	0.60 (0.33, 1.09)	11	0.44 (0.22, 0.87)	11	0.58 (0.32, 1.07)	7	0.46 (0.22, 0.99)	*	n/a
	8-14 days: 1st dose	9	0.47 (0.24, 0.92)	9	0.39 (0.19, 0.80)	9	0.46 (0.24, 0.90)	6	0.39 (0.17, 0.88)	*	n/a
	15-21 days: 1st dose	18	0.95 (0.59, 1.53)	19	0.82 (0.49, 1.39)	18	0.93 (0.57, 1.50)	15	0.95 (0.55, 1.64)	*	n/a
	22-28 days: 1st dose	20	1.04 (0.66, 1.65)	20	0.89 (0.54, 1.46)	20	1.02 (0.64, 1.61)	18	1.11 (0.67, 1.83)	*	n/a
	-28 to -1 days: 2nd dose	35	0.48 (0.34, 0.68)	35	0.45 (0.31, 0.65)	35	0.47 (0.33, 0.67)	31	0.48 (0.32, 0.72)	35	0.45 (0.32, 0.65)

	0 day: 2nd dose	*	1.01 (0.32, 3.15)	*	0.90 (0.29, 2.82)	*	0.99 (0.32, 3.08)	*	1.19 (0.38, 3.75)	*	0.96 (0.31, 3.01)
	1-7 days: 2nd dose	12	0.59 (0.33, 1.06)	12	0.54 (0.30, 0.98)	12	0.58 (0.33, 1.04)	8	0.50 (0.24, 1.02)	12	0.57 (0.32, 1.02)
	8-14 days: 2nd dose	16	0.81 (0.49, 1.35)	16	0.75 (0.45, 1.26)	16	0.80 (0.48, 1.33)	13	0.94 (0.53, 1.69)	16	0.78 (0.47, 1.30)
	15-21 days: 2nd dose	20	1.02 (0.64, 1.61)	21	1.03 (0.65, 1.62)	21	1.06 (0.68, 1.66)	15	1.18 (0.68, 2.06)	21	1.03 (0.66, 1.61)
	22-28 days: 2nd dose	26	1.37 (0.91, 2.05)	26	1.34 (0.89, 2.02)	25	1.31 (0.86, 1.98)	14	1.29 (0.73, 2.29)	26	1.31 (0.87, 1.97)
	1-28 days: 1st dose	58	0.77 (0.58, 1.02)	59	0.65 (0.45, 0.94)	58	0.75 (0.57, 1.00)	46	0.74 (0.53, 1.04)	-	-
	1-28 days: 2nd dose	74	0.94 (0.72, 1.22)	75	0.91 (0.69, 1.19)	74	0.93 (0.71, 1.21)	50	0.93 (0.66, 1.31)	75	0.91 (0.70, 1.19)
**mRNA-1273 vaccine**	Baseline	12	1.00	*	1.00	11	1.00	5	1.00	12	1.00
	-28 to -1 days: 1st dose	*	1.61 (0.51, 5.04)	-	-	*	1.78 (0.56, 5.66)	*	n/a	*	1.63 (0.52, 5.12)
	0 day: 1st dose	*	n/a	*	n/a	*	n/a	*	n/a	-	-
	1-7 days: 1st dose	*	n/a	*	n/a	*	n/a	*	n/a	-	-
	8-14 days: 1st dose	*	n/a	*	n/a	*	n/a	*	n/a	-	-
	15-21 days: 1st dose	*	n/a	*	n/a	*	n/a	*	n/a	-	-
	22-28 days: 1st dose	*	n/a	*	n/a	*	n/a	*	n/a	-	-
	-28 to -1 days: 2nd dose	*	n/a	*	n/a	*	n/a	*	n/a	*	n/a
	0 day: 2nd dose	*	n/a	*	n/a	*	n/a	*	n/a	*	n/a
	1-7 days: 2nd dose	*	n/a	*	n/a	*	n/a	*	n/a	*	n/a
	8-14 days: 2nd dose	*	n/a	*	n/a	*	n/a	*	n/a	*	n/a
	15-21 days: 2nd dose	*	n/a	*	n/a	*	n/a	*	n/a	*	n/a

	22-28 days: 2nd dose	*	n/a	*	n/a	*	n/a	-	-	*	n/a
	1-28 days: 1st dose	*	1.61 (0.45, 5.85)	*	n/a	*	1.91 (0.52, 7.03)	*	n/a	-	-
	1-28 days: 2nd dose	*	n/a	*	n/a	*	n/a	*	n/a	*	n/a
**Cardiac arrhythmia**											
**ChAdOx1 vaccine**	Baseline	116100	1.00	76686	1.00	114543	1.00	68219	1.00	118356	1.00
	-28 to -1 days: 1st dose	17335	0.79 (0.77, 0.80)	-	-	17562	0.78 (0.76, 0.79)	17459	0.78 (0.76, 0.79)	17562	0.77 (0.76, 0.78)
	0 day: 1st dose	278	0.33 (0.29, 0.37)	289	0.34 (0.30, 0.38)	289	0.33 (0.30, 0.37)	283	0.33 (0.29, 0.37)	*	n/a
	1-7 days: 1st dose	5647	0.94 (0.91, 0.96)	5873	0.97 (0.94, 1.00)	5871	0.94 (0.92, 0.97)	5789	0.94 (0.92, 0.97)	*	n/a
	8-14 days: 1st dose	5755	0.92 (0.89, 0.94)	5981	0.95 (0.93, 0.98)	5981	0.93 (0.90, 0.95)	5868	0.92 (0.90, 0.95)	*	n/a
	15-21 days: 1st dose	6022	0.94 (0.92, 0.97)	6243	0.98 (0.95, 1.01)	6242	0.95 (0.92, 0.97)	6109	0.95 (0.92, 0.97)	*	n/a
	22-28 days: 1st dose	5897	0.91 (0.88, 0.93)	6128	0.94 (0.92, 0.97)	6127	0.92 (0.89, 0.94)	5951	0.92 (0.89, 0.94)	*	n/a
	-28 to -1 days: 2nd dose	18474	0.76 (0.74, 0.77)	18595	0.77 (0.76, 0.78)	18595	0.75 (0.74, 0.77)	16213	0.79 (0.78, 0.81)	18595	0.73 (0.71, 0.74)
	0 day: 2nd dose	258	0.29 (0.26, 0.33)	265	0.30 (0.27, 0.34)	265	0.30 (0.26, 0.33)	188	0.29 (0.25, 0.34)	265	0.29 (0.25, 0.32)
	1-7 days: 2nd dose	5126	0.83 (0.81, 0.85)	5236	0.86 (0.84, 0.89)	5223	0.84 (0.82, 0.87)	3859	0.90 (0.87, 0.93)	5236	0.82 (0.79, 0.84)
	8-14 days: 2nd dose	5814	0.95 (0.93, 0.98)	5931	0.99 (0.97, 1.02)	5910	0.97 (0.94, 0.99)	4041	1.04 (1.00, 1.08)	5931	0.94 (0.91, 0.96)
	15-21 days: 2nd dose	5902	0.99 (0.96, 1.01)	6019	1.03 (1.00, 1.06)	6003	1.00 (0.98, 1.03)	3614	1.08 (1.04, 1.12)	6019	0.97 (0.95, 1.00)
	22-28 days: 2nd dose	5699	0.97 (0.95, 1.00)	5833	1.02 (0.99, 1.05)	5809	0.99 (0.97, 1.02)	2819	1.10 (1.06, 1.14)	5833	0.97 (0.94, 0.99)

	1-28 days: 1st dose	23321	0.93 (0.92, 0.95)	24225	0.97 (0.96, 0.99)	24221	0.94 (0.93, 0.96)	23717	0.94 (0.93, 0.96)	-	-
	1-28 days: 2nd dose	22541	0.93 (0.92, 0.95)	23019	0.98 (0.96, 0.99)	22945	0.95 (0.93, 0.96)	14333	1.01 (0.99, 1.03)	23019	0.92 (0.91, 0.94)
**BNT162b2 mRNA vaccine**	Baseline	111705	1.00	89382	1.00	110087	1.00	55351	1.00	113968	1.00
	-28 to -1 days: 1st dose	12627	0.73 (0.71, 0.74)	-	-	12767	0.72 (0.71, 0.73)	11805	0.73 (0.72, 0.75)	12767	0.72 (0.71, 0.74)
	0 day: 1st dose	223	0.32 (0.28, 0.37)	232	0.30 (0.26, 0.34)	232	0.33 (0.29, 0.37)	215	0.34 (0.30, 0.39)	0	n/a
	1-7 days: 1st dose	3775	0.77 (0.75, 0.80)	3958	0.73 (0.70, 0.75)	3956	0.79 (0.76, 0.81)	3642	0.82 (0.79, 0.84)	0	n/a
	8-14 days: 1st dose	4613	0.91 (0.88, 0.93)	4837	0.88 (0.85, 0.91)	4834	0.92 (0.90, 0.95)	4452	0.95 (0.92, 0.98)	0	n/a
	15-21 days: 1st dose	4787	0.92 (0.89, 0.95)	4971	0.91 (0.88, 0.94)	4960	0.93 (0.90, 0.95)	4625	0.96 (0.93, 0.99)	0	n/a
	22-28 days: 1st dose	4429	0.88 (0.86, 0.91)	4593	0.89 (0.86, 0.92)	4582	0.89 (0.86, 0.92)	4247	0.92 (0.89, 0.95)	0	n/a
	-28 to -1 days: 2nd dose	14913	0.76 (0.74, 0.77)	15010	0.74 (0.73, 0.76)	15010	0.75 (0.74, 0.77)	14499	0.82 (0.80, 0.84)	15010	0.73 (0.71, 0.74)
	0 day: 2nd dose	255	0.33 (0.29, 0.37)	257	0.32 (0.28, 0.36)	257	0.33 (0.29, 0.37)	244	0.35 (0.31, 0.40)	257	0.31 (0.28, 0.36)
	1-7 days: 2nd dose	4619	0.85 (0.82, 0.87)	4737	0.84 (0.81, 0.87)	4712	0.86 (0.83, 0.88)	4405	0.93 (0.90, 0.96)	4737	0.83 (0.80, 0.85)
	8-14 days: 2nd dose	5158	0.94 (0.91, 0.96)	5288	0.94 (0.91, 0.97)	5257	0.95 (0.92, 0.98)	4708	1.05 (1.01, 1.08)	5288	0.92 (0.89, 0.95)
	15-21 days: 2nd dose	5365	0.98 (0.95, 1.01)	5495	0.98 (0.96, 1.01)	5477	0.99 (0.97, 1.02)	4716	1.10 (1.06, 1.13)	5495	0.96 (0.94, 0.99)
	22-28 days: 2nd dose	5281	0.97 (0.95, 1.00)	5427	0.99 (0.96, 1.01)	5409	0.99 (0.97, 1.02)	4393	1.11 (1.07, 1.14)	5427	0.96 (0.94, 0.99)
	1-28 days: 1st dose	17604	0.88 (0.86, 0.89)	18359	0.87 (0.85, 0.89)	18332	0.89 (0.87, 0.91)	16966	0.92 (0.91, 0.94)	-	-
	1-28 days: 2nd dose	20423	0.94 (0.92, 0.95)	20947	0.94 (0.93, 0.96)	20855	0.95 (0.94, 0.97)	18222	1.04 (1.02, 1.06)	20947	0.92 (0.91, 0.94)

**mRNA-1273 vaccine**	Baseline	1221	1.00	123	1.00	1193	1.00	557	1.00	1221	1.00
	-28 to -1 days: 1st dose	184	0.89 (0.77, 1.05)	-	-	184	0.91 (0.78, 1.06)	73	0.92 (0.72, 1.19)	184	0.90 (0.77, 1.05)
	0 day: 1st dose	9	1.31 (0.68, 2.52)	9	1.08 (0.54, 2.14)	9	1.34 (0.69, 2.58)	*	0.61 (0.09, 4.34)	0	n/a
	1-7 days: 1st dose	38	0.79 (0.57, 1.10)	38	0.67 (0.46, 0.98)	38	0.82 (0.59, 1.13)	6	0.58 (0.26, 1.31)	0	n/a
	8-14 days: 1st dose	40	0.87 (0.63, 1.19)	40	0.73 (0.51, 1.06)	39	0.88 (0.64, 1.22)	11	1.31 (0.71, 2.42)	0	n/a
	15-21 days: 1st dose	43	1.01 (0.74, 1.37)	43	0.83 (0.58, 1.19)	43	1.07 (0.78, 1.45)	5	0.73 (0.30, 1.79)	0	n/a
	22-28 days: 1st dose	35	0.89 (0.63, 1.24)	35	0.73 (0.50, 1.07)	34	0.92 (0.65, 1.30)	5	1.25 (0.51, 3.10)	0	n/a
	-28 to -1 days: 2nd dose	38	0.65 (0.47, 0.91)	38	0.59 (0.39, 0.89)	38	0.67 (0.48, 0.93)	*	n/a	38	0.66 (0.47, 0.92)
	0 day: 2nd dose	1	0.55 (0.08, 3.90)	1	0.49 (0.07, 3.54)	0	n/a	*	n/a	1	0.56 (0.08, 3.99)
	1-7 days: 2nd dose	22	1.88 (1.23, 2.90)	22	1.68 (1.02, 2.76)	21	2.10 (1.35, 3.26)	*	n/a	22	1.93 (1.25, 2.97)
	8-14 days: 2nd dose	13	1.36 (0.78, 2.36)	13	1.20 (0.65, 2.20)	11	1.49 (0.82, 2.73)	*	n/a	13	1.39 (0.80, 2.42)
	15-21 days: 2nd dose	6	0.82 (0.37, 1.84)	6	0.72 (0.31, 1.67)	6	1.22 (0.54, 2.75)	*	n/a	6	0.84 (0.37, 1.89)
	22-28 days: 2nd dose	7	1.39 (0.66, 2.95)	7	1.20 (0.54, 2.64)	6	2.14 (0.95, 4.86)	*	n/a	7	1.42 (0.67, 3.02)
	1-28 days: 1st dose	156	0.89 (0.75, 1.05)	156	0.74 (0.57, 0.95)	154	0.92 (0.77, 1.09)	27	0.90 (0.60, 1.36)	-	-
	1-28 days: 2nd dose	48	1.43 (1.06, 1.93)	48	1.26 (0.85, 1.87)	44	1.75 (1.27, 2.41)	*	n/a	48	1.46 (1.08, 1.98)

*****Sensitivity 1: excluding those who died from the outcome; Sensitivity 2: restricting analysis to the period post-vaccination, without censoring at death, Sensitivity 3: restricting the study period until the 1 August 2021, Sensitivity 4: restricting the study period until the 17 May 2021, when CDC announced cases of myocarditis after BNT162b2 vaccine, Sensitivity 5: removing patients who had outcomes in the 28 days after 1 dose, but before second dose.

Supplementary table 8b: Incidence rate ratios (IRR 95% CI) for single outcomes in pre-defined risk periods immediately before and after exposure to vaccination, adjusted for calendar time from December 1 2020 to August 24 2021. Comparisons between different sensitivity analyses (cells with < 5 are suppressed) to compare different choices of pre-risk length.

		Main		Sensitivity 6*		Sensitivity 7*		Sensitivity 8*	
		events	IRR (95% CI)	events	IRR (95% CI)	events	IRR (95% CI)	events	IRR (95% CI)
**Myocarditis**									
**ChAdOx1 vaccine**	Baseline	550	1.00	689	1.00	649	1.00	366	1.00
	-28 to -1 days: 1st dose	81	0.74 (0.58, 0.95)	-	-	22	0.42 (0.28, 0.65)	208	0.94 (0.77, 1.14)
	0 day: 1st dose	*	0.54 (0.14, 2.19)	*	0.61 (0.15, 2.45)	*	0.54 (0.13, 2.16)	*	0.56 (0.14, 2.27)
	1-7 days: 1st dose	47	1.76 (1.29, 2.42)	47	1.97 (1.45, 2.69)	47	1.75 (1.28, 2.39)	47	1.82 (1.31, 2.53)
	8-14 days: 1st dose	35	1.22 (0.85, 1.74)	35	1.36 (0.96, 1.94)	35	1.21 (0.85, 1.73)	35	1.25 (0.87, 1.82)
	15-21 days: 1st dose	30	1.03 (0.71, 1.51)	30	1.15 (0.79, 1.68)	30	1.03 (0.71, 1.50)	30	1.06 (0.71, 1.57)
	22-28 days: 1st dose	30	1.03 (0.71, 1.51)	30	1.15 (0.79, 1.68)	30	1.03 (0.71, 1.50)	30	1.05 (0.71, 1.56)
	-28 to -1 days: 2nd dose	58	0.65 (0.49, 0.87)	-	-	18	0.41 (0.25, 0.65)	115	0.78 (0.61, 1.00)
	0 day: 2nd dose	*	0.64 (0.16, 2.56)	*	0.71 (0.18, 2.86)	*	0.64 (0.16, 2.56)	*	0.65 (0.16, 2.60)
	1-7 days: 2nd dose	13	0.60 (0.34, 1.04)	13	0.67 (0.38, 1.16)	13	0.60 (0.34, 1.04)	13	0.61 (0.35, 1.07)
	8-14 days: 2nd dose	28	1.31 (0.88, 1.93)	28	1.45 (0.99, 2.14)	28	1.31 (0.89, 1.94)	28	1.33 (0.89, 1.98)
	15-21 days: 2nd dose	19	0.91 (0.57, 1.45)	19	1.00 (0.63, 1.59)	19	0.91 (0.57, 1.45)	19	0.92 (0.58, 1.48)
	22-28 days: 2nd dose	24	1.16 (0.76, 1.76)	24	1.27 (0.84, 1.93)	24	1.16 (0.76, 1.76)	24	1.18 (0.77, 1.81)
	1-28 days: 1st dose	142	1.29 (1.05, 1.58)	142	1.45 (1.19, 1.76)	142	1.29 (1.05, 1.57)	142	1.32 (1.05, 1.66)
	1-28 days: 2nd dose	84	1.00 (0.78, 1.27)	84	1.10 (0.87, 1.41)	84	1.00 (0.78, 1.28)	84	1.01 (0.78, 1.31)
	Baseline	398	1.00	495	1.00	460	1.00	308	1.00

**BNT162b2 mRNA vaccine**	-28 to -1 days: 1st dose	49	0.75 (0.55, 1.01)	-	-	16	0.48 (0.29, 0.79)	110	0.87 (0.69, 1.10)
	0 day: 1st dose	*	1.24 (0.40, 3.88)	*	1.31 (0.42, 4.08)	*	1.22 (0.39, 3.80)	*	1.26 (0.40, 3.93)
	1-7 days: 1st dose	27	1.45 (0.97, 2.17)	27	1.52 (1.02, 2.27)	27	1.42 (0.96, 2.12)	27	1.47 (0.98, 2.21)
	8-14 days: 1st dose	23	1.23 (0.80, 1.90)	23	1.29 (0.84, 1.98)	23	1.21 (0.79, 1.86)	23	1.25 (0.81, 1.93)
	15-21 days: 1st dose	21	1.14 (0.73, 1.78)	21	1.19 (0.76, 1.86)	21	1.12 (0.72, 1.75)	21	1.15 (0.73, 1.80)
	22-28 days: 1st dose	23	1.33 (0.86, 2.04)	23	1.39 (0.91, 2.13)	23	1.30 (0.85, 2.00)	23	1.33 (0.86, 2.06)
	-28 to -1 days: 2nd dose	48	0.96 (0.70, 1.32)	-	-	19	0.73 (0.46, 1.17)	77	0.94 (0.71, 1.25)
	0 day: 2nd dose	*	n/a	*	n/a	*	n/a	*	n/a
	1-7 days: 2nd dose	23	1.75 (1.13, 2.70)	23	1.87 (1.22, 2.86)	23	1.72 (1.12, 2.65)	23	1.73 (1.11, 2.69)
	8-14 days: 2nd dose	15	1.16 (0.69, 1.97)	15	1.24 (0.74, 2.10)	15	1.15 (0.68, 1.93)	15	1.15 (0.68, 1.96)
	15-21 days: 2nd dose	14	1.14 (0.66, 1.97)	14	1.23 (0.71, 2.10)	14	1.13 (0.66, 1.94)	14	1.14 (0.66, 1.97)
	22-28 days: 2nd dose	12	1.01 (0.57, 1.82)	12	1.08 (0.61, 1.94)	12	1.00 (0.56, 1.79)	12	1.01 (0.56, 1.82)
				-	-				
	1-28 days: 1st dose	94	1.31 (1.03, 1.66)	94	1.38 (1.10, 1.74)	94	1.29 (1.02, 1.63)	94	1.32 (1.03, 1.69)
	1-28 days: 2nd dose	64	1.30 (0.98, 1.72)	64	1.38 (1.05, 1.82)	64	1.27 (0.96, 1.68)	64	1.29 (0.96, 1.73)
**mRNA-1273 vaccine**	Baseline	22	1.00	26	1.00	24	1.00	19	1.00
	-28 to -1 days: 1st dose	*	0.41 (0.09, 1.87)	-	-	*	n/a	5	0.56 (0.20, 1.56)
	0 day: 1st dose	*	n/a	*	n/a	*	n/a	*	n/a
	1-7 days: 1st dose	7	8.38 (3.53, 19.91)	7	8.71 (3.74, 20.27)	7	8.81 (3.74, 20.72)	7	7.88 (3.24, 19.14)
	8-14 days: 1st dose	*	n/a	*	n/a	*	n/a	*	n/a
	15-21 days: 1st dose	*	n/a	*	n/a	*	n/a	*	n/a
	22-28 days: 1st dose	*	n/a	*	n/a	*	n/a	*	n/a

	-28 to -1 days: 2nd dose	*	2.19 (0.45, 10.69)	-	-	*	4.85 (1.00, 23.55)	*	1.45 (0.29, 7.32)
	0 day: 2nd dose	*	n/a	*	n/a	*	n/a	*	n/a
	1-7 days: 2nd dose	*	23.10 (6.46, 82.56)	*	20.96 (6.22, 70.69)	*	25.41 (7.17, 90.06)	*	20.33 (5.55, 74.43)
	8-14 days: 2nd dose	*	n/a	*	n/a	*	n/a	*	n/a
	15-21 days: 2nd dose	*	n/a	*	n/a	*	n/a	*	n/a
	22-28 days: 2nd dose	*	n/a	*	n/a	*	n/a	*	n/a
	1-28 days: 1st dose	9	2.97 (1.34, 6.58)	9	3.02 (1.39, 6.53)	9	3.09 (1.41, 6.76)	9	2.80 (1.23, 6.37)
	1-28 days: 2nd dose	*	9.84 (2.69, 36.03)	*	8.82 (2.55, 30.47)	*	10.77 (2.97, 39.12)	*	8.76 (2.34, 32.82)
**Positive SARS-CoV-2 test**	Baseline	119	1.00	151	1.00	123	1.00	110	1.00
	-28 to -1 days: positive test	32	2.84 (1.89, 4.28)	-	-	28	4.72 (3.08, 7.21)	41	2.47 (1.68, 3.65)
	0 day: positive test	36	78.21 (52.90, 115.61)	36	66.92 (45.92, 97.52)	36	78.39 (53.20, 115.50)	36	83.72 (56.29, 124.52)
	1-7 days: positive test	68	21.08 (15.34, 28.96)	68	18.00 (13.32, 24.31)	68	20.81 (15.20, 28.49)	68	22.44 (16.22, 31.04)
	8-14 days: positive test	37	11.29 (7.70, 16.57)	37	9.62 (6.64, 13.94)	37	11.06 (7.56, 16.18)	37	11.88 (8.06, 17.52)
	15-21 days: positive test	18	5.36 (3.24, 8.89)	18	4.56 (2.78, 7.49)	18	5.35 (3.24, 8.85)	18	5.58 (3.35, 9.27)
	22-28 days: positive test	11	3.08 (1.65, 5.75)	11	2.64 (1.42, 4.89)	11	3.11 (1.67, 5.81)	11	3.17 (1.69, 5.94)
	1-28 days: positive test	134	9.76 (7.51, 12.69)	134	8.38 (6.56, 10.71)	134	9.73 (7.51, 12.62)	134	10.16 (7.76, 13.31)
**Pericarditis**									

**ChAdOx1 vaccine**	Baseline	581	1.00	703	1.00	664	1.00	390	1.00
	-28 to -1 days: 1st dose	64	0.54 (0.42, 0.71)	-	-	19	0.34 (0.21, 0.54)	170	0.78 (0.64, 0.96)
	0 day: 1st dose	*	n/a	*	n/a	*	n/a	*	n/a
	1-7 days: 1st dose	19	0.59 (0.37, 0.94)	19	0.75 (0.47, 1.18)	19	0.64 (0.40, 1.02)	19	0.59 (0.37, 0.96)
	8-14 days: 1st dose	34	1.00 (0.70, 1.43)	34	1.28 (0.90, 1.82)	34	1.10 (0.77, 1.57)	34	1.01 (0.70, 1.47)
	15-21 days: 1st dose	23	0.64 (0.42, 0.99)	23	0.82 (0.54, 1.25)	23	0.71 (0.47, 1.08)	23	0.65 (0.42, 1.00)
	22-28 days: 1st dose	26	0.71 (0.47, 1.06)	26	0.91 (0.61, 1.35)	26	0.79 (0.53, 1.17)	26	0.72 (0.47, 1.08)
	-28 to -1 days: 2nd dose	58	0.43 (0.32, 0.57)	-	-	20	0.32 (0.20, 0.50)	143	0.66 (0.53, 0.83)
	0 day: 2nd dose	*	n/a	*	n/a	*	n/a	*	n/a
	1-7 days: 2nd dose	37	1.12 (0.79, 1.58)	37	1.41 (1.00, 1.97)	37	1.21 (0.86, 1.71)	37	1.17 (0.82, 1.66)
	8-14 days: 2nd dose	29	0.89 (0.61, 1.31)	29	1.11 (0.76, 1.62)	29	0.96 (0.66, 1.41)	29	0.94 (0.64, 1.38)
	15-21 days: 2nd dose	36	1.13 (0.80, 1.60)	36	1.38 (0.98, 1.94)	36	1.21 (0.86, 1.71)	36	1.18 (0.83, 1.69)
	22-28 days: 2nd dose	15	0.49 (0.29, 0.82)	15	0.59 (0.35, 0.98)	15	0.52 (0.31, 0.87)	15	0.51 (0.30, 0.87)
	1-28 days: 1st dose	102	0.74 (0.59, 0.92)	102	0.94 (0.76, 1.17)	102	0.81 (0.65, 1.01)	102	0.74 (0.58, 0.95)
	1-28 days: 2nd dose	117	0.91 (0.73, 1.13)	117	1.12 (0.91, 1.38)	117	0.98 (0.79, 1.21)	117	0.95 (0.76, 1.20)
**BNT162b2 mRNA vaccine**	Baseline	414	1.00	491	1.00	461	1.00	321	1.00
	-28 to -1 days: 1st dose	42	0.63 (0.45, 0.87)	-	-	15	0.45 (0.27, 0.75)	94	0.71 (0.55, 0.90)
	0 day: 1st dose	*	0.74 (0.18, 2.98)	*	0.86 (0.21, 3.44)	*	0.78 (0.19, 3.12)	*	0.71 (0.18, 2.86)
	1-7 days: 1st dose	11	0.59 (0.32, 1.07)	11	0.68 (0.37, 1.24)	11	0.61 (0.34, 1.12)	11	0.56 (0.30, 1.03)
	8-14 days: 1st dose	9	0.46 (0.24, 0.90)	9	0.54 (0.28, 1.05)	9	0.49 (0.25, 0.95)	9	0.44 (0.23, 0.86)
	15-21 days: 1st dose	19	0.98 (0.62, 1.57)	19	1.15 (0.72, 1.83)	19	1.04 (0.65, 1.66)	19	0.93 (0.58, 1.50)
	22-28 days: 1st dose	20	1.02 (0.65, 1.61)	20	1.21 (0.77, 1.90)	20	1.09 (0.69, 1.72)	20	0.97 (0.61, 1.54)

	-28 to -1 days: 2nd dose	35	0.47 (0.33, 0.67)	-	-	15	0.43 (0.26, 0.73)	76	0.60 (0.46, 0.80)
	0 day: 2nd dose	*	0.99 (0.32, 3.09)	*	1.23 (0.40, 3.85)	*	1.09 (0.35, 3.39)	*	0.96 (0.30, 2.99)
	1-7 days: 2nd dose	12	0.58 (0.33, 1.04)	12	0.73 (0.41, 1.29)	12	0.64 (0.36, 1.14)	12	0.57 (0.31, 1.02)
	8-14 days: 2nd dose	16	0.80 (0.48, 1.32)	16	0.98 (0.59, 1.63)	16	0.87 (0.52, 1.44)	16	0.78 (0.47, 1.30)
	15-21 days: 2nd dose	21	1.05 (0.67, 1.64)	21	1.29 (0.83, 2.01)	21	1.14 (0.73, 1.78)	21	1.03 (0.65, 1.62)
	22-28 days: 2nd dose	26	1.34 (0.89, 2.02)	26	1.63 (1.09, 2.44)	26	1.45 (0.97, 2.17)	26	1.32 (0.88, 2.00)
	1-28 days: 1st dose	59	0.77 (0.58, 1.02)	59	0.90 (0.68, 1.19)	59	0.81 (0.61, 1.08)	59	0.73 (0.55, 0.98)
	1-28 days: 2nd dose	75	0.93 (0.72, 1.21)	75	1.15 (0.89, 1.48)	75	1.02 (0.79, 1.31)	75	0.91 (0.70, 1.20)
**mRNA-1273 vaccine**	Baseline	12	1.00	17	1.00	17	1.00	10	1.00
	-28 to -1 days: 1st dose	*	1.62 (0.52, 5.07)	-	-	*	n/a	6	1.14 (0.41, 3.19)
	0 day: 1st dose	*	n/a	*	n/a	*	n/a	*	n/a
	1-7 days: 1st dose	*	n/a	*	n/a	*	n/a	*	n/a
	8-14 days: 1st dose	*	n/a	*	n/a	*	n/a	*	n/a
	15-21 days: 1st dose	*	n/a	*	n/a	*	n/a	*	n/a
	22-28 days: 1st dose	*	n/a	*	n/a	*	n/a	*	n/a
	-28 to -1 days: 2nd dose	*	n/a	-	-	*	n/a	*	n/a
	0 day: 2nd dose	*	n/a	*	n/a	*	n/a	*	n/a
	1-7 days: 2nd dose	*	n/a	*	n/a	*	n/a	*	n/a
	8-14 days: 2nd dose	*	n/a	*	n/a	*	n/a	*	n/a
	15-21 days: 2nd dose	*	n/a	*	n/a	*	n/a	*	n/a
	22-28 days: 2nd dose	*	n/a	*	n/a	*	n/a	*	n/a

	1-28 days: 1st dose	*	1.64 (0.45, 5.94)	*	1.33 (0.39, 4.59)	*	1.21 (0.35, 4.22)	*	1.57 (0.42, 5.88)
	1-28 days: 2nd dose	*	n/a	*	n/a	*	n/a	*	n/a
**Positive SARS-CoV-2 test**	Baseline	95	1.00	124	1.00	104	1.00	88	1.00
	-28 to -1 days: positive test	29	3.57 (2.30, 5.55)	-	-	20	4.45 (2.71, 7.30)	36	2.78 (1.82, 4.25)
	0 day: positive test	11	35.04 (18.47, 66.46)	11	29.80 (15.92, 55.77)	11	33.25 (17.62, 62.73)	11	38.67 (20.30, 73.67)
	1-7 days: positive test	11	4.85 (2.56, 9.18)	11	4.10 (2.19, 7.66)	11	4.53 (2.40, 8.52)	11	5.30 (2.79, 10.06)
	8-14 days: positive test	9	3.81 (1.90, 7.63)	9	3.18 (1.60, 6.30)	9	3.53 (1.77, 7.05)	9	4.05 (2.01, 8.14)
	15-21 days: positive test	*	1.63 (0.59, 4.45)	*	1.35 (0.50, 3.66)	*	1.52 (0.56, 4.15)	*	1.67 (0.61, 4.58)
	22-28 days: positive test	*	1.15 (0.36, 3.66)	*	0.95 (0.30, 3.00)	*	1.09 (0.35, 3.46)	*	1.16 (0.37, 3.69)
	1-28 days: positive test	27	2.79 (1.80, 4.32)	27	2.33 (1.52, 3.56)	27	2.61 (1.69, 4.03)	27	2.91 (1.87, 4.53)
**Cardiac arrhythmia**									
**ChAdOx1 vaccine**	Baseline	118356	1.00	154513	1.00	139316	1.00	80393	1.00
	-28 to -1 days: 1st dose	17562	0.78 (0.76, 0.79)	-	-	7311	0.65 (0.63, 0.67)	40103	0.88 (0.87, 0.90)
	0 day: 1st dose	289	0.33 (0.30, 0.38)	289	0.37 (0.33, 0.42)	289	0.34 (0.30, 0.38)	289	0.33 (0.29, 0.37)
	1-7 days: 1st dose	5873	0.94 (0.92, 0.97)	5873	1.05 (1.03, 1.08)	5873	0.96 (0.94, 0.99)	5873	0.92 (0.90, 0.95)
	8-14 days: 1st dose	5981	0.93 (0.90, 0.95)	5981	1.03 (1.00, 1.05)	5981	0.95 (0.92, 0.97)	5981	0.90 (0.88, 0.93)
	15-21 days: 1st dose	6243	0.95 (0.92, 0.97)	6243	1.05 (1.02, 1.07)	6243	0.97 (0.95, 1.00)	6243	0.92 (0.89, 0.94)
	22-28 days: 1st dose	6128	0.92 (0.89, 0.94)	6128	1.01 (0.98, 1.04)	6128	0.94 (0.92, 0.97)	6128	0.88 (0.86, 0.91)
	-28 to -1 days: 2nd dose	18595	0.75 (0.74, 0.77)	-	-	7886	0.65 (0.63, 0.66)	34017	0.81 (0.80, 0.82)

	0 day: 2nd dose	265	0.30 (0.26, 0.33)	265	0.32 (0.29, 0.36)	265	0.30 (0.27, 0.34)	265	0.29 (0.26, 0.33)
	1-7 days: 2nd dose	5236	0.84 (0.82, 0.87)	5236	0.92 (0.89, 0.94)	5236	0.85 (0.83, 0.88)	5236	0.84 (0.82, 0.87)
	8-14 days: 2nd dose	5931	0.97 (0.94, 0.99)	5931	1.04 (1.01, 1.07)	5931	0.98 (0.95, 1.00)	5931	0.97 (0.94, 0.99)
	15-21 days: 2nd dose	6019	1.00 (0.97, 1.03)	6019	1.07 (1.04, 1.09)	6019	1.01 (0.98, 1.04)	6019	1.00 (0.98, 1.03)
	22-28 days: 2nd dose	5833	0.99 (0.97, 1.02)	5833	1.05 (1.02, 1.08)	5833	1.00 (0.97, 1.03)	5833	1.00 (0.97, 1.03)
	1-28 days: 1st dose	24225	0.94 (0.93, 0.96)	24225	1.05 (1.03, 1.06)	24225	0.97 (0.95, 0.98)	24225	0.92 (0.90, 0.93)
	1-28 days: 2nd dose	23019	0.95 (0.94, 0.96)	23019	1.02 (1.00, 1.03)	23019	0.96 (0.95, 0.97)	23019	0.95 (0.94, 0.97)
**BNT162b2 mRNA vaccine**	Baseline	113968	1.00	141745	1.00	129472	1.00	90048	1.00
	-28 to -1 days: 1st dose	12767	0.72 (0.70, 0.73)	-	-	5795	0.63 (0.61, 0.65)	25301	0.78 (0.77, 0.79)
	0 day: 1st dose	232	0.33 (0.29, 0.37)	232	0.36 (0.32, 0.42)	232	0.34 (0.30, 0.39)	232	0.32 (0.28, 0.36)
	1-7 days: 1st dose	3958	0.79 (0.76, 0.81)	3958	0.87 (0.85, 0.90)	3958	0.81 (0.78, 0.84)	3958	0.76 (0.73, 0.78)
	8-14 days: 1st dose	4837	0.92 (0.89, 0.95)	4837	1.03 (1.00, 1.06)	4837	0.95 (0.92, 0.98)	4837	0.88 (0.86, 0.91)
	15-21 days: 1st dose	4971	0.92 (0.90, 0.95)	4971	1.03 (1.00, 1.06)	4971	0.95 (0.93, 0.98)	4971	0.88 (0.85, 0.91)
	22-28 days: 1st dose	4593	0.89 (0.86, 0.91)	4593	0.99 (0.96, 1.02)	4593	0.92 (0.89, 0.95)	4593	0.84 (0.81, 0.86)
	-28 to -1 days: 2nd dose	15010	0.75 (0.74, 0.77)	-	-	6478	0.66 (0.65, 0.68)	26396	0.75 (0.74, 0.76)
	0 day: 2nd dose	257	0.32 (0.29, 0.37)	257	0.37 (0.32, 0.41)	257	0.34 (0.30, 0.38)	257	0.31 (0.27, 0.35)
	1-7 days: 2nd dose	4737	0.85 (0.83, 0.88)	4737	0.96 (0.94, 0.99)	4737	0.88 (0.86, 0.91)	4737	0.82 (0.79, 0.84)
	8-14 days: 2nd dose	5288	0.95 (0.92, 0.98)	5288	1.06 (1.03, 1.09)	5288	0.98 (0.95, 1.00)	5288	0.91 (0.89, 0.94)
	15-21 days: 2nd dose	5495	0.99 (0.96, 1.02)	5495	1.10 (1.07, 1.13)	5495	1.02 (0.99, 1.04)	5495	0.96 (0.93, 0.98)
	22-28 days: 2nd dose	5427	0.99 (0.96, 1.02)	5427	1.09 (1.06, 1.12)	5427	1.01 (0.98, 1.04)	5427	0.96 (0.93, 0.99)

	1-28 days: 1st dose	18359	0.89 (0.87, 0.90)	18359	0.99 (0.98, 1.01)	18359	0.92 (0.90, 0.93)	18359	0.85 (0.84, 0.87)
	1-28 days: 2nd dose	20947	0.95 (0.93, 0.96)	20947	1.06 (1.04, 1.08)	20947	0.98 (0.96, 0.99)	20947	0.92 (0.90, 0.93)
**mRNA-1273 vaccine**	Baseline	1221	1.00	1443	1.00	1346	1.00	929	1.00
	-28 to -1 days: 1st dose	184	0.90 (0.77, 1.05)	-	-	78	0.78 (0.62, 0.98)	457	1.17 (1.04, 1.31)
	0 day: 1st dose	9	1.32 (0.69, 2.54)	9	1.31 (0.68, 2.53)	9	1.30 (0.68, 2.51)	9	1.45 (0.75, 2.80)
	1-7 days: 1st dose	38	0.80 (0.58, 1.11)	38	0.79 (0.57, 1.10)	38	0.79 (0.57, 1.10)	38	0.89 (0.64, 1.23)
	8-14 days: 1st dose	40	0.88 (0.64, 1.21)	40	0.86 (0.63, 1.18)	40	0.87 (0.63, 1.19)	40	0.97 (0.71, 1.33)
	15-21 days: 1st dose	43	1.02 (0.75, 1.38)	43	0.99 (0.73, 1.35)	43	1.01 (0.74, 1.37)	43	1.13 (0.83, 1.53)
	22-28 days: 1st dose	35	0.90 (0.64, 1.26)	35	0.87 (0.62, 1.22)	35	0.89 (0.63, 1.25)	35	1.00 (0.71, 1.40)
	-28 to -1 days: 2nd dose	38	0.66 (0.47, 0.92)	-	-	19	0.68 (0.43, 1.08)	57	0.81 (0.61, 1.08)
	0 day: 2nd dose	*	n/a	*	n/a	*	n/a	*	n/a
	1-7 days: 2nd dose	22	1.93 (1.25, 2.96)	22	1.86 (1.21, 2.86)	22	1.92 (1.25, 2.94)	22	2.16 (1.40, 3.32)
	8-14 days: 2nd dose	13	1.39 (0.80, 2.42)	13	1.34 (0.77, 2.33)	13	1.38 (0.80, 2.40)	13	1.55 (0.89, 2.71)
	15-21 days: 2nd dose	6	0.84 (0.37, 1.89)	6	0.81 (0.36, 1.82)	6	0.84 (0.37, 1.88)	6	0.94 (0.42, 2.12)
	22-28 days: 2nd dose	7	1.43 (0.67, 3.03)	7	1.38 (0.65, 2.93)	7	1.42 (0.67, 3.01)	7	1.60 (0.75, 3.40)
	1-28 days: 1st dose	156	0.90 (0.76, 1.06)	156	0.88 (0.74, 1.04)	156	0.89 (0.75, 1.05)	156	0.99 (0.83, 1.18)
	1-28 days: 2nd dose	48	1.46 (1.08, 1.98)	48	1.41 (1.05, 1.90)	48	1.45 (1.08, 1.96)	48	1.63 (1.20, 2.21)
**Positive SARS-CoV-2 test**	Baseline	15119	1.00	21737	1.00	17222	1.00	13499	1.00
	-28 to -1 days: positive test	6618	4.82 (4.68, 4.97)	-	-	4515	5.65 (5.46, 5.84)	8238	4.11 (3.99, 4.23)
	0 day: positive test	3847	69.83 (67.32, 72.43)	3847	52.74 (50.93, 54.62)	3847	63.41 (61.17, 65.73)	3847	78.58 (75.71, 81.56)

	1-7 days: positive test	4593	11.73 (11.33, 12.14)	4593	8.82 (8.54, 9.11)	4593	10.53 (10.18, 10.88)	4593	13.09 (12.64, 13.55)
	8-14 days: positive test	2643	6.57 (6.30, 6.85)	2643	4.90 (4.71, 5.11)	2643	5.80 (5.56, 6.05)	2643	7.19 (6.89, 7.50)
	15-21 days: positive test	966	2.30 (2.15, 2.45)	966	1.71 (1.61, 1.83)	966	2.05 (1.92, 2.18)	966	2.47 (2.31, 2.64)
	22-28 days: positive test	738	1.67 (1.55, 1.80)	738	1.25 (1.16, 1.34)	738	1.51 (1.41, 1.63)	738	1.76 (1.64, 1.90)
	1-28 days: positive test	8940	5.35 (5.21, 5.50)	8940	4.03 (3.93, 4.13)	8940	4.80 (4.68, 4.93)	8940	5.79 (5.63, 5.95)

***Sensitivity 6: including pre-risk into baseline; Sensitivity 7: restricting pre-risk to two weeks; Sensitivity 8: increasing pre-risk to 8 weeks.

Supplementary table 9: Age distribution of vaccinated patients with each outcome in the study period and in the period restricted until the 17 May 20201.

	Myocarditis					
	ChAdOx1		BNT162b2		mRNA-1273	
	Complete study period	*Restricted to 17 May 2021*	*Complete study period*	*Restricted to 17 May 2021*	*Complete study period*	*Restricted to 17 May 2021*
**Number of patients**	*919*	*898*	*657*	*504*	*39*	*8*
**Age**						
Mean age (SD)	57 (45, 70)	57 (45, 70)	58 (36, 74)	64 (51, 77)	30 (22, 38)	41 (28, 47)
16-29 years	7.3 (67)	7.2 (65)	14.0 (92)	6.3 (32)	48.7 (19)	*
30-39 years	9.0 (83)	9.1 (82)	15.7 (103)	7.5 (38)	30.8 (12)	*
40+ years	83.7 (769)	83.6 (751)	70.2 (461)	86.1 (434)	20.5 (8)	62.5 (5)

Supplementary table 10: Measures of effect of vaccinations and SARS-CoV-2 infections presented in absolute terms, number needed to expose for one excess event; excess events due to exposure; excess events per 1 million exposed. Only significant increased risks were

reported over the 1-28 days post exposure. When IRR were not significant over the 1-28 days post-vaccine, absolute measures were not given.

	**IRR (95% CI) 1-28 days***	**Number needed to expose to cause one excess event (95% CI)**	**Excess events per 1,000,000 exposed (95% CI)**
**Myocarditis**			
with 1st dose of ChAdOx1	1.29 (1.05, 1.58)	649,082 (394,227 – 3,157,549)	2 (0, 3)
with 2nd dose of ChAdOx1	1.00 (0.78, 1.27)	-	-
with 1st dose of BNT162b2	1.31 (1.03, 1.66)	760577 (453,568 – 5,353,966)	1 (0, 2)
with 2nd dose of BNT162b2	1.30 (0.98, 1.72)	-	-
with 1st dose of mRNA-1273	2.97 (1.34, 6.58)	168,612 (131,832 – 442,126)	6 (2, 8)
with 2nd dose of mRNA-1273	9.84 (2.69, 36.03)	102,625 (94,830 – 146,797)	10 (7, 11)
with SARS-CoV-2 infection	9.76 (7.51, 12.69)	25,183 (24,537 – 26,077)	40 (38, 41)
**Myocarditis (< 40)**			
with 1st dose of ChAdOx1	0.92 (0.55, 1.55)	-	-
with 2nd dose of ChAdOx1	1.50 (0.88, 2.55)	-	-
with 1st dose of BNT162b2	1.83 (1.20, 2.79)	549,501 (388,931 – 1,479,310)	2 (1, 3)
with 2nd dose of BNT162b2	3.40 (1.91, 6.04)	292,742 (247,620 – 432,998)	3 (2, 4)
with 1st dose of mRNA-1273	3.89 (1.60, 9.44)	131,834 (109,515 – 261,052)	8 (4, 9)
with 2nd dose of mRNA-1273	20.71 (4.02, 106.68)	65,423 (62,853 – 82,867)	15 (12, 16)
with SARS-CoV-2 infection	4.06 (2.21, 7.45)	102,479 (89,197 – 141,044)	10 (7, 11)
**Pericarditis**			
with 1st dose of ChAdOx1	0.74 (0.59, 0.92)	-	-
with 2nd dose of ChAdOx1	0.91 (0.73, 1.13)	-	-
with 1st dose of BNT162b2	0.77 (0.58, 1.02)	-	-
with 2nd dose of BNT162b2	0.93 (0.72, 1.21)	-	-
with 1st dose of mRNA-1273	1.64 (0.45, 5.94)	-	-
with 2nd dose of mRNA-1273	n/a	-	-
with SARS-CoV-2 infection	2.79 (1.80, 4.32)	174,970 (145,934 – 253,102)	6 (4, 7)
**Cardiac arrhythmia**			
with 1st dose of ChAdOx1	0.94 (0.93, 0.96)	-	-
with 2nd dose of ChAdOx1	0.95 (0.94, 0.96)	-	-
with 1st dose of BNT162b2	0.89 (0.87, 0.90)	-	-

with 2nd dose of BNT162b2	0.95 (0.93, 0.96)	-	-
with 1st dose of BNT162b2	0.90 (0.76, 1.06)	-	-
with 2nd dose of mRNA-1273	1.46 (1.08, 1.98)	24,335 (15,547 – 103,369)	41 (10, 64)
with SARS-CoV-2 infection	5.35 (5.21, 5.50)	416 (414 - 419)	2400 (2385, 2415)

Supplementary table 11: ICD-10 codes used to identify cases with each outcome.

Composite outcomes	ICD-10 code and description
**Myocarditis**	I40 - Acute myocarditis<br>I400 - Infective myocarditis<br>I401 - Isolated myocarditis<br>I408 - Other acute myocarditis<br>I409 - Acute myocarditis, unspecified<br>I41 - Myocarditis in diseases classified elsewhere<br>I410 - Myocarditis in bacterial diseases classified elsewhere<br>I411 - Myocarditis in viral diseases classified elsewhere<br>I412 - Myocarditis in other infectious and parasitic diseases classified elsewhere<br>I418 - Myocarditis in other diseases classified elsewhere<br>I514 - Myocarditis, unspecified
**Pericarditis**	I010 - Acute rheumatic pericarditis<br>I092 - Chronic rheumatic pericarditis<br>I30 - Acute pericarditis<br>I300 - Acute nonspecific idiopathic pericarditis<br>I301 - Infective pericarditis<br>I308 - Other forms of acute pericarditis<br>I309 - Acute pericarditis, unspecified<br>I310 - Chronic adhesive pericarditis<br>I311 - Chronic constrictive pericarditis<br>I32 - Pericarditis in diseases classified elsewhere

	I320 - Pericarditis in bacterial diseases classified elsewhere<br>I321 - Pericarditis in other infectious and parasitic diseases classified elsewhere<br>I328 - Pericarditis in other diseases classified elsewhere
**Cardiac arrhythmia**	
**Atrial fibrillation and flutter**	I48 - Atrial fibrillation and flutter<br>I480 - Paroxysmal atrial fibrillation<br>I481 - Persistent atrial fibrillation<br>I482 - Chronic atrial fibrillation<br>I483 - Typical atrial flutter<br>I484 - Atypical atrial flutter<br>I489 - Atrial fibrillation and atrial flutter, unspecified<br>I48X - Atrial fibrillation and flutter<br>I471 - Supraventricular tachycardia
**AV block and conduction disorders**	I44 - Atrioventricular and left bundle-branch block<br>I440 - Atrioventricular block, first degree<br>I441 - Atrioventricular block, second degree<br>I442 - Atrioventricular block, complete<br>I443 - Other and unspecified atrioventricular block<br>I444 - Left anterior fascicular block<br>I445 - Left posterior fascicular block<br>I446 - Other and unspecified fascicular block<br>I447 - Left bundle-branch block, unspecified<br>I45 - Other conduction disorders<br>I450 - Right fascicular block<br>I451 - Other and unspecified right bundle-branch block<br>I452 - Bifascicular block<br>I453 - Trifascicular block<br>I454 - Nonspecific intraventricular block<br>I455 - Other specified heart block<br>I458 - Other specified conduction disorders<br>I459 - Conduction disorder, unspecified

**Ventricular tachycardia**	I47 - Paroxysmal tachycardia<br>I470 - Re-entry ventricular arrhythmia<br>I472 - Ventricular tachycardia<br>I479 - Paroxysmal tachycardia, unspecified
**Ventricular fibrillation**	I490 - Ventricular fibrillation and flutter
**Other cardiac cardiac arrhythmias**	I456 - Pre-excitation syndrome<br>I49 - Other cardiac arrhythmias<br>I491 - Atrial premature depolarization<br>I492 - Junctional premature depolarization<br>I493 - Ventricular premature depolarization<br>I494 - Other and unspecified premature depolarization<br>I495 - Sick sinus syndrome<br>I498 - Other specified cardiac arrhythmias<br>I499 - Cardiac arrhythmia, unspecified<br>R00 - Abnormalities of heart beat<br>R000 - Tachycardia, unspecified<br>R001 - Bradycardia, unspecified<br>R002 - Palpitations<br>R008 - Other and unspecified abnormalities of heart beat
**Coeliac**	K900 - Coeliac disease
**Anaphylaxis**	T780 - Anaphylactic shock due to adverse food reaction<br>T782 - Anaphylactic shock, unspecified<br>T805 - Anaphylactic shock due to serum<br>T886 - Anaphylactic shock due to adverse effect of correct drug or medicament properly administered
